# Cell-type specific regulator RBPMS switches alternative splicing via higher-order oligomerization and heterotypic interactions with other splicing regulators

**DOI:** 10.1093/nar/gkad652

**Published:** 2023-08-07

**Authors:** Yi Yang, Giselle C Lee, Erick Nakagaki-Silva, Yuling Huang, Matthew Peacey, Ruth Partridge, Clare Gooding, Christopher W J Smith

**Affiliations:** Department of Biochemistry, University of Cambridge, Cambridge CB2 1QW, UK; Department of Biochemistry, University of Cambridge, Cambridge CB2 1QW, UK; Department of Biochemistry, University of Cambridge, Cambridge CB2 1QW, UK; Department of Biochemistry, University of Cambridge, Cambridge CB2 1QW, UK; Department of Biochemistry, University of Cambridge, Cambridge CB2 1QW, UK; Department of Biochemistry, University of Cambridge, Cambridge CB2 1QW, UK; Department of Biochemistry, University of Cambridge, Cambridge CB2 1QW, UK; Department of Biochemistry, University of Cambridge, Cambridge CB2 1QW, UK

## Abstract

Alternative pre-mRNA splicing decisions are regulated by RNA binding proteins (RBPs) that can activate or repress regulated splice sites. Repressive RBPs typically harness multivalent interactions to bind stably to target RNAs. Multivalency can be achieved by homomeric oligomerization and heteromeric interactions with other RBPs, often mediated by intrinsically disordered regions (IDRs), and by possessing multiple RNA binding domains. Cell-specific splicing decisions often involve the action of widely expressed RBPs, which are able to bind multivalently around target exons, but without effect in the absence of a cell-specific regulator. To address how cell-specific regulators can collaborate with constitutive RBPs in alternative splicing regulation, we used the smooth-muscle specific regulator RBPMS. Recombinant RBPMS is sufficient to confer smooth muscle cell specific alternative splicing of *Tpm1* exon 3 in cell-free assays by preventing assembly of ATP-dependent splicing complexes. This activity depends upon a C-terminal IDR that facilitates dynamic higher-order self-assembly, cooperative binding to multivalent RNA and interactions with widely expressed splicing co-regulators, including MBNL1 and RBFOX2, allowing cooperative assembly of stable cell-specific regulatory complexes.

## Graphical Abstract

**Graphical Abstract:**
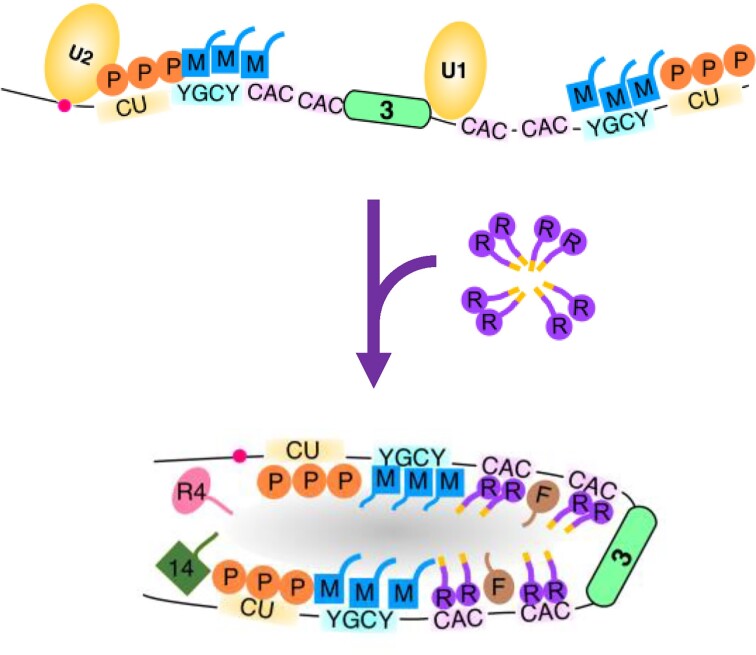

## INTRODUCTION

Alternative pre-mRNA splicing (AS) is a widespread phenomenon in eukaryotes that allows multiple transcripts to be generated from individual genes, often leading to the production of functionally distinct protein isoforms with profound effects on cell and organismal phenotype (1,2). Genome-wide studies demonstrate that most AS events (ASEs) are mediated by the combinatorial and tissue-specific binding of multiple RNA binding proteins (RBPs) (3–5). The human genome encodes at least 1500 RBPs (6,7), many of which comprise one or more RNA binding domains (RBDs) along with a variety of intrinsically disordered regions (IDRs) (8). While much focus has been placed on the role of structurally ordered RBDs in the recognition of specific RNA motifs (6), recent studies have also begun to unveil the biological significance of the IDRs (9,10).

Most splicing regulatory RBPs have preferred binding motifs, which act as splicing enhancers or silencers depending on RBP activity and motif position relative to the ASEs (11). RBPs work either synergistically or antagonistically to modulate spliceosome assembly at regulated splice sites, resulting in either exon activation or repression (12–14). Tissue-specific ‘splicing codes’ comprise different combinations of enhancer and silencer motifs along with other transcript features (11,15). Most features of cell-specific splicing codes are not unique to that cell type, reflecting the roles of widely expressed RBPs in cell-specific splicing decisions (15). Nevertheless, some splicing regulators show more restricted expression and may act as master regulators of splicing programmes (13,16). The outcome of splicing decisions can be viewed as resulting from a competition between activating and repressive inputs, while switching between AS patterns can result from modulation of either or both sets of inputs. For example, many neuron-specific exons are included as a result of reduced repression by PTBP1 combined with increased activation by RBFOX or SRRM3 proteins (15–18).

Splicing activators of the serine–arginine (SR) protein family can act by binding to exon splicing enhancers (ESEs) via their RNA recognition motif (RRM) domains, while their SR-rich IDRs either recruit core splicing factors to splice sites or stabilize interactions within splicing complexes [discussed in (19)]. Increased numbers of ESEs additively enhance splicing efficiency, but this arises from increased probability of initial weak binding to ESEs (19) or increased probability of interaction of ESE-bound SR proteins with core splicing factors (20) rather than cooperation between SR proteins bound to different ESEs. Splicing repressors broadly act in one of two ways. Their binding can directly occlude splice sites, ESEs or whole exons, blocking the binding of activating or core splicing factors (11,14). Alternatively, repressors can interact with RNA-bound core splicing factors leading to dead-end splicing complexes (21–23). Exemplified by the heterogeneous nuclear RNP (hnRNP) family, repressors have one or more RBDs and typically interact in a multivalent manner with target RNAs containing multiple cognate binding motifs. Multivalency can arise via multiple RBDs within a single protein or via oligomerization mediated by IDRs (14). The IDRs have a propensity for mediating both homomeric and heteromeric protein–protein interactions, including higher-order oligomerization and biological condensate formation, and have been shown to be functionally important in a range of splicing regulators such as RBFOX2 (24–26), hnRNPH1 (27), hnRNPA and hnRNPD (28). It has been proposed that some RBPs might act by promoting local ‘binding region condensates’ on target transcripts (29).

**Figure 1.**
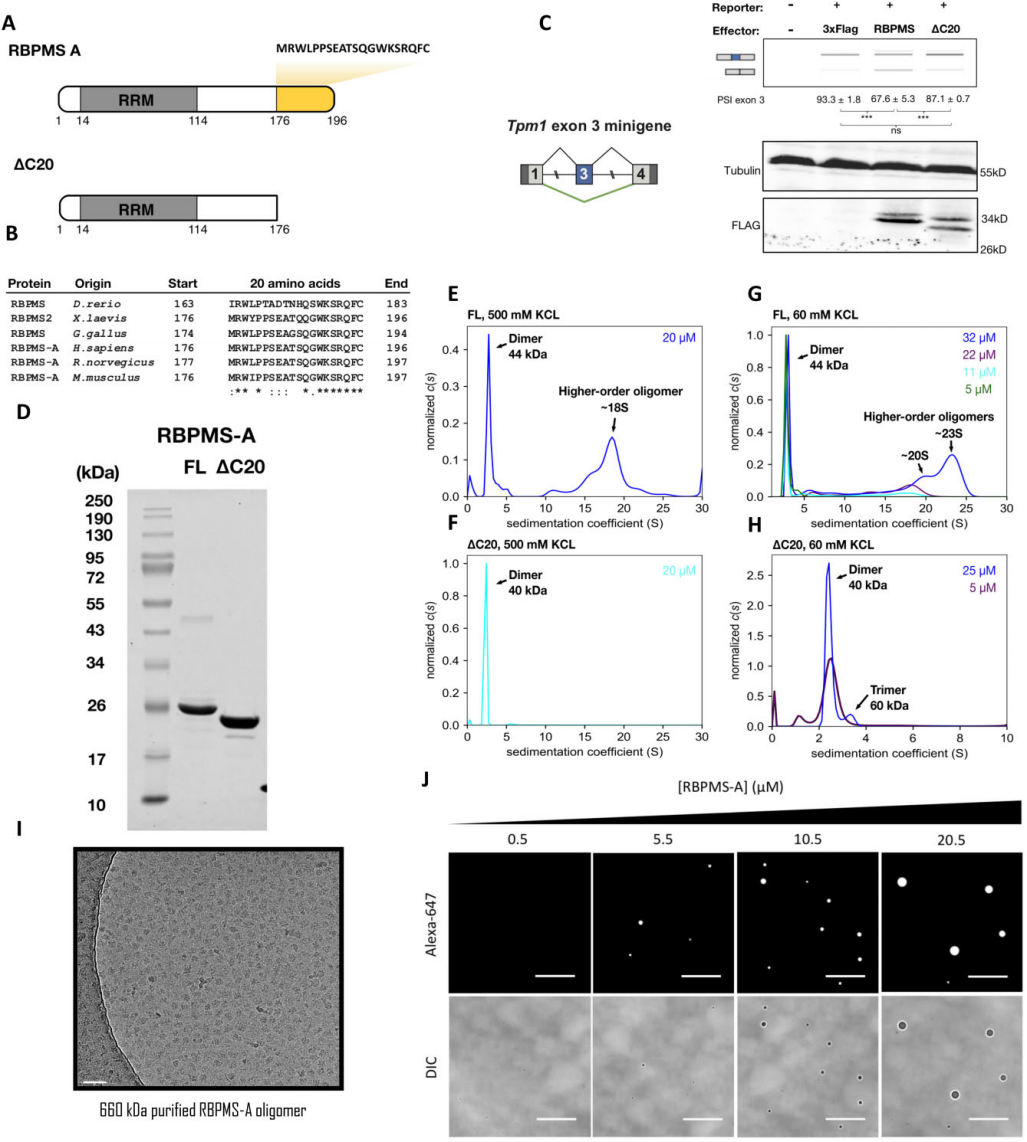
*In vitro* characterization of recombinant RBPMS. (**A**) Alternative exon encodes 20-amino acid RBPMS-A isoform specific tail (yellow). ΔC20, an experimental construct lacking the C-terminal tail. (**B**) Sequence alignment of C-terminal 20 amino acids of RBPMS-A vertebrate orthologues. Asterisk indicates fully conserved residues, colon indicates residues of strongly similar properties and period indicates residues of weakly similar properties. (**C**) Tpm1 exon 3 minigene reporter co-transfected with FLAG-tagged RBPMS in HEK293 cells. Schematic of the minigene is shown on the left. Reverse transcriptase polymerase chain reaction (RT-PCR) analysis of splicing patterns is shown above, and western blots for protein expression are shown below. Exon 3 percent spliced in (PSI) values are shown as mean ± standard deviation (SD), *n* = 3. Statistical significance from Student’s *t*-test is shown as follows: ns, *P* > 0.05; ***, *P* < 0.001. (**D**) Sodium dodecyl sulphate–polyacrylamide gel electrophoresis (SDS–PAGE) analysis of purified recombinant RBPMS-A FL and C-terminal 20 amino acids truncated (ΔC20). (**E**–**H**) Sedimentation coefficient distribution plot, *c*(*s*), of analytical ultracentrifugation (AUC) analysis using FL or ΔC20 RBPMS at 500 or 60 mM KCl, pH 7.9. Protein concentrations are colour coded. Data shown in panels (E) and (F) are normalized to the area under curve using GUSSI, while for panels (G) and (H) data are normalized to the maximum value of the dataset. (**I**) Cryo-electron microscopy (cryo-EM) image of glutaraldehyde cross-linked and purified RBPMS-A high-order oligomer of 660 kDa. Scale bar, 50 nm. (**J**) Fluorescence and differential interference contrast micrographs of His_6_-RBPMS-A droplets at 90 mM KCl. Fluorophore-conjugated RBPMS-Alexa 647 is added to 0.5 μM to give the final concentration shown. Images are representative of 5–10 acquired at each concentration. Scale bars are 25 μm.

Detailed mechanistic understanding of the action of splicing regulatory RBPs can be gained from cell-free *in vitro* investigations. For example, biochemical investigations of the *SRC* N1 exon have provided a detailed picture of how the archetypal repressor PTBP1 leads to exon skipping via cooperative binding to motifs flanking the exon (30), leading to hyperstabilized non-productive U1 snRNP binding at the N1 5′ splice site (5′ss) (22,23). Here, PTBP1 acts widely as a splicing repressor and its reduced expression in neurons leads to N1 exon inclusion. *In vitro* analyses of the action of cell-specific regulators are lacking, possibly due to challenges associated with expression and purification of active full-length (FL) proteins with extensive IDRs. We recently found that the 22-kDa RNA binding protein RBPMS is sufficient to activate a splicing programme associated with differentiated contractile vascular smooth muscle cells (VSMCs) (31). Among the ASEs regulated by RBPMS was the switch between *Tpm1* mutually exclusive exons 2 and 3, an event that has been extensively investigated using *in vitro*, *in cellulo* and *in vivo* approaches (32). *Tpm1* exon 3 inclusion results from its dominant 5′ss and 3′ss elements, which outcompete the weaker exon 2 splice sites, except in differentiated SMCs where exon 3 is repressed (33,34). MBNL and PTBP1 proteins apply a constitutive repressive influence on exon 3 by binding to flanking negative regulatory sequences (35–38). However, both proteins are widely expressed (39), and despite the binding of up to six PTBP1 and three to eight MBNL1 proteins around *Tpm1* exon 3, it is efficiently included in HeLa nuclear extract (NE) splicing reactions (35,37,38). Since RBPMS overexpression is sufficient to switch *Tpm1* splicing in cell lines such as HEK293T (31), we hypothesized that recombinant RBPMS might be able to confer tissue-specific splicing of *Tpm1* in cell-free assays. RBPMS has a single RRM that mediates both homodimerization (40,41) and binding to closely spaced pairs of CAC motifs (42,43), a 14-amino acid N-terminal tail and an ∼80-amino acid proline-rich C-terminal IDR (Figure 1A). The IDR is important for some functions (44–46) and can contribute to RNA binding (42), but the biophysical basis of its activity is unclear.

Here, we show that recombinant RBPMS confers cell-specific AS of *Tpm1* exon 3 *in vitro* by remodelling the ribonucleoprotein (RNP) complexes that assemble around the exon, thereby preventing the formation of ATP-dependent splicing complexes. The IDR is essential for RBPMS splicing regulatory function and for its ability to bind to *Tpm1* RNA in NE. It mediates higher-order oligomerization extending to liquid–liquid phase separation, cooperative binding to the multivalent *Tpm1* RNA, and interaction with other widely expressed splicing regulators such as MBNL1 and RBFOX2. In particular, the interaction with MBNL1 helps to recruit RBPMS to *Tpm1* RNA in the competitive context of NE, while RBFOX2 and other proteins are recruited by RBPMS. Notably both MBNL1 and RBFOX2 co-regulate not only *Tpm1* but also other VSMC regulated events. Our results provide an important proof of principle for how a cell-specific splicing regulator can interact functionally and physically with more widely expressed regulators to direct their activity towards a co-regulated set of ASEs.

## MATERIALS AND METHODS

### Cloning

The cloning of rat RBPMS-A cDNA, NCBI accession code: XM_006253240.2, into the pEGFP-C1 vector was described previously (31). To produce RBPMS-A with an N-terminal removable His_6_ tag, PCR products of pET15b vector and TEV-RBPMS-A generated by primers (Supplementary Table S1) were treated with T4 DNA polymerase and joined by ligation-independent cloning. Based on the resulting pET15b-TEV-RBPMS-A plasmid, PCR product generated by primers (Supplementary Table S1) was used to replace the FL RBPMS open reading frame flanked by SalI and XhoI restriction sites, producing pET15b-TEV-RBPMS-A-ΔC20 plasmid. To produce RBPMS constructs for affinity purification, the segment of pET15b-TEV-RBPMS-A flanked by XbaI and SalI sites was replaced with DNA oligonucleotide (Supplementary Table S2) encoding ribosome binding, Strep tag II and His_6_ tag sequences. Using the resulting pET15b-StrepII-His_6_-RBPMS-A, restriction enzyme cloning was conducted replacing the FL RBPMA sequence with either ΔC20 or RRM (2–114 amino acid) sequences. As tabulated in Supplementary Table S2, Gibson assembly (47) was used to produce pET15b-StrepII-His_6_-RBPMS-A-K100E.

### Expression and purification of recombinant protein

Expression vectors were transformed into *Escherichia coli* BL21(DE3) competent cells. An overnight primary culture was prepared by inoculating a single colony in 10 ml lysogeny broth (LB), 100 mg/ml ampicillin, at 37°C with shaking. The primary culture was subsequently scaled up by using a 1:50 dilution with LB (100 mg/ml ampicillin), and protein expression was induced with 0.2 mM IPTG at OD_600nm_ of 0.8 for 2 h at 37°C. The post-induction culture was harvested by centrifugation at 7000 × *g* for 10 min, and the pellet was resuspended in HisA buffer [50 mM Tris, 500 mM KCl, 40 mM imidazole, 10% (v/v) glycerol, 0.5 mM DTT, pH 8.5]. The resuspended cell pellet was lysed using a French press. Nucleic acids were precipitation on ice in HisA supplemented with 1 M LiCl and cOmplete protease inhibitor (Roche) for 10 min. Lysate was clarified by centrifugation (40 000 × *g* for 30 min), and the supernatant was filtered with 0.45 μm filter, loaded on a 1-ml Histrap HP column (Cytiva) with an AKTA purifier (Cytiva) and eluted with a gradient of increasing imidazole concentration. The identified peak fractions were buffer exchanged into QA buffer (20 mM CAPS, 50 mM KCl, pH 10), and protein concentration was determined by absorbance at 280 nm. To 1 mg of recombinant protein, 25 μg of TEV protease was added and incubated at 4°C for 16 h. Tag-free protein was purified further via a Mono Q 5/50 GL column (Cytiva), with elution via an NaCl gradient. RBPMS fractions were pooled and polished with a Superdex 200 16/600 column (Cytiva).

### Analytical ultracentrifigation

Sedimentation velocity (SV) experiments were conducted using an Optima XL-1 analytical ultracentrifuge (Beckman Coulter). Samples were loaded into standard double-sector cells, 12 mm centrepiece thickness, and analysed at a speed of 40 000 rpm with a four-hole An60 Ti rotor, at 20°C for 15 h, and 300 scans of interference optics were recorded in 90 s interval. All AUC experiments were performed in buffer containing 20 mM HEPES and 1 mM TCEP, at pH 7.9, but varied in KCl concentrations. Under 500 mM KCl condition, FL and ΔC20 RBPMS of equal molar concentration were studied at 0.46 and 0.57 mg/ml, separately. At 60 mM KCl, a concentration series of FL RBPMS was analysed from 0.7, 0.48, 0.24 to 0.11 mg/ml. Analysis of ΔC20 was conducted at either 0.5 or 0.1 mg/ml. SV data analysis was performed using SEDFIT (v14.1) program, assuming sedimentations of all species fit into a continuous *c*(*s*) model. The partial specific volume of the protein (FL_υ = 0.73 ml/g, ΔC20_υ = 0.74 ml/g) and the viscosity and density of the buffer (*ρ* = 1.016 × 10^–2^; *ρ* = 1.026) were calculated using the program SEDNTERP (48). Best *c*(*s*) fits were determined using over 60 scans, by fixing the meniscus, partial specific volume and solvent density, but floating the frictional ratio *f*/*f*_0_, until the overall root-mean-square deviation fall between 0.005 and 0.02. *f*/*f*_0_ between 1 and 1.15 (Supplementary Figure S2A–D) was determined to be the compromised value that was used to describe both RBPMS dimer and oligomers in a single *c*(*s*) plot. *f*/*f*_0_ values above 1.4 were reached for fitting the FL RBPMS-A at 5 μM (Supplementary Figure S2E) and all ΔC20 sedimentation profiles (Supplementary Figure S2F–H).

### Cryo-electron microscopy

The sample was spotted on a Quantifoil 1.2/1.3 300 mesh Cu (10) grid (Agar Scientific), blotted and plunge frozen using a Vitrobots (Thermo Fisher Scientific). Image acquisition was carried out at the normal magnification of 92 000× using a Falcon 3 counting detector in a Talos Arctica transmission electron microscope (Thermo Fisher Scientific).

### Fluorophore labelling

Purified His_6_-TEV-RBPMS-A was exchanged to 500 mM KCl AUC buffer using a Zeba spin desalting column (7K MWCO, Thermo Fisher Scientific). Alexa Fluor 647 C2 maleimide (Thermo Fisher Scientific) was added to 10× molar excess and incubated overnight at 4°C in the dark. The reaction was quenched with excess β-mercaptoethanol and buffer exchanged to QA buffer. Using an Amicon Ultra centrifugal filter (10K MWCO; Thermo Fisher Scientific), labelled protein was repetitively concentrated until a 100 000× dilution was achieved. The amount of free fluorophore in the mixture was estimated by SDS–PAGE (Supplementary Figure S4A).

### Phase separation assays

Purified RBPMS-A was exchanged to image buffer (20 mM CAPS, KCl, 1 mM TCEP, pH 10) using a Zeba spin desalting column (7K MWCO, Thermo Fisher Scientific) and diluted as indicated. For fluorescence microscopy, the mixture included 0.5 μM labelled His_6_-TEV-RBPMS-A in QA. Phase separation was induced by addition of 100 mM HEPES (pH 7.9) to final volume of 10 μl. Additionally, polyvinyl alcohol (PVA) was added in experiments using tag-free RBPMS. The mixture was incubated at room temperature for 1 h in the dark. For microscopy, 5 μl of the mixture was spotted onto a glass slide, covered and sealed. Images were acquired using a Nikon ECLIPSE Ti microscope equipped with a 60× oil-immersion differential interference contrast objective. All images were acquired within 5 h of the time at which phase separation was induced.

### Band shift

Recombinant proteins were buffer exchanged to buffer BS (20 mM HEPES, 100 mM KCl, 0.5 mM DTT, pH 7.9). RNA substrates (Supplementary Tables S5 and S11) were transcribed using T7 RNA polymerase (Thermo Fisher Scientific). A 10 μl binding reaction contains 10 nM RNA, 25 mM HEPES, 100 mM KCl, 2 mM MgCl_2_, 0.625 mM DTT, 0.1 mg/ml bovine serum albumin (BSA), 5% glycerol and increasing concentrations of recombinant RBPMS, at pH 7.9. After 1 h incubation at 30°C, 1 μl heparin was added to a final concentration of 5 mg/ml and a 10 min additional incubation was performed at 30°C. Before gel loading, the binding reactions were chilled on ice and 2 μl of 50% (v/v) glycerol was added. Bound and free RNA was separated on a native PAGE gel, 5%, 40:1 acrylamide:bisacrylamide ratio, using TBE running buffer at room temperature. Gels were dried and visualized by autoradiography on a Typhoon FLA 9000 (Cytiva). Binding curves were fitted with specific binding with Hill slope analysis, $Y = B_{{\rm max}} \times X^h(K_{\rm d}^h + X^h)$, using Prism 9 program.

### Cell culture and NE preparation

HeLa S3 cells were cultured in suspension with a 5-l T-flask in SMEM (Thermo Fisher Scientific) supplemented with 10% foetal calf serum (Sigma–Aldrich), with constant agitation at 80 rpm at 37°C. Six to eight litres of HeLa S3 culture in log phase of the growth, at a cell density of 5 × 10^5^ cells/ml, was harvested by centrifugation in a Megafuge (Heraeus) at 2000 rpm, at 4°C for 10 min. The cell pellets were immediately washed twice with ice-cold phosphate-buffered saline, in total, 40 times the cell pellet volume. The downstream extract preparation was carried out strictly according to the S10 protocol detailed in (49).

### *In vitro* transcription

Depending on the experiment, either [α-^32^P] CTP or UTP (Perkin-Elmer) labelled RNA transcript (specified in the figure legends) was transcribed from linearized pGEM vectors (Supplementary Table S7) with T7 polymerase. To make RNA for *in vitro* splicing, complex assembly and UV cross-linking assays, GTP to m7G(5′)ppp(5′)G dinucleotide cap analogue ratio was kept at 1:8 to ensure high capping efficiency. For electrophoretic mobility shift assays (EMSAs), the addition of cap analogue was omitted from the *in vitro* transcription mixture. The reaction mixtures are tabulated in Supplementary Table S11.

### *In vitro* splicing

*In vitro* splicing was carried out as in (33). Standard reactions were assembled in 10 μl with 20 fmol [^32^P]-labelled RNA transcript, 2.2 mM MgCl_2_, 0.5 mM ATP, 20 mM creatine phosphate (Roche), 16 U RiboLock RNase inhibitor (Thermo Fisher Scientific), 12 mM HEPES (pH 7.9), 12% (v/v) glycerol, 60 mM KCl, 0.12 mM EDTA, 0.3 mM DTT, 2.6% PVA and 30% (v/v) NE. RBPMS was added at the indicated concentrations in buffer BS. When the effect of RBPMS concentration was studied, 500 ng total protein (RBPMS + BSA) (NEB) was added to each reaction. Titration of RNA oligonucleotide 3×YGCY ‘D12’ (37) or 3×YCGY ‘NCD12’ (Supplementary Table S5) was conducted to study the co-regulatory activity of MBNL proteins. Splicing reactions were incubated for 3 h. After the reaction, reactions were stopped by performing protease K (PK; Thermo Fisher Scientific) digestion. RNA components were phenol extracted, ethanol precipitated and resolved on 4% denaturing urea–PAGE acrylamide gel. Splicing products were detected by autoradiography with a phospho-imaging screen and imaged with a Typhoon FLA9000 (Cytiva) imager.

### Complementary DNA oligo directed RNase H breakdown of U1 and U2 snRNPs

DNA oligonucleotides (Supplementary Table S4) complementary to 5′ end (nt 1–15) of U1 snRNA, to the branch point recognition sequence (nt 18–42) of U2 snRNA and to GAPDH mRNA were added in combination with RNase H (NEB) to NE. In sham conditions, enzyme storage buffer or H_2_O was used. The targeted digestion of snRNA was performed as described previously (50,51). Treated 20 μl NE aliquots were used directly or stored at −80°C.

### Complex assembly

Pre-spliceosomal complexes (10 μl) were assembled on 2.5 nM of [^32^P]-labelled pre-mRNA with 50% (v/v) HeLa NE, based on the standard *in vitro* splicing reaction conditions. Deviations from standard conditions are indicated in the figure legends. Reactions were incubated at 30°C for 10 min or as indicated. After complex formation, an additional 10 min incubation was performed with heparin added to the final concentration of 0.5 mg/ml. Similar to the band shift assay, the reactions were chilled on ice, to which 2 μl of 50% (v/v) glycerol was added. Complexes were loaded on a pre-run of native PAGE gel, 4%, 80:1 acrylamide:bisacrylamide ratio, using 50 mM Tris–glycine (pH 8.8) running buffer, running at 160 V at room temperature for 5 h. Gels were dried on a filter paper, and autoradiography was performed as described above.

### Protein–RNA UV cross-linking and immunoprecipitation

Twenty microlitres of pre-spliceosomal complexes subjected to UV cross-linking were assembled on 2 nM [^32^P]-labelled RNA transcript without PVA, in otherwise identical fashion to those resolved on native gels. After complex assembly and incubation with heparin, reactions were radiated with 2 × 960 mJ 240 nm UV-C light. Non-cross-linked RNA was digested by 8 μg RNase A and 0.024 U RNase T1 at 37°C for 12 min. For immunoprecipitation, RNase-treated sample was incubated with 90 μl NETS buffer [10 mM Tris–HCl, 100 mM NaCl, 10 mM EDTA, 0.2% SDS (w/v), pH 7.4] and 5 μl of antibody or pre-immune serum. After 1 h incubation at 4°C, pull-down was performed with 100 μl pre-blocked [NETS buffer with 4 mg/ml BSA (NEB) and 2 mg/ml tRNA (Sigma–Aldrich)] 0.2% protein G slurry (Cytiva). Following 1 h incubation at 4°C, protein enriched on the beads was washed (3× NETS buffer via centrifugation at 1000 × *g* for 1 min) and released by 30 μl of reducing Laemmli loading buffer. Protein–RNA cross-links were resolved on 15% SDS–PAGE gels and visualized by autoradiography.

### Psoralen RNA–RNA cross-linking and snRNA identification

Pre-spliceosomal complexes (2.5 fmol, 10 μl) were assembled on [^32^P]-labelled RNA transcript as described above. Psoralen-AMT (1 μl; Sigma–Aldrich) was added to the final concentration of 25 μl/ml, heparin omitted. After a further 10 min incubation, complexes were radiated with UV-A light for 20 min; both steps were performed on ice. The total RNA content was harvested by standard PK digestion followed by ethanol precipitation with GlycoBlue co-precipitant (Thermo Fisher Scientific). The precipitated RNA pellets were resuspended in H_2_O. Targeted RNase H (NEB) digestions were performed according to the manufacturer’s protocol. Three DNA oligonucleotides were used to verify the substrate RNA cross-linking to snRNA, and complementary DNA oligonucleotide to GAPDH mRNA was used as a negative control (Supplementary Table S4). The digested RNA was purified via standard phenol extraction procedure, ethanol precipitated and analysed on a 4% denaturing urea–PAGE acrylamide gel. The cross-linking products and sensitivity to complementary DNA oligonucleotides were determined by autoradiography with a phospho-imaging screen and imaged with a Typhoon FLA9000 imager.

### *trans*-Splicing

Transcription templates for AML_E1 and TM4_40exU1 pre-mRNA were generated by oligonucleotide synthesis and cloned into pGEM-4Z vector (Supplementary Table S5). Pre-mRNA substrates used in the *trans*-splicing assay were *in vitro* transcribed with T7 RNA polymerase (Supplementary Table S11), 80% capped with m7G cap analogue (NEB) and treated with DNase turbo (37°C, 30 min; Thermo Fisher Scientific). After column purification with RNA Monarch kit (NEB), the concentration of RNA transcripts was determined by UV absorbance. *trans*-Splicing reaction condition was similar to that of *cis*-splicing condition described previously, except splicing was performed concurrently with 5 nM of regulated (TM3 or TM23) and 50 nM constitutive (AML_E1 or TM4_40ex) RNA substrates, at 3.6 mM MgCl_2_ and 2 mM ATP. After incubation, spliced products were phenol extracted from the PK digestion and ethanol co-precipitated with 20 μg of GlycoBlue (Thermo Fisher Scientific). Ten microlitres of RNA product dissolved in water was pre-incubated (65°C, 5 min) with 20 pmol RT primer (Supplementary Table S6) and dNTP, cooled on ice and then reverse transcribed with SuperScript II reverse transcriptase (Invitrogen) according to the manufacturer’s instructions. Ten percent of the RT reaction was used as the template in 25 μl PCR reactions containing 1.25 U of JumpStart Taq Polymerase (Sigma, D9307), 1× PCR buffer (Sigma, P2192), 400 nM of primers (Supplementary Table S6) and 0.2 mM dNTP. The reactions were heated (94°C, 3 min) before 32 amplification cycles (94°C for 30 s, 60°C for 30 s and 72°C for 60 s) and a final extension (72°C, 60 s). PCR products were subsequently resolved on the QIAxcel Advanced system (QIAGEN) using a DNA screening capillary electrophoresis cartridge.

### Affinity enrichment of pre-spliceosomal proteome

RNA-assisted pull-down was adapted from (52) and performed under eight conditions; each contains three technical repeats as detailed in Supplementary Figure S13B. Before purification, *in vitro* transcribed TM3–MS2 RNA was heated at 80°C for 2 min and refolded at room temperature for 5 min in buffer RB (20 mM HEPES, 0.2 mM EDTA, pH 7.9). MS2–MBP protein (MS2 bacteriophage coat protein–maltose binding protein fusion) binding was performed at room temperature for 15 min at a protein to RNA ratio of 20:1. After thawing, a centrifugation (17 000 × *g*, 5 min) of the HeLa NE was performed to remove aggregation. A 400 μl binding reaction was constituted, in condition similar to that of the *in vitro* splicing reaction, with 200 μl of clarified NE, ±5 nM RNA, ±1.5 μM recombinant RBPMS, ±0.5 mM ATP, ±20 mM creatinine phosphate, 3 mM MgCl_2_, 70 mM KCl, PVA omitted and pH 7.9. After incubation for 20 min at 30°C, the binding reaction was added to 200 μl of 5% (v/v) amylose beads (NEB) pre-blocked overnight with 1 mg/ml BSA, 0.5 mg/ml tRNA in 20 mM HEPES, 70 mM KCl and pH 7.9. Four washes were conducted following 1 h incubation at 4°C, using WB-50 buffer (20 mM HEPES, 50 mM KCl, 0.5 mM DTT, pH 7.9). Elution was carried out using 10× bead volume of WB-50 buffer supplemented with 40 mM maltose (Sigma–Aldrich).

### Affinity enrichment of RBPMS proteome

Purified StrepII-His_6_-RBPMS-A protein was exchanged into buffer BS using a Zeba spin desalting column (7K MWCO, Thermo Fisher Scientific). Pull-down assays were assembled as 166 μl reactions containing 60% HeLa NE, 2 μM StrepII-His_6_-RBPMS, 2.2 mM MgCl_2_ and 2.6% PVA. Where applicable, HeLa NE was pretreated with 5 U/ml Benzonase (Millipore) at 30°C for 15 min and clarified by centrifugation (17 500 × *g*, 5 min) prior to the addition of RBPMS. Reactions were incubated at 30°C for 15 min, added to 200 μl of 2.5% (v/v) MagStrep ‘type3’ XT beads (IBA Lifesciences) pre-blocked overnight with 1 mg/ml BSA (NEB) and 0.5 mg/ml tRNA (Sigma–Aldrich) in WB-150 buffer (20 mM HEPES, 150 mM KCl, 0.5 mM DTT, pH 7.9), and further incubated at 4°C for 1 h. After removal of the flow-through, beads were washed six times with WB-150 buffer (6 × 1 ml). The elution was carried out at room temperature with shaking for 30 min by adding 45 μl of elution buffer (100 mM Tris, 150 mM NaCl, 1 mM EDTA, 50 mM biotin, pH 8).

### Mass spectrometry

Liquid chromatography–tandem mass spectrometry (LC–MS/MS) was used to identify and quantify proteins recovered from RNA assisted pull-down. Eluate was trichloroacetic acid precipitated, redissolved in reducing Laemmli loading, separated by SDS–PAGE and visualized with silver staining (Supplementary Figure S13C). The serial gel slices were excised and digested *in situ* with trypsin. The extracted tryptic peptides were analysed using Q-Exactive mass spectrometer. Raw data were processed using Proteome Discoverer v2.3 (Thermo Fisher Scientific). Protein identification was conducted by searching human database downloaded in 2020, UniPort, using Mascot algorithm. This generated a list of 1081 entries containing common contaminant proteins (human keratins, heat shock proteins and BSA), which were identified and removed from downstream analysis. The data obtained from Proteome Discoverer were abundance data at the peptide level. Data were processed with R package and filtered to remove entries that only identified one out of three replicates of at least one condition. The resulting 978 entries (Supplementary File RNA_MS_A) were background corrected and normalized by variance stabilizing transformations. Inspection of the list revealed repetitive interpretation due to isoforms of the same protein and searching multiple databases. We collapsed the repetitive isoform entries of the same protein and shortlisted 178 unique identifications for further analysis (Supplementary File RNA_MS_B). Low-intensity missing values were biased to no RNA background samples and no RBPMS added conditions. To conduct the differential expression analysis, missing total precursor intensity was imputed using random draws from a Gaussian distribution centred around a minimal value, *q*th quantile = 0.01. We used R package Limma to test the significant changes between background subtracted groups as tabulated in Supplementary Figure S12B. The fold changes were estimated by the Bayes method, while the adjusted *P*-values were corrected by the Benjamini–Hochberg method.

For an affinity purification–mass spectrometry (AP–MS) experiment, sample preparation, peptide identification and raw data processing were identical to those described above. Initial proteomic data processing was carried out in Scaffold (53) (Supplementary File AP_MS_TSC_raw). Entries detected in the lack of NE condition (Supplementary File AP_MS_TSC_raw, samples BL1–3) were excluded from analysis. To further enrich the list of significantly recovered proteins, total spectrum count of grouped technical repeats was compared using unpaired Student’s *t*-test. FL versus negative control produced 131 significant interactors (*P* < 0.05), while FL versus ΔC20 generated 133 significant interactors (Supplementary File AP_MS_SL). ΔC20 versus negative control produced 48 significant interactors. Control null-gene sets were generated based on protein expression levels (54). Gene Ontology (GO) analysis was performed on STRING with the following parameters adjusted: interaction sources set to experiments and databases only, and minimum required interaction score set to medium confidence (0.400).

### Glycerol gradient ultracentrifugal sedimentation

For analysis of RBPMS-A-associated protein complexes in HeLa NE, 13 ml of 10–60% (w/v) glycerol and 0–0.15% glutaraldehyde gradient (20 mM HEPES, 100 mM KCl, 2.2 mM MgCl_2_, 0.5 mM DTT, 0.2 mM EDTA, pH 7.9) was set up in 14 mm × 95 mm tubes (Beckman Coulter). Reactions (166 μl) were assembled as described previously for Benzonase-treated affinity pull-down assay, but instead of adding 2 μM StrepII-His_6_-RBPMS, a mixture of 1 μM StrepII-His_6_-RBPMS-A (repeat 1) or tag-free RBPMS-A (repeats 2 and 3) and 1 μM His_6_-RBPMS-A conjugated to Alexa 647 dye was used. Samples were loaded onto the gradient and subjected to ultracentrifugation in an SW40 Ti rotor (Beckman Coulter) at 32 000 rpm for 13 h at 4°C. Gradients were fractionated into 24 × 0.5 ml fractions. The sedimentation coefficient was deduced by analysing *Escherichia coli* lysate (55), and the 30S or 50S ribosomal subunit fractions were identified by determination of absorption at 260 nm. Fifty microlitres of each fraction was diluted with 2× transfer buffer [50 mM Tris–glycine, 16% (v/v) methanol, pH 8.8] and dot blotted onto an Immobilon-FL PVDF membrane (Millipore). Immunodetection was carried out using the antibody and dilution tabulated (Supplementary Table S10). Chemiluminescence was developed using Clarity Max ECL substrate (Bio-Rad) and imaged via Bio-Rad ChemiDoc MP imager. Imaging processing was conducted using ImageStudioLite package (LI-COR).

### PAC-1 cell culture and RNAi gene silencing

Rat pulmonary artery smooth muscle PAC-1 cells were cultured following standard procedures to maintain the differentiated state (39). siRNA-mediated knockdown was performed using reverse transfection. Briefly, 60–90 pmol siRNA and Lipofectamine RNAiMAX reagent (Thermo Fisher Scientific) were diluted by Opti-MEM without serum and then mixed and incubated at room temperature for 20 min. Diluted 2.5 × 10^5^ differentiated PAC-1 cells per well were added to the RNAi duplex–Lipofectamine RNAiMAX complexes. An siRNA of scrambled sequence ‘C2’ was used as control in every set of knockdown experiments. All siRNA sequences are tabulated in Supplementary Table S8. Each condition was repeated in ×6 format, to allow triplicates of RT-PCR analysis and sufficient material for verification of the knockdown at the protein level. In the two-hit experiment, the cells were treated again with the same reagent and procedure after 24 h. Cells were harvested 48 h after the terminal siRNA treatment.

### RT-PCR and RT-qPCR

To verify the silencing of target gene, cDNA was prepared using 1 μg of total RNA, oligo(dT) (Merck) and SuperScript II (Invitrogen) based on the instructions given by the manufacturers. RT-qPCR was performed as in (31), but using gene-specific primers (Supplementary Table S9). Two housekeeping genes were included in each analysis (CANX and Rpl32), and their geometric means were used to normalize the relative expression values. Expression values were acquired from three biological repeats.

To examine the usage of differentiation-specific exon usage, PCRs with 50 ng of cDNA were performed using the oligonucleotide primers listed in Supplementary Table S9. The PCR products were resolved in a QIAxcel system as described previously. The visualization and quantification of PSI values were conducted using QIAxcel ScreenGel software. PSI values are expressed as mean (%) ± SD. Statistical significance was examined with unpaired Student’s *t*-test.

## RESULTS

### RBPMS assembles into higher-order oligomeric structures via its C-terminus

Vertebrate RBPMS and RBPMS2 sequences show high conservation of the C-terminal 20 amino acids corresponding to the major RBPMS isoform (Figure 1A), with complete conservation of aromatic and basic residues (Figure 1B). Deletion of this region in transfected RBPMS (ΔC20) led to a significant loss in splicing repressor activity (Figure 1C), demonstrating that the RRM alone is insufficient for RBPMS splicing regulatory function, despite being sufficient for dimerization and sequence-specific binding (40,41). For *in vitro* analyses, we prepared recombinant untagged FL and ΔC20 rat RBPMS (Figure 1D), which shares 98% sequence identity with human RBPMS. Around 60% of FL RBPMS eluted as a dimer during size exclusion chromatography (SEC), consistent with previous reports (Supplementary Figure S1A) (40,41). The remaining 40% eluted as a broad peak before the 443-kDa marker approaching the void volume, indicative of heterogeneous higher-order assemblies. In contrast, 96% of the ΔC20 mutant eluted as a single peak corresponding to dimer, suggesting that the C-terminal region is required for RBPMS higher-order oligomerization. Both proteins migrated corresponding to their monomeric molecular weight on SDS–PAGE (Figure 1D).

To examine RBPMS higher-order structures in more detail, we carried out SV AUC. At high ionic strength (500 mM KCl), FL RBPMS displayed polydispersity and existed as a series of dimers and higher-order oligomers (Figure 1E). The largest oligomeric species sedimented at 18S, corresponding to a size of ∼460 kDa (∼21 monomers). In contrast, ΔC20 sedimented homogeneously as a dimeric protein (Figure 1F), consistent with its SEC profile (Supplementary Figure S1B). At low-salt conditions used for *in vitro* splicing assays (60 mM KCl), FL RBPMS oligomerized in a concentration-dependent manner with oligomers as large as 23S (∼600 kDa) observed at 32 μM (Figure 1G). In contrast, the ΔC20 mutant was mainly dimeric, with a small amount of trimers forming at 25 μM (Figure 1H). Using glycerol gradients supplemented with a glutaraldehyde cross-linker (GraFix), we found that the C-terminal tail is required for the formation of higher-order oligomers above 250 kDa, but not trimeric and tetrameric species, which were more prominent with ΔC20 (Supplementary Figure S3). FL RBPMS and ΔC20 also exhibit different friction ratios that reflect potential shape differences; FL RBPMS and ΔC20 dimers have friction ratios over 1.4 (Supplementary Figure S2E–H), whereas heterogeneous RBPMS oligomers have friction ratios of 1.0–1.15 (Supplementary Figure S2A–D). This indicates that RBPMS dimers are more elongated in conformation, whereas RBPMS higher-order structures are likely more spherical. We confirmed the spherical shape of RBPMS higher-order structures by subjecting chemically cross-linked oligomers of ∼660 kDa to cryo-EM (Figure 1I; Supplementary Figure S3). However, two-dimensional projections of RBPMS oligomers were unamenable to further classification, indicating a high degree of structural heterogeneity. Consistent with this, His_6_-tagged and tag-free RBPMS were observed to undergo liquid–liquid phase separation *in vitro* (Figure 1J; Supplementary Figure S4B). The phase transition of tag-free RBPMS required the presence of the molecular crowding reagent PVA (Supplementary Figure S4B). To examine the nature of phase-separated RBPMS droplets, His_6_-tagged RBPMS was fluorescently labelled with Alexa Fluor 647 (Supplementary Figure S4A), spiked into unlabelled RBPMS and monitored by fluorescence microscopy. Pre-formed RBPMS droplets acquired fluorescence after mixing and showed protein concentration-dependent changes in volume, suggesting that they are liquid-like and dynamic. Taken together, our biophysical analyses show that RBPMS exists as a heterogeneous mixture of concentration-dependent higher-order oligomers in addition to dimers, and that the C-terminal 20 amino acids that are important for activity *in vivo* are also essential for higher-order oligomerization.

### RBPMS confers VSMC-specific splicing decisions in HeLa NE

Overexpression of RBPMS in HEK293 cells induces skipping of *Tpm1* exon 3, dependent on tandem CAC clusters in the flanking intronic regions (31). To further examine the effects of RBPMS on *Tpm1* exon 3 splicing, we employed cell-free *in vitro* splicing assays with two different radiolabelled substrates, TM2-3-4 (33) and TM1-3-4 (56). The TM2-3-4 substrate comprises *Tpm1* exons 2, 3 and 4 and essential flanking intronic regulatory sequences (Supplementary Figure S5A). Since *Tpm1* exons 2 and 3 are mutually exclusive, TM2-3-4 provides a simple, binary 5′ss choice where exon 4 can be spliced to either exon 2 (2:4) or exon 3 (3:4). In HeLa NE, TM2-3-4 default splicing generated approximately equal amounts of 2:4 and 3:4 products (Supplementary Figure S5B, lane 6). Titration of RBPMS led to a loss of the 3:4 product, consistent with repression of exon 3 by RBPMS (Supplementary Figure S5B, lanes 5–1).

**Figure 2.**
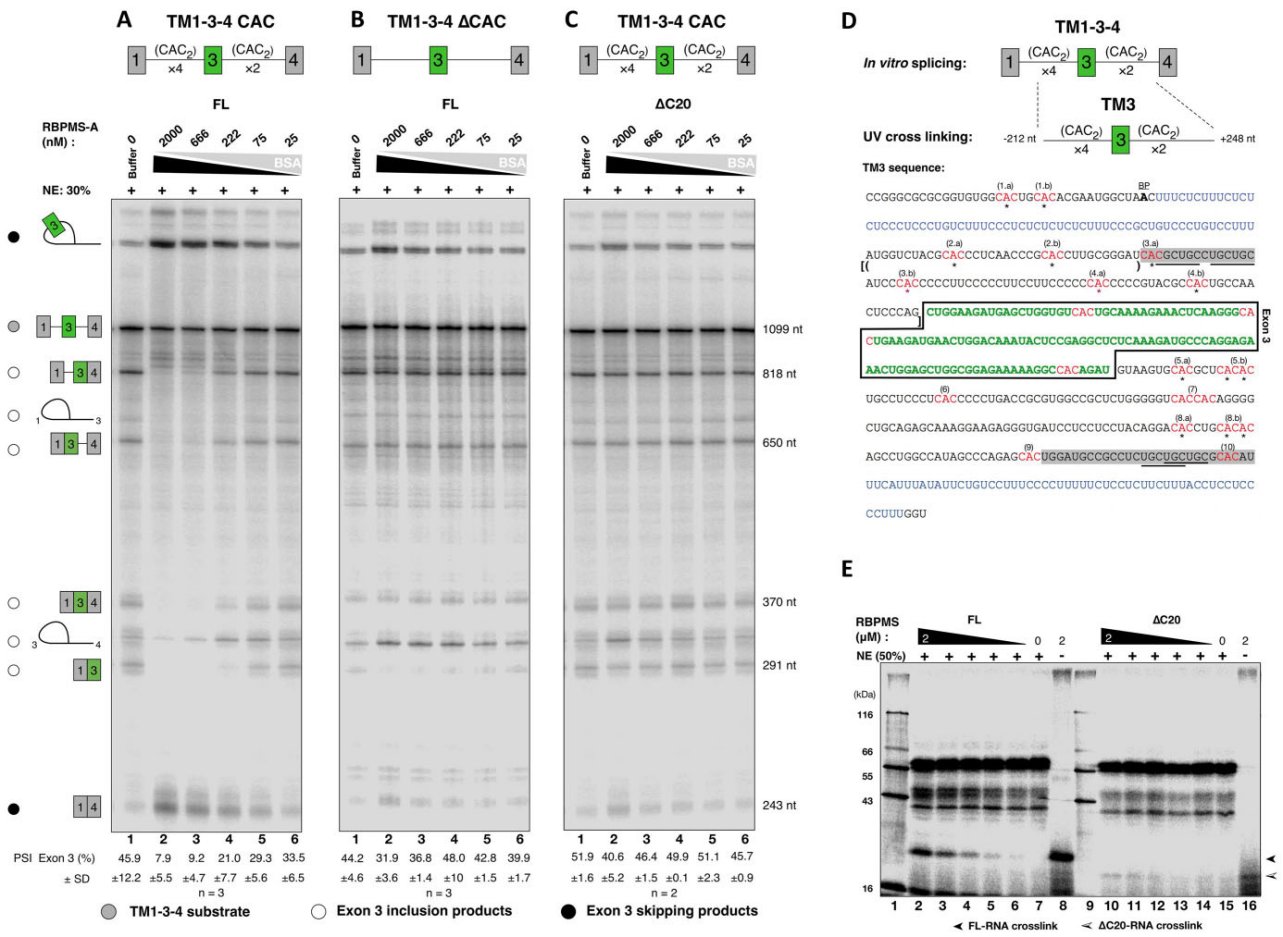
Concentration-dependent RBPMS-A modulation of TM134 *in vitro* splicing. Titration of triply diluted series of FL RBPMS-A into the *in vitro* splicing reaction (**A**) using TM134 substrate with flanking tandem CAC clusters and (**B**) using flanking CAC clusters deleted or mutated as specified in Supplementary Table S3. (**C**) Titration of ΔC20 into the *in vitro* TM134 splicing reaction. The identities of the linear spliced products are inferred by nucleotide length and depicted by schematic diagrams. The identities of the lariat are determined by matching against the previous *in vitro* splicing experiments (56) of the same substrate. The pre-mRNA substrate TM134 is indicated by a grey filled circle. 1-3-4 splicing products are indicated by open circles, while black filled circles are placed aside of the 1–4 splicing products. One representative of two to three technical repeats is shown. Size normalized quantification of exon 3 PSI values is shown as mean ± SD, *n* as indicated. (**D**) Top: the TM134-derived TM3 model RNA, containing the full complement of the flanking regulatory sequences. Bottom: the sequence of the TM3 model RNA. Exon 3 sequence is coloured in green and boxed. CAC sites are highlighted in red and numbered. C to A mutation or deletion of adenosine in ΔCAC construct was indicated by black or red asterisks. The exon 3 branch point is indicated by the bolded ‘A’. Upstream and downstream PTBP1 binding polypyrimidine tract is coloured blue. The previously determined flanking regulatory elements, URE and DRE, are shadowed in grey with MBNL binding ‘YGCY’ motifs underlined. The sequence of (CAC)_2_ or 3×(CAC)_2_ EMSA substrate is bracketed by () or [], respectively. (**E**) UV cross-linking of 3-fold dilution series of either FL or ΔC20 RBPMS to tandem CAC clusters in HeLa NE. The expected mobility of RBPMS–RNA cross-link is indicated by lanes 8 and 16 where no NE was added. One representative of two technical repeats is shown.

The TM1-3-4 substrate, comprising *Tpm1* exons 1, 3 and 4, showed even more emphatic changes in splicing outcome upon RBPMS titration (Figure 2A). The default splicing pattern of TM1-3-4 in HeLa NE is exon 3 inclusion, with bands corresponding to fully spliced 1:3:4 and partially spliced 1:3–4 and 1–3:4 intermediates (Figure 2A, empty circles). Only a very small amount of the exon skipping product (1–4) and the corresponding lariat is seen (Figure 2A, black filled circles). Strikingly, titration of FL RBPMS led to a complete switch from exon 3 inclusion to skipping, indicating that RBPMS is sufficient to confer SMC-specific splicing of *Tpm1 in vitro* (Figure 2A, lanes 6–2). When tandem CAC clusters both upstream and downstream of exon 3 were mutated, the basal pattern of splicing was unaltered but RBPMS no longer mediated exon 3 skipping (Figure 2B). Furthermore, the RBPMS ΔC20 mutant failed to induce exon 3 skipping even with intact flanking CAC clusters (Figure 2C), consistent with observations *in vivo* (Figure 1C). The cell-free AS assay therefore faithfully reflects the specificity of cell culture assays.

### RBPMS C-terminus is essential for RBPMS cooperative RNA binding

**Figure 3.**
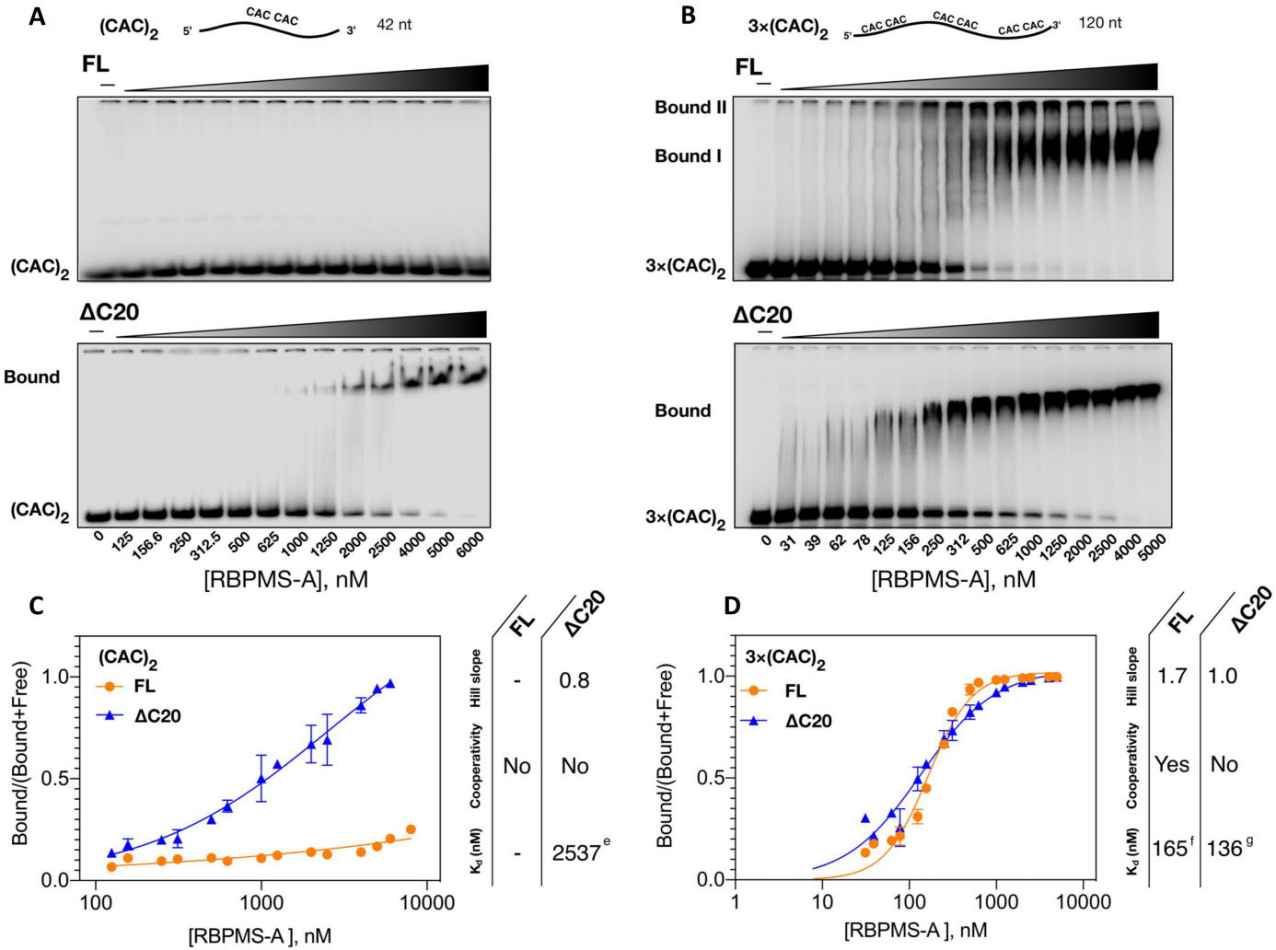
FL and ΔC20 RBPMS-A binding to (CAC)_2_ substrates in EMSA. (**A**) [^32^P-CTP]-labelled RNA of 42 nt (10 nM), containing single tandem CAC motif (10 nt spacer, Figure 2D), (CAC)_2_, was incubated with 0–6 μM recombinant RBPMS and resolved on a 5% native polyacrylamide gel. (**B**) [^32^P-CTP]-labelled RNA of 120 nt, containing three tandem CAC motifs (10–16 nt spacers, Figure 2D), 3×(CAC)_2_, was incubated with 0–5 μM recombinant RBPMS and resolved on a 5% native polyacrylamide gel. One representative of three technical repeats is shown. (**C**, **D**) Determination of dissociation constants (*K*_d_) for RBPMS binding to either (CAC)_2_ or 3×(CAC)_2_ substrates. After phosphor imaging, every lane was quantified as two proportions, ‘Bound’ and ‘Free’. The specific binding was determined as Bound/(Bound + Free), which was plotted against protein concentration. ^e^95% confidence interval (CI) 1257–18 719 nM; ^f^95% CI 152.4–177.7 nM; ^g^95% CI 118.2–159.3 nM. The data points and error bars depict the mean of three technical repeats and SD. Data points of protein at 0 nM were omitted due to the log-scaled *x*-axis.

To test whether the inability of RBPMS ΔC20 to mediate exon 3 repression was associated with altered RNA binding, we performed UV cross-linking of RBPMS along with HeLa NE proteins to a radiolabelled RNA substrate TM3, which contains exon 3 and all flanking splice site and regulatory elements (Figure 2D). While FL RBPMS efficiently cross-linked to TM3 (Figure 2E, lanes 7–2), very little cross-linking was observed for the ΔC20 mutant (Figure 2E, lanes 15–10). Hence, the C-terminal 20 amino acids are critical for RBPMS binding to the regulated *Tpm1* pre-RNA in NE and the RRM domain alone, which mediates both dimerization and RNA binding *in vitro*, is not sufficient for RNA binding in NE. We further examined the RNA binding properties of FL and ΔC20 RBPMS using EMSA. Strikingly, with an RNA substrate containing a single pair of CAC motifs, (CAC)_2_, separated by 10 nt (indicated by parentheses in Figure 2D), RBPMS ΔC20 bound with an apparent *K*_d_ of 2.5 μM, whereas FL RBPMS did not bind within the concentration range assayed (Figure 3A and C). The lack of FL binding to (CAC)_2_ may be related to the lower effective concentration of free dimeric RBPMS compared to ΔC20. In contrast, both proteins bound to a longer substrate with three tandem CAC motifs [3×(CAC)_2_, indicated by square brackets in Figure 2D], with an apparent *K*_d_ of ∼150 nM, which is 20-fold higher in affinity than ΔC20 binding to the shorter (CAC)_2_ substrate. The observed binding was dependent upon CAC motifs, as shown with CCC mutants (Supplementary Figure S6C and D). Importantly, FL RBPMS showed an additional supershifted species with limited gel mobility, which we postulate to be higher-order RBPMS-bound substrates (Figure 3B, Bound II). Indeed, a Hill factor of 1.7 (Figure 3D), derived by considering both Bound I and II complexes, suggests that FL RBPMS binds the 3×(CAC)_2_ substrate in a cooperative manner. No cooperativity was apparent (Hill factor ∼1) if only Bound I was considered (Supplementary Figure S6A and B). ΔC20 also showed no cooperativity of binding, but its affinity was similar to FL RBPMS (Figure 3B and D). These data indicate that the C-terminal 20-amino acid tail mediates cooperative binding to multivalent RNA substrates, which could be a result of its propensity for driving RBPMS oligomerization (Figure 1). However, this does not appear to be sufficient to explain the severe loss of RNA binding by ΔC20 in the competitive environment of NE (Figure 2E). Instead, we postulate that IDR-mediated interactions with other RBPs are a requirement for RBPMS to bind target RNAs effectively under splicing conditions (see below).

### RBPMS remodels pre-spliceosomal complexes on target RNA substrates

**Figure 4.**
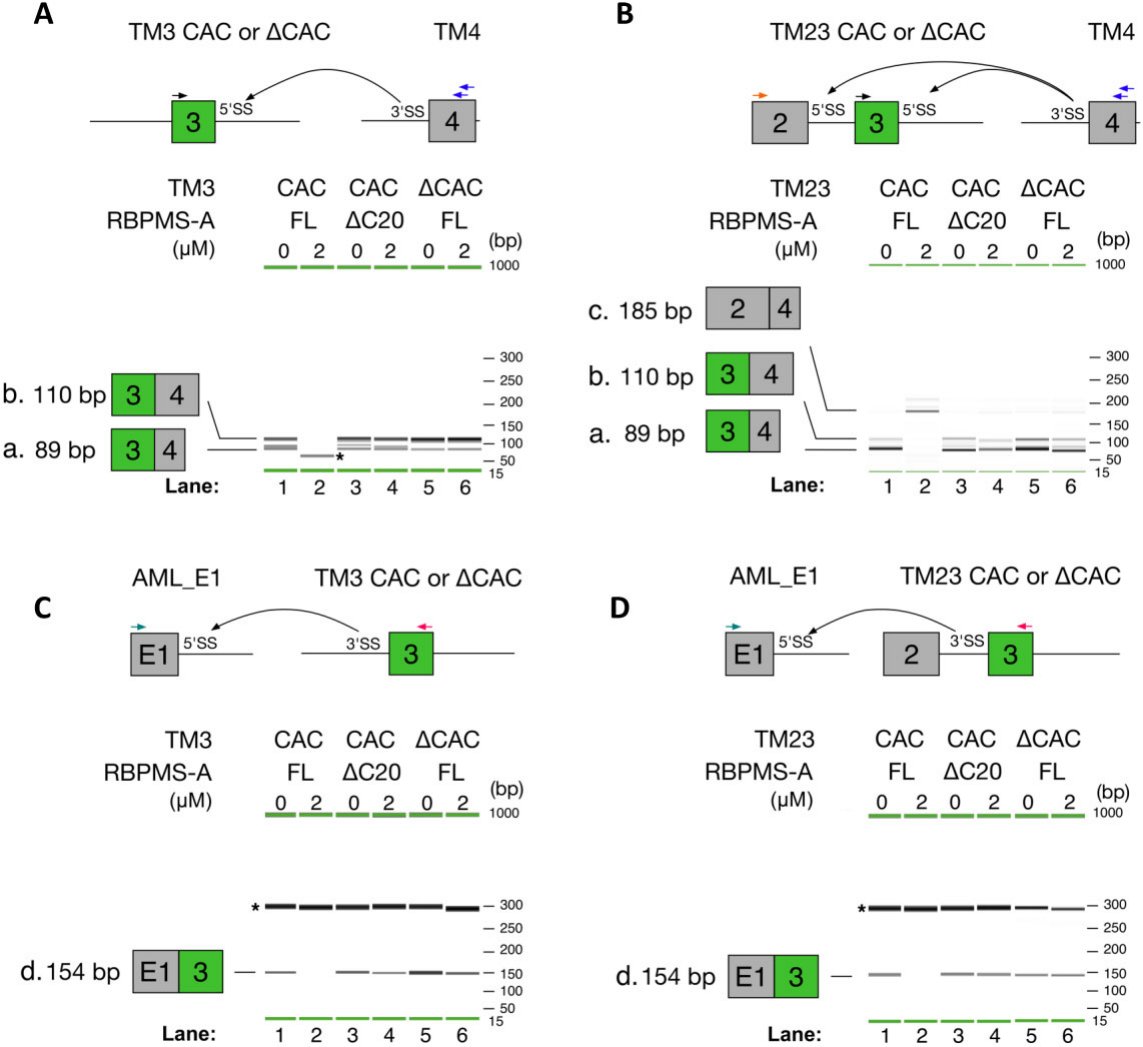
RBPMS regulates alternative *trans*-splicing. The top panels illustrate schematic diagrams showing the *trans*-splicing reactions (black curved arrows) between the TM3 (**A**) or TM23 (**B**) and the 3′ss of the TM4 *trans*-partner. Sensitivity to FL or ΔC20 RBPMS was tested on either CAC sufficient (CAC) or deficient (ΔCAC) exon 3 containing substrate. In panel (A), PCR reactions amplify 3–4 *trans*-spliced product with primers indicated by colour on top of the RNA substrates. QIAxcel imaging of PCR product a, 89 bp, is the intended 3–4 PCR product. Band b, 110 bp, results from mispriming of the reverse primer at +20 position with respect to the on-target priming. However, both products report 3–4 *trans*-splicing product faithfully. (B) Three-primer PCR reactions detect both 2–4 or 3–4 *trans*-splicing products. The identical PCR products, a and b, indicative of 3–4 spliced products were detected, while 2–4 splicing, band c, appears with FL RBPMS-A (lane 2). *trans*-Splicing of the 5′ss of the adenovirus major late (AML) exon 1 to exon 3 in TM3 (**C**) or TM23 (**D**). Sensitivity to *cis*- and *trans*-elements was tested as described above. In panels (C) and (D), the identical PCR product, d, indicative of AML_E1-3 *trans*-spliced products was detected, but subjected to FL RBPMS-A repression (lane 2). PCR primers are coloured and indicated on top of the RNA substrates. The identities of bands a–d were confirmed by sequencing. Asterisks were placed next to non-specific PCR amplifications. Extended experiments using concentration series of RBPMS and additional negative controls are shown in Supplementary Figure S7.

**Figure 5.**
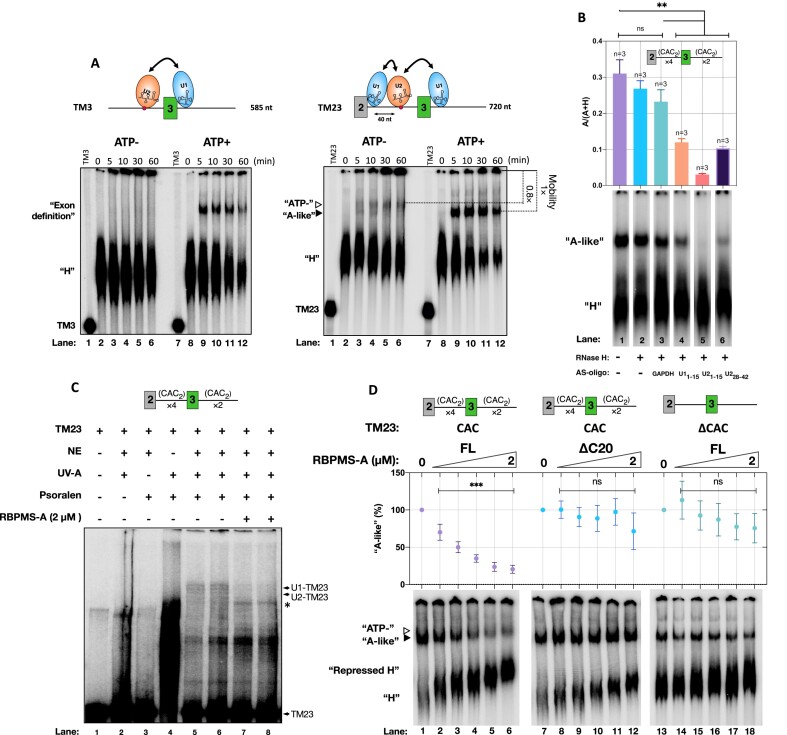
RBPMS modulates pre-spliceosome complexes on *Tpm1* exon 3 model RNAs. (**A**) RNAs, TM3 and TM23, were incubated in NE, ±ATP, and sampled at the specified time. Branch point sequence is denoted by a red dot. Hypothesized binding of snRNPs is illustrated, and the potential pairings of snRNPs are indicated by doubled-headed arrows. The mobility positions of ‘ATP-’ and ‘A-like’ complexes (described in the main text) are indicated by open and closed triangles, respectively. (**B**) ‘A-like’ complex formation in sham-treated NE (lane 1), NE pretreated with RNase H in combination with H_2_O (lane 2) or DNA oligonucleotide complementary to GAPDH mRNA, U1_1–15_, U2_1–15_ or U2_28–42_ snRNA (lanes 3–6). ‘H’ and ‘A-like’ complexes were quantitated in each lane and the proportion of A-like complex [A/(A + H)] is plotted above. (**C**) Base pairing of U1 or U2 snRNA to TM23 in ‘A-like’ complex and in the presence of RBPMS-A. The identities of snRNA–TM23 cross-links were verified and are shown in Supplementary Figure S9. Asterisk indicates a possible intramolecular cross-link that was insensitive to RNAse H digestion targeted by U1_64–75_ or U2_104–112_ (Supplementary Figure S11). (**D**) Incubation of TM23 (CAC) or CAC cluster-deficient TM23 (ΔCAC) in NE in combination with the triply diluted series of FL or ΔC20 RBPMS-A. To determine the ‘A-like’ percentage, the ratio of the ‘A-like’ complex was first determined as described in panel (A). For every titration, RBPMS spiked conditions were normalized against the condition where RBPMS-A was omitted [(‘A-like’_RBPMS_/‘A-like’_null_) × 100]. For panels (B) and (D), the data points and error bars depict the mean of three technical repeats and SD. Unpaired, one-tailed, Student’s test was used to assess the differences in mean ratio or percentage of ‘A-like’ conversion across different conditions and shown as follows: ns, non-significant; **, *P* < 0.01; ***, *P* < 0.001.

RBPMS may mediate exon 3 repression by direct modulation of pre-spliceosomal assembly pathways. To explore this possibility, we examined complex assembly on two *cis*-splicing incompetent substrates, TM3 and TM23, in HeLa NE (Figures 4 and 5). Both TM3 and TM23 contain *Tpm1* exon 3 with the full complement of splice site and regulatory elements, but no flanking constitutive splice sites, thereby allowing us to focus on complex assembly only across the regulated exon 3 region. To ensure that the complexes forming on TM3 and TM23 are functionally relevant, we first tested their activity in *trans*-splicing assays (51).

To test the 5′ss functionality of TM3 and TM23, *Tpm1* exon 4 with an 88-nt 5′ intronic extension (TM4) was used as a 3′ *trans*-splicing partner. TM4 (50 nM) was used at 10-fold molar excess over either TM3 or TM23 (5 nM), in order to overcome splicing inefficiency caused by the physical separation of 5′ and 3′ substrates (57). The 89-nt TM34 spliced product was generated from paired TM3:TM4 and TM23:TM4 reactions in HeLa NE (band a, Figure 4A and B, lane 1; Supplementary Figure S7). A second band at 110 nt (band b) resulted from mispriming 21 nt downstream in exon 4, but still reports on TM3:TM4 splicing. These results confirm that TM3 and TM23 retain 5′ss functionality in *trans*-splicing. Moreover, for both *trans*-splicing substrates, the addition of FL RBPMS abolished TM34 splicing (Figure 4A and B, lane 2). With the TM23 substrate, we included a third PCR primer to detect TM24 splicing (185 nt, band c, Figure 4B). Remarkably, addition of >75 nM RBPMS induced a complete switch from TM34 to TM24 splicing (Figure 4B, lane 2; Supplementary Figure S6B), indicating that RBPMS regulates 5′ss competition between exons 2 and 3. In other words, RBPMS mediated alternative *trans*-splicing *in vitro* (Figure 4B). This is an important observation that demonstrates the specificity of RBPMS action. The 5′ss of exon 2 is only 41 nt upstream of the exon 3 branch point; the activation of exon 2 splicing demonstrates that RBPMS is not ‘smothering’ the whole RNA to make it splicing incompetent, but is acting in a precise and targeted manner. Again, for both TM3 and TM23, RBPMS *trans*-splicing regulatory activity was completely dependent on its C-terminal 20 amino acids (lane 4) and the presence of CAC motifs flanking exon 3 (lane 6). The identities of bands a, b and c were confirmed by sequencing, and control lanes showed that they only appeared upon incubation with both *trans*-spliced partner RNAs and in the presence of ATP (Supplementary Figure S7, lanes 19–24).

We tried to test the 3′ss functionality of TM3 and TM23 using *Tpm1* exon 1 with its downstream intronic segment (TM1) as a 5′ *trans*-splicing partner. We were unable to detect the TM1:3 splice product with either the TM3 or TM23 acceptor (data not shown), despite the fact that equivalent splice products were readily detected in *cis*-splicing experiments (Figure 2). We suspected that the large size of the TM1 substrate (350 nt) might result in inefficient *trans-*splicing kinetics. We therefore used the 34-nt AML exon 1 with 95 nt of 3′ intronic sequence (AML_E1) (51). We detected AML-TM3 splice products at 154 nt, confirming the 3′ss functionalities of TM3 and TM23 (band d, Figure 4C and D). Again, RBPMS inhibited the *trans*-splicing of AML_E1 to exon 3 for both substrates (Figure 4C and D, lanes 1 and 2). This effect was again dependent on an intact RBPMS C-terminal region (lanes 3 and 4) and the presence of CAC clusters on both sides of the regulated exon (lanes 5 and 6).

Having established that both TM3 and TM23 are competent for *trans*-splicing and are regulated specifically by RBPMS, we proceeded to investigate how RBPMS regulates the assembly of splicing-related complexes (Figure 5). In the absence of RBPMS and with ATP present, both minimal substrates initially formed a heterogeneous (H) complex of fast mobility that developed into a lower-mobility complex within 5 min (Figure 5A, ATP+). The lower-mobility ATP-dependent complexes were sensitive to targeted partial digestion of U1 and U2 snRNAs (Figure 5A and B; Supplementary Figure S8A and B). Psoralen cross-linking further confirmed U1 and U2 snRNA base pairing to TM23 (Figure 5C, lanes 5 and 6; Supplementary Figure S9). Given the single exon configuration of TM3, we propose that the lower-mobility complex on TM3 corresponds to an exon definition A (EDA) complex (22). On the other hand, the advanced complex formed on TM23 could be a combination of an EDA complex across exon 3 and a sterically hindered ‘A-like’ complex between exons 2 and 3 (58). Notably, on TM23 but not TM3, a lower-mobility complex also formed in the absence of ATP (denoted by ATP−, Figure 5A, right). This complex could be distinguished from the ATP-dependent ‘A-like’ complex by its slightly lower mobility (∼0.8-fold lower mobility).

Addition of FL RBPMS abolished the formation of ATP-dependent complexes on TM3 and TM23 in a concentration-dependent manner (Figure 5D; Supplementary Figure S10A). At the highest concentration (2 μM) of RBPMS, ∼20% of lower-mobility complexes remained on TM23 (Figure 5D). However, the residual low-mobility complex migrated more slowly than the ATP-dependent complex in the absence of RBPMS (∼0.8-fold lower mobility), similar to the ATP-independent low-mobility complex (Figure 5A). RBPMS therefore appears to inhibit formation of all ATP-dependent complexes. Consistent with this, in the presence of RBPMS, U1 and U2 snRNA base pairing to TM23 was eliminated (Figure 5C, lanes 7 and 8; Supplementary Figure S11). Meanwhile, the progressive reduction in H-complex gel mobility is indicative of RBPMS binding (Figure 5D, left panel, ‘Repressed H’). All of the effects of RBPMS upon splicing complexes were dependent on the C-terminal 20 amino acids of RBPMS and clusters of tandem CAC sites flanking the regulated exon (Figure 5D, middle and right panels; Supplementary Figure S9), mirroring the requirements for RBPMS splicing regulation in *cis*- and *trans*-splicing assays (Figures 2 and 4). Taken together, our results establish a strong link between RBPMS splicing regulatory activity and its remodelling of spliceosomal complexes on model transcripts.

### RBPMS remodels the RNA-bound proteome composition

**Figure 6.**
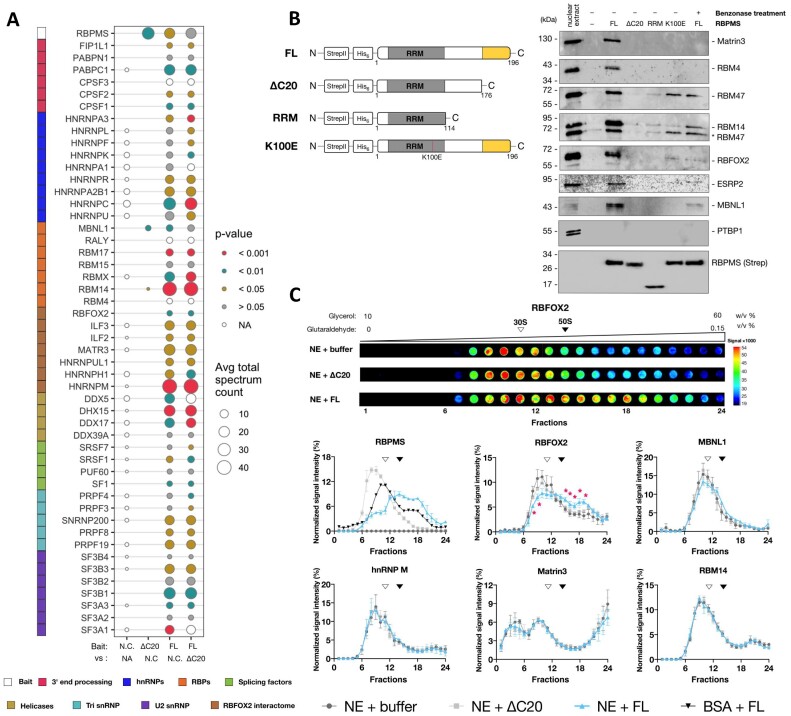
RBPMS-A interactome in HeLa NE. (**A**) Fifty shortlisted AP–MS identified interaction partners assorted by functional groups: hnRNP, heterogeneous nuclear ribonucleoprotein; RBP, RNA binding protein; Avg total spectrum count, the average number of the raw spectral counts across replicates. Statistical comparisons of the raw peptide count replicates were conducted as specified under the *x*-axis and by unpaired, one-tailed, Student’s *t*-test. *P*-values are colour coded by the significant levels. N.C. is negative control. Due to the low peptide count and variability within certain replicates, *t*-tests in these cases were inconclusive, therefore annotated as NA. (**B**) Left panel: schematic diagram of Strep tag II and His_6_-tagged RBPMS variants used. Right panel: immunoblot assessment of the dependence of RBPMS-A interactome to C-terminal truncations, an RNA binding mutation K100E and nuclease treatment. Proteins probed are indicated on the right (see the ‘Materials and Methods’ section for antibodies used). Strep-His_6_-RBPMS-A and its truncations were detected with an antibody to the Strep tag II. (**C**) Top panel: dot blot analysis of RBFOX2 sedimentation resolved by glycerol gradient loaded with nuclease-treated HeLa NE in combination with either buffer, ΔC20 or FL RBPMS. Gradient fractionations performed from top to bottom are ordered from left to right. Scale bar on the right colour coded for the signal intensities. Bottom panel: normalized signal intensity of indicated protein across fractionated glycerol gradient. Colour key underneath the panel indicates the experimental conditions. Every sedimentation profile was determined by performing two to three technical repeats of the gradient separation followed by dot blot and quantification. Intensity of an individual fraction is normalized to the total signal of a given gradient. Sedimentation position of 30S or 50S *E. coli* ribosomal subunit is indicated as open or filled triangle. Error bar indicates standard error of the mean. Statistical analysis for NE + FL versus NE + buffer was performed using unpaired, one-tailed, Student’s *t*-test, indicated as follows: *, *P* < 0.05.

Changes in gel mobility of complexes forming on TM3 and TM23 are expected to be caused not only by RBPMS binding and snRNP displacement, but also by the recruitment and displacement of other RBPs. In line with this hypothesis, FL RBPMS was observed to alter the cross-linking of other NE proteins to the TM3 substrate (Figure 2E, lanes 2–7). To identify RBPMS binding partners in HeLa NE, we first generated recombinant RBPMS proteins with an N-terminal Strep tag II followed by a polyhistidine tag (StrepII-His_6_-RBPMS) for AP–MS experiments (Supplementary Figures S12 and S14; Figure 6B). StrepII-His_6_-RBPMS has similar oligomerization properties to untagged RBPMS, indicating that the tag has negligible effects on RBPMS biophysical and functional properties (Supplementary Figure S4A). A total of 118 FL RBPMS interactors were significantly enriched above background and 100 of these interactors were significantly depleted from the ΔC20 pull-down (Supplementary Figure S14C and Supplementary File AP_MS_SL). Interactors were then classified using enriched GO terms on STRING (59). Analysis of both lists of interactors generated near-identical top five enriched terms associated with mRNA processing, RNA splicing and RNA binding from each category (biological process, molecular function and cellular component) (Supplementary Figure S15). The enriched terms were not reproduced to the same significance in three independent gene expression matched sets of 118 genes (Supplementary Figure S16; gene sets in Supplementary File AP_MS_SL). Fifty RBPMS interactors were selected from the following terms: RNA splicing, RNA binding and RNP complex, and grouped (Figure 6A) based on their annotated function as 3′-end processing factors, hnRNPs, splicing regulators, other RBPs, interaction network of RBFOX2 (24), RNA helicases, and components of pre-spliceosome complexes such as U4/U6·U5 tri-snRNP and U2 snRNP. Among the splicing regulators was MBNL1, a known regulator of *Tpm1* splicing (37). Remarkably, truncation of the C-terminal 20 amino acids led to a near-global loss of the RBPMS interactome (Figure 6A; Supplementary File AP_MS_SL).

To validate some of the RBPMS-mediated interactions, we performed western blot analysis (Figure 6B, right) and confirmed interactions between FL RBPMS and MATR3, RBM4, RBM14, RBM47, RBFOX2, ESRP2 and MBNL1. The lack of interaction with PTBP1, a known co-regulator of *Tpm1* exon 3, serves as a control for the specificity of RBPMS interactions. With the exception of RBM14 and RBM47, all of the interactions were completely abolished by the ΔC20 deletion. This included MBNL1, which did not show a statistically significant difference between FL and ΔC20 in the AP–MS analysis (Figure 6A), but clearly showed loss of binding by ΔC20 in the western blot (Figure 6B). Using Benzonase-treated NEs or the K100E RNA-binding mutant, most of the interactions were observed to be partially or completely dependent on RNA binding (Figure 6B, lanes 6 and 7 compared to lane 3).

We next tested whether RBPMS detectably altered the glycerol gradient sedimentation profiles of a subset of its interactors (Figure 6C). Upon addition to NE, RBPMS itself sedimented more rapidly than free RBPMS, indicating that it forms heterogeneous high molecular weight complexes. Consistent with its loss of both homo-oligomerization (Figure 1) and heterotypic protein–protein interactions (Figure 6A and B), the ΔC20 RBPMS in NE sedimented in lighter fractions than FL RBPMS (Figure 6C). Strikingly, RBFOX2 shifted into heavier fractions upon addition of FL RBPMS but not ΔC20 to NE (Figure 6C), suggesting that the two proteins are components of a common higher molecular weight complex. RBFOX2 is known to be present in the multicomponent Benzonase-resistant large assembly of splicing regulators (LASR) complex (24). The sedimentation profiles of other proteins, including the LASR components MATR3 and hnRNPM, were unaffected by RBPMS suggesting that the RBPMS–RBFOX2 complex is distinct from LASR. MBNL1 appeared to show a slight shift to heavier complexes (Figure 6C), but the differences in MBNL1 were not significant between equivalent fractions in the presence or absence of RBPMS.

**Figure 7.**
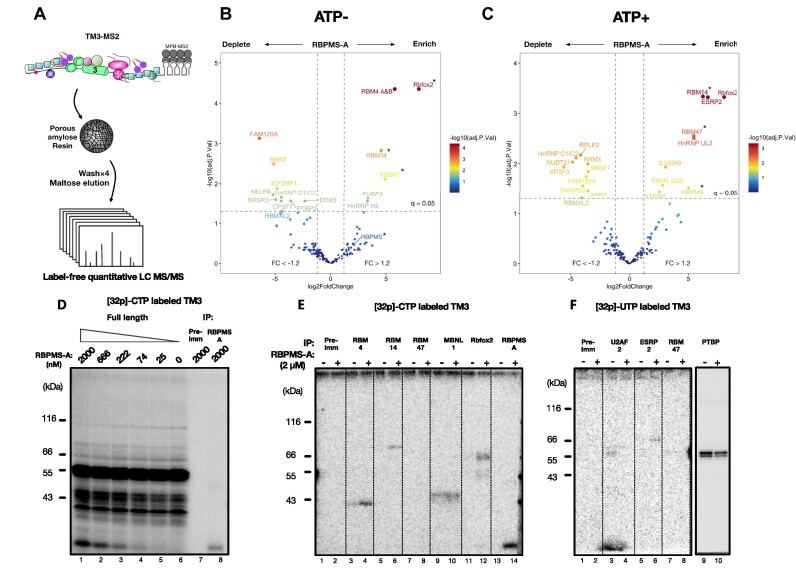
RBPMS modulates transcript-bound proteome. (**A**) Graphical summary of the RNA affinity purification. TM3–MS2 (black line), exon 3 (filled green oval) and branch point (red dot). MBP–MS2 fusion protein-bound TM3–MS2 was incubated in HeLa NE under conditions of ‘H’ complex assembly, ±RBPMS-A (1.5 μM) and ±ATP. The RBPs (coloured shapes) associated with the transcript were purified via amylose resin, washed, eluted and subjected to label-free quantitative LC–MS/MS. Volcano plots showing differential pull-down analysis of the proteins discovered from the RNA affinity purification in the absence (**B**) or presence (**C**) of ATP. Asterisk indicates that protein was not detected in the absence of RBPMS-A; the underestimated log_2_(fold change) was derived using imputed background values. Vertical dashed lines indicate log_2_(fold change) > 1.2 and <−1.2, respectively. Horizontal dashed line indicates adjusted *P*-value (false discovery rate, *q*-value) of 0.05. *P*-values were corrected by the Benjamini–Hochberg method. (**D**) Lanes 1–6: protein binding to radiolabelled TM3 transcript upon titration of FL RBPMS-A into NE with 0.5 mM ATP was analysed via UV cross-linking. Two immunoprecipitations were performed using samples prepared identically to that shown in lane 1, with either rabbit pre-immune serum (lane 7) or rabbit polyclonal antibodies specific to RBPMS (lane 8). Immunoprecipitations of RNA–protein cross-links from the digested [^32^P]-CTP (**E**) or [^32^P]-UTP (**F**) labelled TM3 transcript incubated in NE ± RBPMS-A, in the presence of ATP. Proteins probed are indicated on top using antibody listed in the ‘Materials and Methods’ section.

Having established that the RBPMS interactome includes numerous splicing factors and regulators, we proceeded to examine how RBPMS remodels the composition of splicing-related complexes on splicing substrates tagged with MS2 sites to facilitate affinity purification with MBP–MS2 (Figure 7A). We initially attempted to use the TM23 substrate, but were unable to achieve purification of specific complexes, in part due to the large size of TM23 (820 nt). We therefore opted for the shorter TM3 substrate and omitted the molecular crowding agent PVA to facilitate comparable recovery of transcripts across different experimental conditions. Under these conditions, stable association of snRNPs with the RNA is expected to be very inefficient, so we would not expect to capture the displacement of snRNPs evident in Figure 5D. However, we hoped to capture remodelling of RBPs associated with the H-complex (Figure 5D, lanes 1–6) that might influence subsequent complex assembly under splicing conditions with PVA present. Urea–PAGE analysis showed a slight increase in RNA recovery in RBPMS-spiked samples, but these differences were within the normalization range of downstream data processing (Supplementary Figure S13C). However, assembly of ATP-dependent low-mobility complexes was negligible under these conditions (Supplementary Figure S13A). Total proteins from each condition (±ATP, ±RBPMS) were submitted to LC-coupled quantitative label-free MS/MS. Consistent with the complex assembly conditions, very few snRNP proteins were detected even in the absence of RBPMS. However, differential pull-down analysis revealed that a large number of RBPs were either recruited to (e.g. ESRP2) or displaced from (e.g. SRSF3) the TM3 substrate by RBPMS (Figure 7B and C). Proteins identified as RBPMS interactors in the AP–MS experiment were also found among both RBPMS-recruited proteins (e.g. RBM4, RBM14, RBFOX2) and RBPMS-displaced proteins (e.g. SRSF7, hnRNPC) (Supplementary File RNA_MS_B). The differential recruitment of some proteins appeared to be sensitive to ATP; for example, enrichment of bound ESRP2 and depletion of SRSF7 by RBPMS were only observed in the presence of ATP. We observed no significant changes in the transcript-bound levels of PTBP1 and MBNL1, despite the fact that MBNL1 was identified as a direct interactor (Figure 6) and both proteins are co-regulators that bind to sites flanking *Tpm1* exon 3 (37,38).

Differential RBPMS-sensitive binding of selected RBPs was confirmed by UV cross-linking of [^32^P]-UTP- or [^32^P]-CTP-labelled TM3 RNA to proteins in HeLa NE (Figure 7D–F). RBM4, RBM14, Rbfox2 and ESRP2 cross-linking only occurred in the presence of RBPMS, in agreement with results from the differential pull-down using MS2-tagged TM3. Notably, each of these proteins was seen to interact with RBPMS in an RNA-dependent manner (Figure 6B). RBM47 cross-linking was not detected with either the [^32^P]-CTP- or [^32^P]-UTP-labelled transcript, possibly due to poor cross-linking efficiency. In line with the differential pull-down results, cross-linking of MBNL1 and PTBP1 was unchanged by RBPMS (Figure 7E and F, lanes 9 and 10), Therefore, PTBP1 and MBNL1 binding to TM3 does not require active recruitment, although their transcript-bound activities may still be regulated by RBPMS. Consistent with repression of *Tpm1* exon 3 splicing, cross-linking of the essential splicing factor U2AF2, which recognizes the polypyrimidine tract, was reduced by RBPMS (Figure 7F).

### RBFOX2 and MBNL1 cooperate with RBPMS in the VSMC AS programme

**Figure 8.**
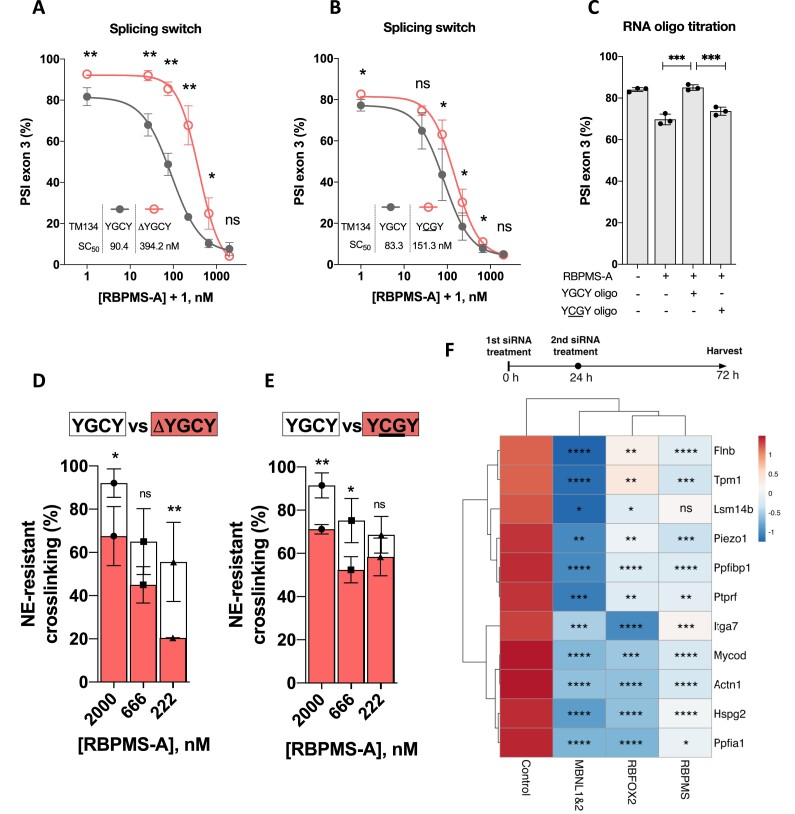
RBPMS, RBFOX2 and MBNL1 co-regulate VSMC alternative splicing events. Sensitivity of RBPMS modulated splicing switch on TM134 to the deletion (**A**) or mutation (**B**) of flanking MBNL binding sites (URE and DRE elements; Figure 2B). Half-maximum switching concentration, SC_50_, was estimated with log(inhibitor) versus response-variable slope equation provided by GraphPad Prism 9. (**C**) Titration of RNA oligo containing three YGCY or YCGY motifs to the TM134 *in vitro* splicing reactions in combination with the effect of 70 nM RBPMS-A. Comparison of RBPMS-A NE-resistant UV cross-linking to [^32^P]-CTP-labelled TM3 transcript between WT with the deletion (**D**) or mutation (**E**) of flanking MBNL binding sites. %_resistant_, the percentage of binding that sustained NE competition, (Signal_NE+/_Signal_NE−_) × 100. Statistical analysis was performed at each RBPMS concentration. (**F**) Summarizing heatmap. The effects of knockdown of RBPMS, RBFOX2, MBNL1 and MBNL2 on differentiated alternative splicing pattern of selected events were evaluated by RT-PCR following two siRNA treatments in PAC-1 cells (see Supplementary Figure S20C and D). Statistical analysis was performed with the PSI values (Supplementary Figure S20C). Unit variance scaling was applied to PSI values to standardize each row, resulting in a mean of 0 and SD of 1 in either direction (colour coded). Rows are centred and clustered using correlation distance and average linkage. Columns are clustered using maximum distance and average linkage. The knockdown was verified via RT-qPCR or western blots (Supplementary Figure S20A and B). Statistical analysis was performed using unpaired, one-tailed, Student’s *t*-test, indicated as follows: ns, *P* > 0.05; *, *P* < 0.05; **, *P* < 0.01; ***, *P*< 0.001; ****, *P*< 0.0001. For panels (D) and (E), signal-to-noise ratio between 25 and 75 nM is too low to conduct confidence comparison.

The preceding data indicate that RBPMS interacts with and actively recruits a number of RBPs in HeLa NE to TM3 RNA. In contrast to other RBPMS interactors, MBNL1 binds stably to short YGCY clusters upstream and downstream of *Tpm1* exon 3 independent of RBPMS (Figure 7E) and promotes exon skipping (37). To test whether MBNL1 modulates RBPMS activity on *Tpm1* splicing, we disrupted MBNL1 binding to the TM134 substrate either by deletion of both clusters or by mutation of all YGCYs to YCGY. These mutations led to reduced sensitivity to RBPMS regulation of splicing *in vitro*; higher concentrations of RBPMS (1.8–4-fold increase in SC_50_) were required to cause skipping of exon 3 in the MBNL site disrupted transcripts (Figure 8A and B; Supplementary Figure S21A and B). To complement these results, we titrated RNA oligonucleotides containing three YGCY or mutant YCGY motifs into *in vitro* splicing reactions. Addition of the MBNL-binding YGCY RNA oligonucleotide, but not the mutant YCGY RNA reversed the effect of sub-saturating RBPMS (70 nM) on *Tpm1* exon 3 skipping (Figure 8C; Supplementary Figure S21C), suggesting that MBNL1 acts in concert with RBPMS. The reduced activity of RBPMS upon MBNL site mutation was also reflected in reduced RBPMS cross-linking to TM3 RNA in NE (Figure 8D and E). RBPMS cross-linking to TM3 RNA is lower in the presence than absence of NE, presumably due to competition from other RBPs and splicing factors. We also noted differences in the absolute levels of RBPMS cross-linking to the different substrates. To assess the effects of MBNL binding site mutants, we therefore normalized RBPMS cross-linking in extract to cross-linking without extract, and refer to this as ‘NE-resistant cross-linking’ (Figure 8D and E). Upon mutation of the MBNL sites, NE-resistant cross-linking of RBPMS was significantly reduced by both MBNL binding site mutations (Figure 8D and E; Supplementary Figure S21D and E). Taken together, the preceding data suggest that protein–protein interactions with MBNL1 help to recruit RBPMS to TM3 RNA in NE, thereby explaining the inability of ΔC20 RBPMS to bind to or regulate TM134 RNA (Figure 2).

To examine the wider functional relevance of the identified RBPMS interactions, we tested the effects on four VSMC-specific ASEs (*Tpm1*, *Actn1*, *Flnb*, *Hspg2*) of siRNA-mediated knockdown in PAC-1 VSMCs of RBPMS, RBM4A and B, RBM14, RBM47, RBFOX2, MBNL1 and 2, and ESRP2. All the tested RBPs are expressed in PAC-1 cells, but only RBPMS shows significantly elevated expression in differentiated compared to proliferative cells (Supplementary Figure S17A). Depletion of targeted mRNAs and encoded proteins was verified by RT-qPCR and western blot (Supplementary Figures S17C and D, and S18–S20A and B). Only MBNL1/2 and RBFOX2 depletion had effects on all four ASEs in the same direction as RBPMS. We therefore expanded the ASE panel to include five RBPMS repressed (*Tpm1*, *Actn1*, *Itga7*, *Piezo1* and *Lsm14b*) and six RBPMS-activated (*Flnb*, *Hspg2*, *Ppfia1*, *Mycod*, *Ptprf* and *Ppfibp1*) splicing events (31). Strikingly, MBNL1/2 and RBFOX2 co-regulated all 11 ASEs in the same direction as RBPMS (Figure 8F; Supplementary Figure S20C), suggesting that they might work widely as RBPMS co-regulators of VSMC-specific ASEs. The only non-significant change was the effect of RBPMS on *Lsm14b*; however, this target was previously shown to respond to RBPMS knockdown in PAC-1 cells that were more differentiated than those used here (31). We noted that there were some indications of cross-regulation between RBPMS, MBNL1/2 and RBFOX2 (e.g. MBNL1/2 knockdown results in some depletion of RBPMS and RBFOX2; Supplementary Figure S20A and B). Nevertheless, this is not sufficient to undermine the conclusion that each protein directly affects splicing (see the next section), and further supports the functional integration of their splicing networks.

## DISCUSSION

**Figure 9.**
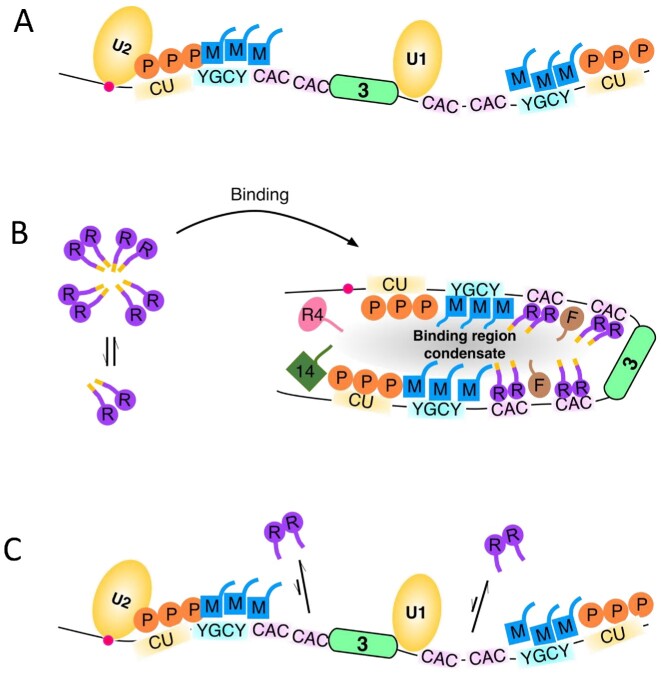
Summary model. RBP binding motifs, branch point (red dot) and *Tpm1* exon 3 are indicated in the strand of RNA (black line). snRNPs are depicted by yellow ovals. Coloured shapes are used to indicate various RBPs, PTBP1 (P), MBNL (M), RBPMS (R), RBFOX2 (F), RBM4 (R4) and RBM14 (14). (**A**) In the absence of RBPMS, co-repressors such as MBNL1 and PTBP1 can bind around *Tpm1* exon 3 but are not sufficient to prevent binding of U1 and U2 snRNPs. (**B**) RBPMS forms dynamic oligomers that can interact with other RBPs, including MBNL1 and RBFOX2, to block binding of U1 and U2 snRNPs. We propose that this assembly resembles a ‘binding region condensate’ as described by Hallegger *et al.* (29). (**C**) ΔC20 RBPMS fails to oligomerize, interact with other RBPs or bind to *Tpm1* RNA.

The activity of recombinant RBPMS *in vitro* allowed detailed analysis of the relationship between its biophysical properties and splicing regulatory activity and insights into how cell-specific splicing regulators interact physically and functionally with more widely expressed RBPs (Figure 9). *Tpm1* exon 3 is efficiently spliced in most cell types, and in HeLa NE *in vitro*, despite the binding of up to six PTBP1 and three to eight MBNL co-repressors around the exon (Figure 9A). RBPMS exists as a heterogeneous dynamic mixture of dimeric and oligomeric species (Figure 9B, left), with the C-terminal IDR mediating both homomeric oligomerization and heterotypic interactions with other proteins. Oligomeric RBPMS can therefore make multivalent interactions with the multiple (CAC)_2_ motifs flanking *Tpm1* exon 3 as well as contacting other RBPs, which might further stabilize RNA binding. Notably, MBNL1 binds independently to YGCY motifs and by a direct protein–protein interaction helps to recruit RBPMS to the RNA. RBPMS in turn recruits further co-regulators, including RBFOX2, that do not have specific binding sites. As a result, a stable repressed complex forms that prevents splicing complex assembly, including binding of U1 and U2 snRNPs (Figure 9B, right). With deletion of the C-terminal 20 amino acids of the IDR, RBPMS exists only as a dimer, is unable to interact with other RBPs and consequently is inactive as a splicing regulator, being unable to promote regulatory complex assembly (Figure 9C). We propose that the stable repressed complex, which encompasses a 500-nt region surrounding exon 3 (Figure 2D), resembles the ‘binding region condensates’ described by Hallegger *et al.* (29). Single-molecule analyses showed that the TM3 RNA binds ∼5–6 PTBP1 and 3–8 MBNL1 molecules (35,37). Equivalent analyses of RBPMS binding have not yet been carried out, but the size of RNA-free RBPMS oligomers (Figure 1) suggests that the repressed complex likely contains 5–10 RBPMS dimers, meaning that the size of the RBPMS:MBNL1:PTBP1 complex would be ∼1 MDa or larger, without taking into account RBFOX2 and other RBPs that do not have specific binding sites around exon 3. Despite this size, the repressive mode of action must be very precisely targeted because the 5′ss of exon 2, only 41 nt upstream of the exon 3 branch point, is activated by the repressive mechanism operating on exon 3, even on a *trans*-splicing substrate (Figure 4; Supplementary Figure S5). It seems plausible that the ‘zone of repression’ is limited by the two PTBP1 binding tracts, which flank the upstream and downstream MBNL and RBPMS binding sites (Figures 2D and 9).

Recombinant RBPMS primarily exists as a heterogeneous dynamic mixture of dimeric and oligomeric species, characteristic of phase-separating proteins below their critical saturation concentration (*c*_sat_) (60,61) (Figure 1D), although it can undergo phase separation forming liquid-like droplets *in vitro* (Figure 1J; Supplementary Figure S4B). While the reported association of RBPMS and RBPMS2 with cytoplasmic granules may involve condensate behaviour (42,46,62), we envisage that as a splicing regulator RBPMS is in the form of dynamic co-regulator-containing hetero-oligomers smaller than the mesoscale assemblies that form visible cellular condensates. The size of the transcript-bound RBPMS oliogomers could be addressed in the future using single-molecule methods (35,63). The disordered 20-amino acid C-terminal tail, enriched in aromatic and basic residues, is essential for RBPMS oligomerization (Figure 1), alternative splicing outcomes (Figures 1, 2 and 4), cooperative binding to multivalent RNA (Figures 2 and 3), splicing complex regulation (Figure 5) and most protein–protein interactions (Figure 6). These effects of the ΔC20 deletion are consistent with, and provide a physical basis for, previous reports that C-terminal truncation of RBPMS or RBPMS2 impaired localization to cytoplasmic granules (62), participation in RNP complexes (44), co-immunoprecipitation with its FL counterpart and mRNA binding (45).

The relative importance of the homomeric and heteromeric interactions mediated by the IDR remains an open question. Indeed, it is plausible that homotypic and heterotypic interactions share a common physical basis—for example, π–π or cation–π interactions mediated by aromatic and/or basic residues (64)—so mutants to distinguish their roles might be elusive. Nevertheless, given the multiple CAC motifs around *Tpm1* exon 3 the ability of RBPMS both to oligomerize and to mediate heterotypic interactions with other RBPs appears to be essential for its function. Indeed, the ability to interact with MBNL1 appears important for recruiting RBPMS to TM3 RNA in the face of competitive binding in NE (Figure 8). Modulation of RBPMS activity via deoligomerization also appears to be a physiological control mechanism. RBPMS is phosphorylated at Thr113/118 immediately downstream of the RRM, and phosphomimetic mutants have reduced activity and RNA binding, which is related, in part, to deoligomerization, as well as direct occlusion of the RNA binding surface of the RRM in a phosphomimetic mutant (65).

While many new RBPMS interactors were identified by affinity pull-down (Figure 6), 10 proteins in our dataset are known RBPMS interactors, including RBFOX2, MBNL1 and RBM14 (66–69). That most interactions are direct but enhanced by the presence of RNA (Figure 6B) is consistent with combinatorial models of splicing regulation, in which the low affinity of binary protein–protein interactions is tuned so as to enable specific cooperative assembly only upon regulated substrates with the correct combination of binding sites (70). Given the heterogeneity of RNAs in NE, the captured RBPMS interactome is likely to contain RBPMS co-regulators involved in both splicing activation and repression as well as other activities such as 3′-end processing (Figure 6; Supplementary Figure S15). Indeed, the identification of numerous U2 snRNP components (Figure 6A) suggests possible mechanisms for RBPMS splicing activation by U2 snRNP recruitment. However, it is less clear how this interaction could be involved in the observed displacement of U2 snRNP from *Tpm1* transcripts (Figure 5; Supplementary Figures S10 and S11), which is more likely explained by the earlier displacement of U2AF2 by RBPMS (Figure 7F). In examining how RBPMS remodels the Tpm1 RNA-bound proteome, we would ideally have used similar conditions to those used to identify ATP-dependent complexes on native gels (Figure 5D). However, we encountered insurmountable technical problems in trying to purify complexes assembled in the presence of PVA and with the longer (820 nt) TM23 substrate. In the future, it would be useful to exploit single-molecule methods to assess how RBPMS affects binding of individual snRNPs to the TM RNAs as well as the number of RBPMS subunits associated with the repressed complex (35,63). Nevertheless, by analysing complexes formed on TM3 RNA in the absence of PVA, we identified potential splicing co-regulators of RBPMS (Figure 7), many of which were also pulled down directly by RBPMS from HeLa NE. In contrast, several known splicing regulators detected in the AP–MS experiment were either depleted from TM3 by RBPMS (e.g. SRSF7 and hnRNPC) or not significantly enriched (e.g. SRSF1). Some of these differences could be attributed to the presence or absence of specific *cis*-elements in the TM3 substrate, which may be a requirement for recruitment of some interactors, such as RBM4 whose interaction was completely RNA dependent (Figure 6B). RBM4 was previously reported to promote Tpm1 exon 3 inclusion, antagonizing the activity of PTBP1 (71), but we saw no effects upon RBM4 knockdown (Supplementary Figure S17D).

Among RBPMS interactors, we identified many components of the 55S Benzonase-resistant LASR splicing regulatory complex (24) (Figure 6A). LASR confers the RNA binding specificities of its other constituent proteins upon RBFOX, effectively expanding the motif recognition preference of RBFOX, consistent with the lack of identifiable RBFOX motifs associated with many RBFOX CLIP tags (72). Nevertheless, the higher-order Benzonase-resistant RBPMS interaction with RBFOX2 (Figure 6C) did not involve other LASR complex components (e.g. MATR3 or hnRNP M), so it may be a distinct complex. RBFOX2 has been shown to direct distinct splicing outcomes of opposing biological activities by partnering with different splicing regulators (73). RBPMS may have such a determining influence, redirecting RBFOX2 from promoting mesenchymal (74) to differentiated VSMC splicing programmes. One interesting example is the *Flnb* H1 exon, encoding a hinge region in filamin B, which is activated in PAC-1 cells by RBPMS, RBFOX2 and MBNL1 (Figures 8; Supplementary Figures S18–S20) (31). However, in human breast cancer cells RBFOX1 promoted skipping of the same exon, as part of epithelial–mesenchymal transition (75).

Several lines of evidence converge to suggest that MBNL1 and RBFOX2 act as general co-regulators with RBPMS. Both proteins were found as direct RNA-stimulated interactors with RBPMS (Figure 6): RBFOX2 was recruited to the TM3 RNA by RBPMS (Figure 7), while MBNL1 bound TM3 independently via its own binding sites and helped to recruit RBPMS in NE (Figure 8). Knockdown of both proteins affected 11 tested ASEs in the same direction as RBPMS (Figure 8; Supplementary Figure S20C). We noted that there was evidence of cross-regulation between RBPMS, RBFOX2 and MBNL1, particularly at the protein level (Supplementary Figure S20). Despite this, the data support direct roles of all three proteins in the ASEs tested. First, RBPMS depletion did not affect RBFOX2 and actually led to small apparent increases in MBNL levels, which would act to dampen RBPMS affects, so we can conclude that RBPMS effects are explained by its own knockdown. Second, RBFOX2 knockdown led to partial RBPMS and MBNL1 protein depletion (Supplementary Figures S18–S20). However, RBFOX2 knockdown had a larger effect than RBPMS knockdown on many events, arguing that it acts directly as well as by reducing RBPMS levels. Finally, although MBNL1/2 knockdown also partially reduced levels of RBFOX2 and RBPMS, it also had the greatest effect on all but one ASE (Itga7; Supplementary Figure S20C), again arguing that it acts directly. We do not know the molecular basis of most of the cross-regulatory effects. However, RBPMS (31) causes an exon skipping in MBNL1 and 2, which has been shown to mediate post-translational downregulation of MBNL2 protein (76), which might explain the MBNL protein upregulation upon RBPMS knockdown.

Other lines of evidence also suggest widespread functional cooperation of RBPMS and RBFOX proteins, including enrichment around RBPMS-regulated exons not only of RBPMS dual CAC binding motifs but also of RBFOX binding GCAUG motifs (77), the identification of a GCAUG-containing motif as the top intronic binding site for RBPMS in ES cells (78) and the association of both RBFOX and RBPMS binding motifs with ERG (E26-related gene protein)-repressed exons in HeLa cells (79). Furthermore, the SMC transcription factor MYOCD was recently found to indirectly drive a set of SMC AS changes via changes in the expression levels of RBPMS, RBFOX2 and MBNL1 (80), and both *Rbpms* and *Mbnl1* were found by single-cell RNA sequencing to be part of a contractile VSMC gene signature (81). Indeed, it has been suggested that gastrointestinal dysfunction in myotonic dystrophy is associated with dysregulation of an MBNL1-regulated splicing programme in visceral smooth muscle cells (82). Our data suggest that this dysregulated programme is likely driven by MBNL1–RBPMS co-regulation. The finding that recombinant RBPMS is sufficient *in vitro* to switch *Tpm1* exon 3 alternative splicing to the fully differentiated VSMC state (Figure 2) is consistent with its proposed role as a master regulator of the AS splicing programme in differentiated VSMCs (31). *Rbpms* heterozygous knockout mice have no phenotype, while homozygous knockouts are inviable (83) but have phenotypes associated with dysfunction of both VSMCs and cardiomyocytes. Confirmation of the physiological roles of RBPMS in fully differentiated VSMCs *in vivo* will therefore require conditional knockout models.

In conclusion, this study builds on previous work to suggest that the dynamic regulation of splice site choice is dependent on the existence of both constitutive and tissue-specific AS regulatory networks. The intricate connections and functional redundancy of the two networks may reflect the requirement of VSMCs to conduct phenotypic switching rapidly in response to environmental cues.

## Supplementary Material

gkad652_Supplemental_Files

## Data Availability

The proteomes recovered from RNA-assisted pull-down and Strep-tagged RBPMS affinity pull-down were analysed using label-free quantitative LC–MS/MS. The raw mass spectrometry data were submitted to the ProteomeXchange Consortium via the PRIDE partner repository (84) and are identified by dataset numbers PXD037617 and PXD037620, respectively.
