# Supplementary material for: Cell-type specific regulator RBPMS switches alternative splicing via higher-order oligomerization and heterotypic interactions with other splicing regulators
Source: Nucleic Acids Res. 2023 Aug 7;51(18):9961–82. doi: 10.1093/nar/gkad652 (PMC10570038; doi:10.1093/nar/gkad652)
Supplement: gkad652_Supplemental_Files [file gkad652_supplemental_files.zip › Supplementary Figures and Tables.pdf]

Cell-type specific regulator RBPMS switches alternative splicing *via* higher-order oligomerization and heterotypic interactions with other splicing regulators

Yi Yang, Giselle C Lee, Erick Nakagaki-Silva, Yuling Huang, Matthew Peacey, Ruth Partridge, Clare Gooding, Christopher WJ Smith

Department of Biochemistry, University of Cambridge, CB2 1QW, Cambridge, UK

Supplementary Figures S1-21

Supplementary Tables S1-11

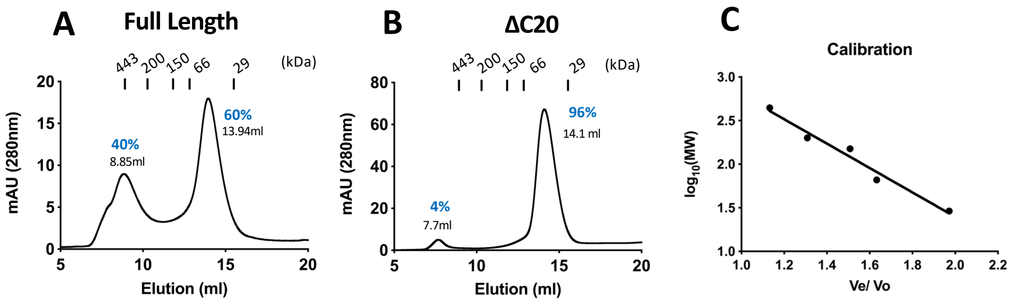

D

His$_6$-RBPMS	Elution volume (V_e), ml	Construct Monomeric MW, kDa	Apparent MW, kDa	Void volume(V_O), ml
Full-length (FL)	13.94	23.93	52.12	7.87
ΔC20	14.1	21.55	48.75	7.87

Figure S1. Size exclusion chromatography analysis of RBPMS constructs on Superdex 200 100/300 GL column. (A) Elution of full-length His$_6$ tagged RBPMS. (B) Elution of His$_6$ tagged or ΔC20 RBPMS. (C) Calibration curve of the Superdex 200 column. Calibration was carried out using purified proteins containing: Apoferrin from horse spleen, β-Amylase from sweet potato, Alcohol Dehydrogenase from yeast, Bovine Albumin, and Carbonic Anhydrase from bovine erythrocytes, the molecular weights are 443, 200, 150, 66 and 29 kDa, respectively. The void volume was determined separately using Blue Dextran. (D)Table of apparent molecular weight determination.

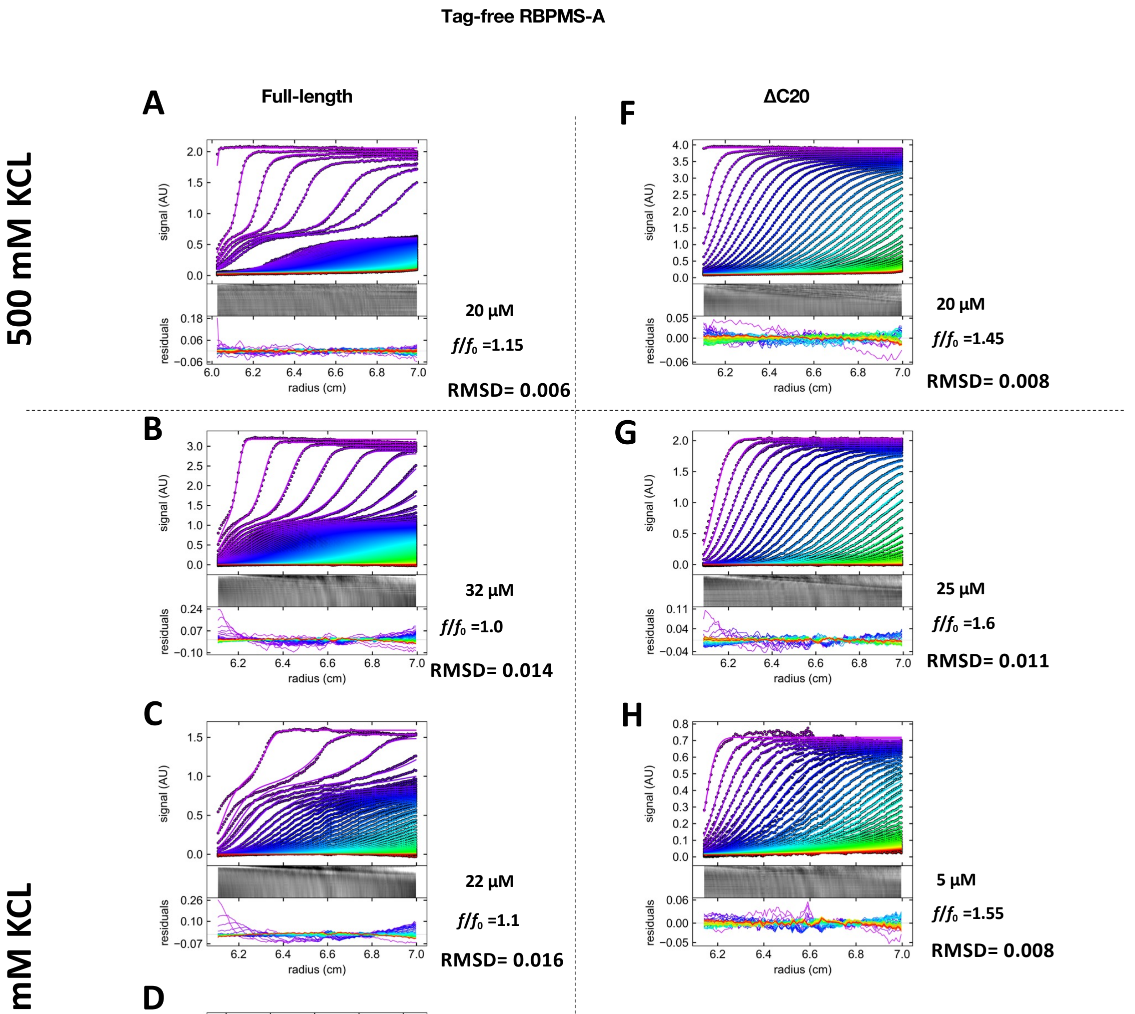

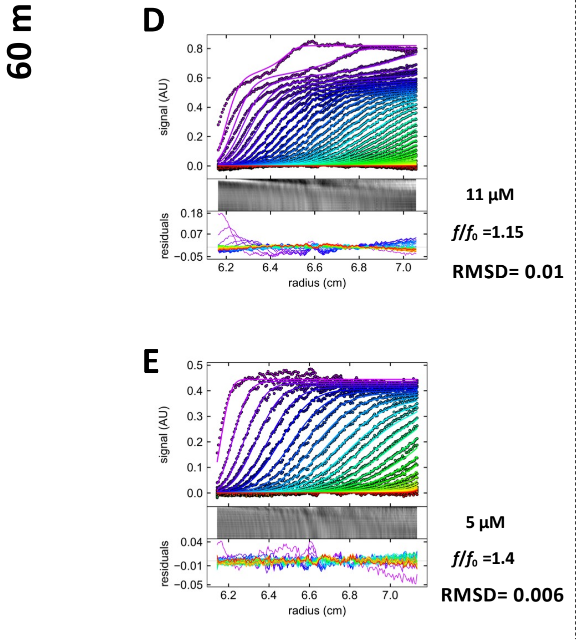

Figure S2. Fitting of the sedimentation boundaries of RBPMS in 500 mM or 60 mM KCl using continuous *c(s)* model. Tag-free RBPMS constructs used are indicated on top of the panel. The protein concentration in μM is denoted in each panel. Every tenth scan boundary was shown. The sedimenting boundaries over time were colored coded using purple to blue to green transition.

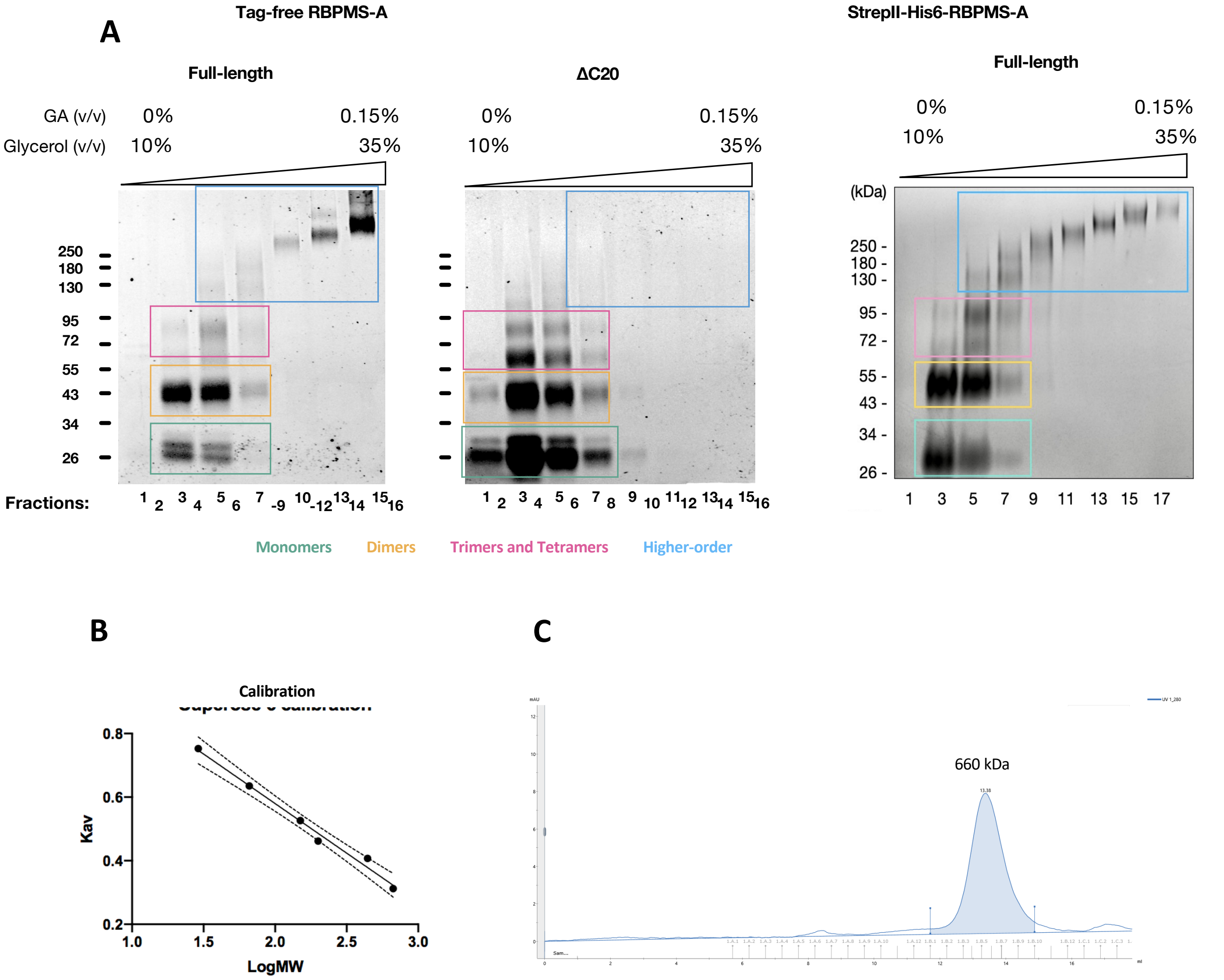

Figure S3. Top panel: RBPMS oligomerization was captured by GraFix glycerol gradients. (A) Glycerol gradient separated of glutaraldehyde (GA) crosslinked RBPMS constructs were fractionated, analysed on a 3-12% SDS-PAGE gel, and visualized by silver staining. Monomers, dimers, trimers and tetramers, and higher order oligomers were highlighted by colored boxes. Asterisk indicates fractions selected for further purification described below. Bottom panel: Size exclusion chromatography analysis of RBPMS constructs on Superose 6 increase column. (B) Calibration curve of the Superose 6 increase column. Calibration was carried out using purified proteins containing: Bovine Thyroglobin, Apoferrin from horse spleen, β-Amylase from sweet potato, Alcohol Dehydrogenase from yeast, Bovine Albumin, and Carbonic Anhydrase from bovine erythrocytes, the molecular weights are 669, 443, 200, 150, 66 and 29 kDa, respectively. The void volume was determined separately using Blue Dextran. (C) Elution of RBPMS high order oligomer. The apparent molecular weight of the peak was indicated on the top. Fractions shadowed by blue were concentrated for Cryo-EM analysis

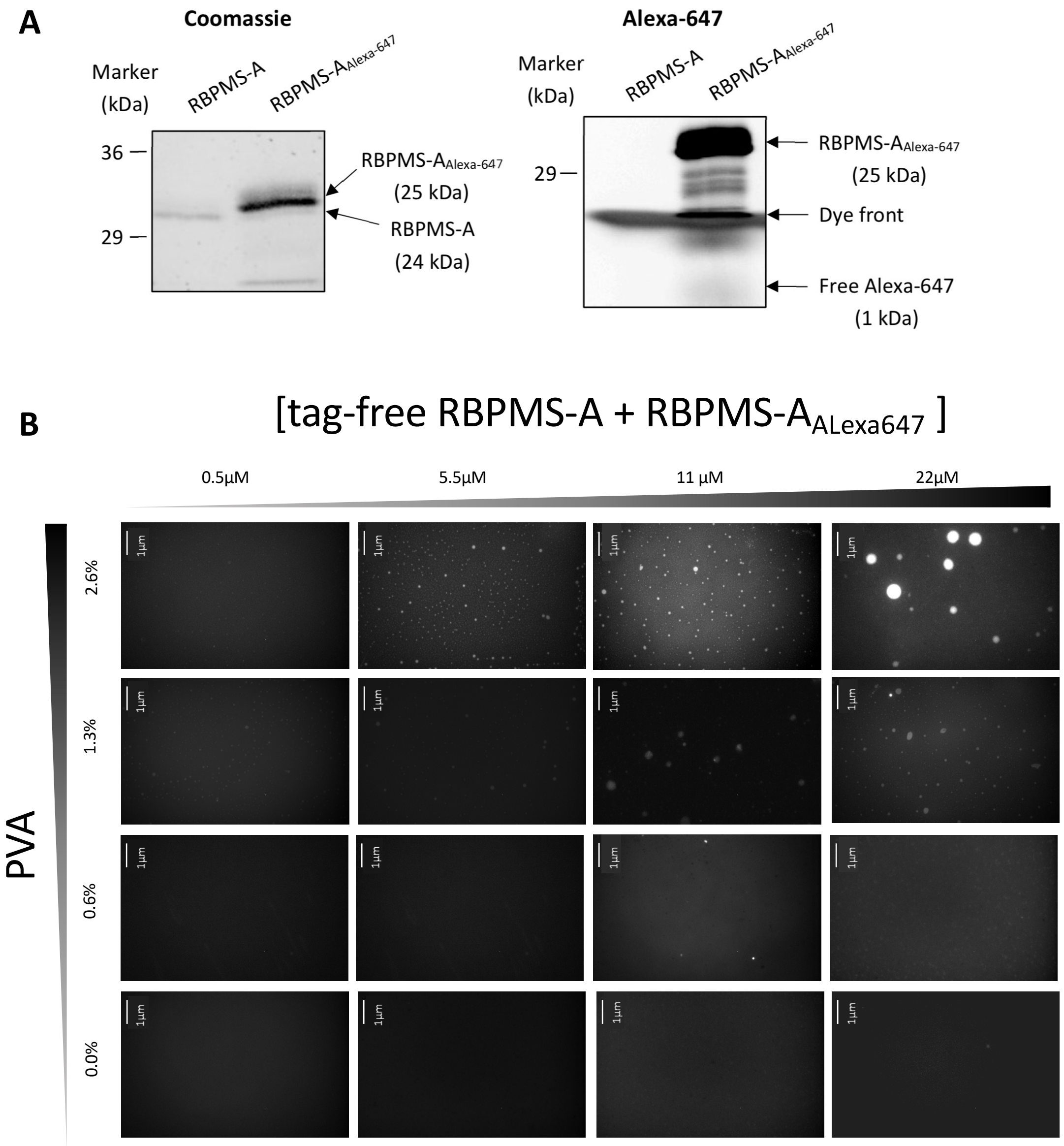

Figure S4. Fluorescent labeled of His_6-TEV-RBPMS-A . (A) SDS-PAGE analysis of recombinant RBPMS-A before and after labeling. Left and right images are of the same gel, showing either Coomassie stained or Alexa-647 fluorescent labeled protein. (B) Fluorescent images of tag-free RBPMS-A LLPS droplets at 90 mM KCL and various PVA concentration. Fluorophore-conjugated His_6-TEV-RBPMS-$A_{Alexa\text{-}647}$ is added to 0.5 µM to give the final concentration shown. $\%_{v/v}$ of PVA was added to examine the RBPMS LLPS propensity.

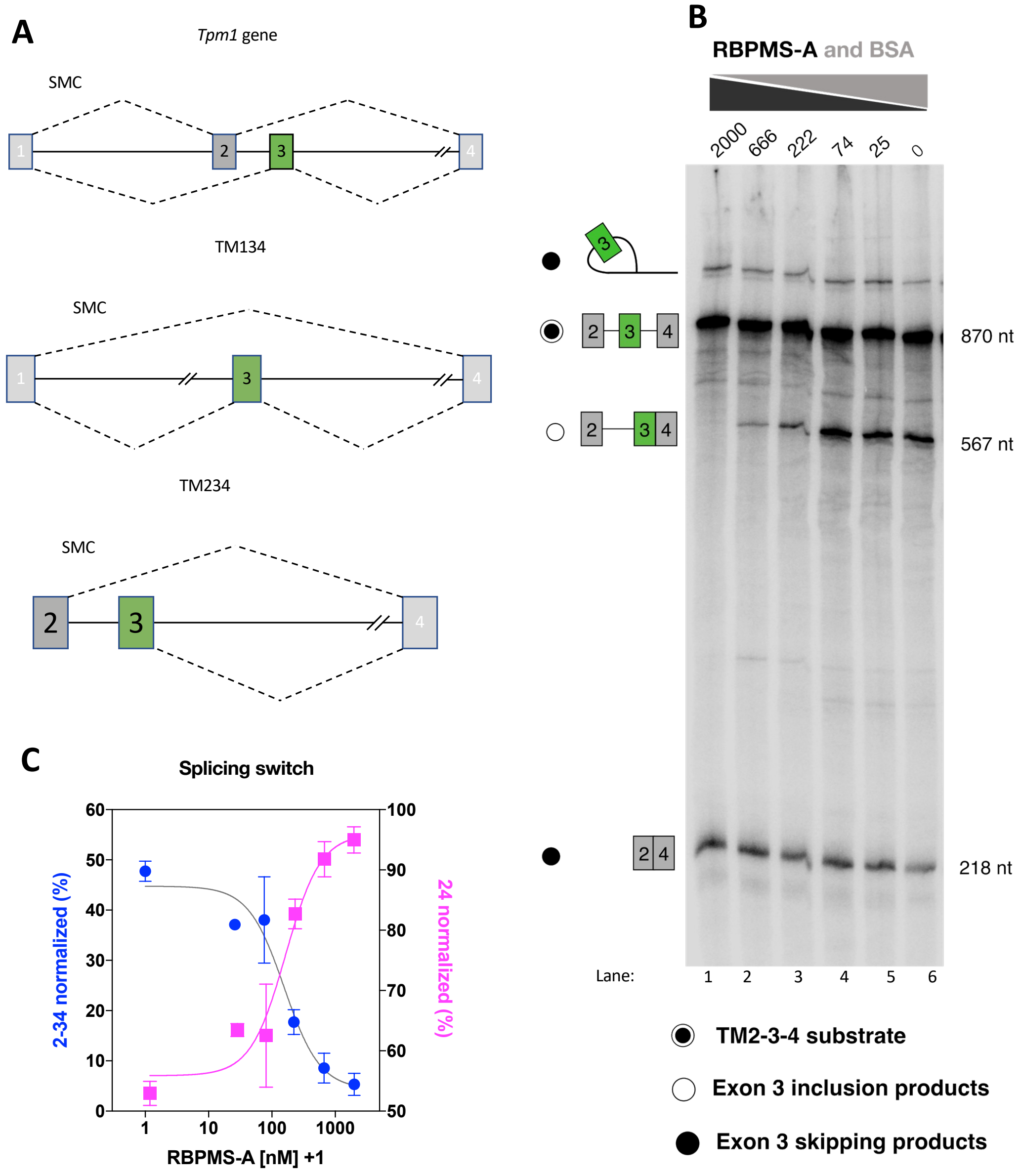

Figure S5. Concentration-dependent RBPMS-A modulation of TM234 *in vitro* splicing reaction. (A) Schematic of genomic organization of *Tpm1* exons 1-4, and the derived TM134 and TM234 experimental transcripts used here. (B) Titration of full-length RBPMS-A into the *in vitro* TM234 splicing reaction. The identities of the linear spliced products are inferred by nucleotide length and depicted by schematic diagrams. The identities of the lariat are determined by matching against the previous *in vitro* splicing experiments [Gooding 1994] of the same substrate. The pre-mRNA substrate TM234 is indicated by the concentric circle. 2-34 splicing products are indicated by an opened circle, while filled circles are placed aside of the 2-4 splicing products. One representative of three technical repeats is shown. (C) Size normalized quantification of switching between 2-34 (left y-axis, blue) and 2-4 splicing products (right y-axis, pink). Mean and standard deviation of three technical repeats was plotted. Nonlinear regression was performed with log (inhibitor) vs response-variable slope equation provided by GraphPad Prism 9.

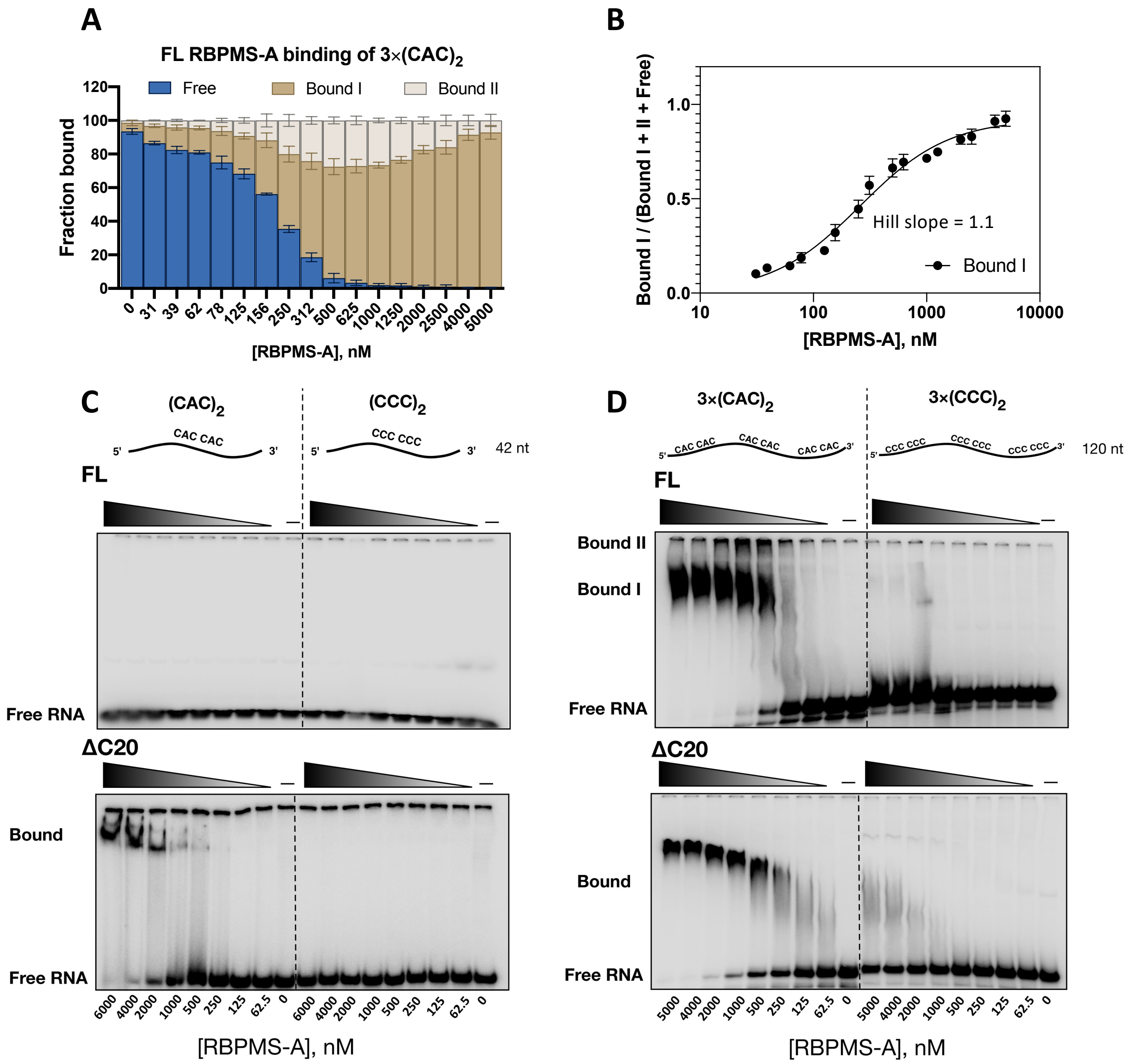

Figure S6. (A) Quantification of the two bound species of FL RBPMS-A binding to 10 nM 3×$(CAC)_2$. Image shown in Fig. 3B. Mean and standard deviation of three technical repeats was plotted. (B) The specific binding of Bound I was determined as Bound I/(Bound I + II +Free), which was plotted against protein concentration. Mean and standard deviation of three technical repeats was plotted. Data points of protein at 0 nM, were omitted due to the log scaled x-axis. The curves were fitted using specific binding with the Hill slope equation (see M&M) provided by GraphPad Prism 9 package. FL and ΔC20 RBPMS-A binding to 10 nM $(CAC)_2$ or $(CCC)_2$ substrates in electrophoretic mobility shift assay (EMSA). (C) [32p-CTP] incorporated synthetic RNA of 42 nt, containing either single tandem CAC or CCC motif (10 nt spacer Fig 2D), as illustrated on top, was incubated with 0-6 µM RBPMS recombinant protein as indicated and resolved on a 5% native polyacrylamide gel. (D) [^{32}P-CTP] incorporated synthetic RNA of 120 nt, containing either three tandem CAC or CCC motifs (10-16 nt spacers Fig 2D), as illustrated on top, was incubated with 0-5 µM RBPMS recombinant protein as indicated and resolved on a 5% native polyacrylamide gel. The binding reactions carried out with either FL or ΔC20 RBPMS-A were indicated on top left corner of each gel.

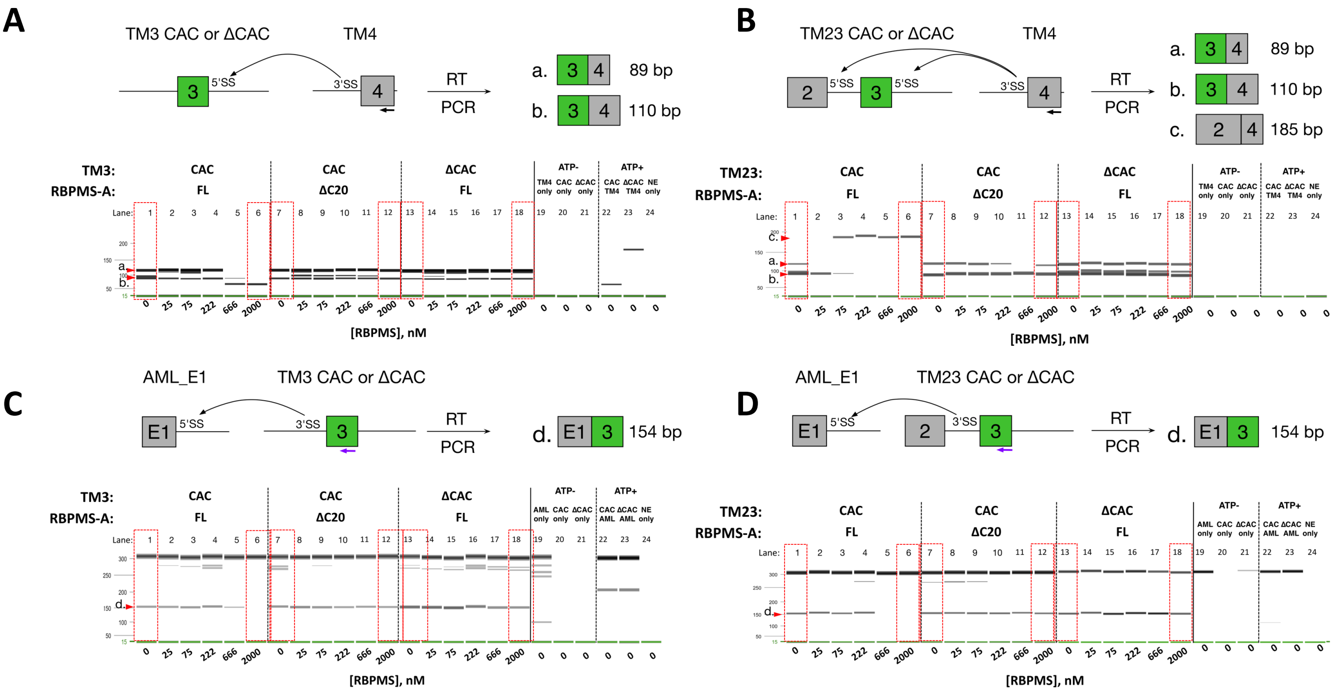

Figure S7. RBPMS regulates 5' and 3' SS on TM3 and TM23. The top panels illustrate schematic diagrams showing the *trans*-splicing reactions (black curved arrows) between the TM3 (A) or TM23 (B) and the 3'SS of the TM4 *trans* partner. Sensitivity to triply diluted series of FL or ΔC20 RBPMS was tested on either CAC sufficient (CAC) or deficient (ΔCAC) exon 3 containing substrate. After incubation with NE, total RNA was recovered and reverse transcribed (RT) with primer indicated (black). In (A), PCR reactions aimed at amplifying 3-4 *trans* spliced product. QIAxel digital PCR product *a* (red arrow), 89 bp, is the intended 3-4 PCR product. Band *b* (red arrow), 110 bp, was the result of mispriming of the reverse primer at +20 position with respect to the on-target priming. However, both products report 3-4 *trans* splicing product faithfully. In (B), An three-primers PCR reaction detecting both 2-4 or 3-4 *trans* splicing products was used. The identical PCR products *a* and *b* (red arrow), indicative of 3-4 spliced products were detected, while band *c* appears under FL RBPMS-A modulation (> 75 nM, lane 3). Lower panels illustrate the *trans*-splicing reactions (black curved arrows) between the TM3 (C) or TM23 (D) and the 5'SS of the AML exon 1. Sensitivity to *cis*- and *trans*-elements was tested as described above. RT was performed with a primer complementary to the 3' end of exon 3 (purple). In (C-D) The identical PCR product, *d,* indictive of AML_E1-3 *trans* spliced products were detected, but subjected to FL RBPMS-A repression. For all RT-PCR panels, negative controls were performed including, splicing of the trans-partner alone (Lane 19) , splicing of CAC or ΔCAC exon 3 substrate (Lane 20-21), ATP depleted NE incubation of CAC or ΔCAC substrate in combination with trans partner (Lane 22-23), and NE alone (Lane 24). Lanes used in Fig 4 were highlighted with a red dashed box.

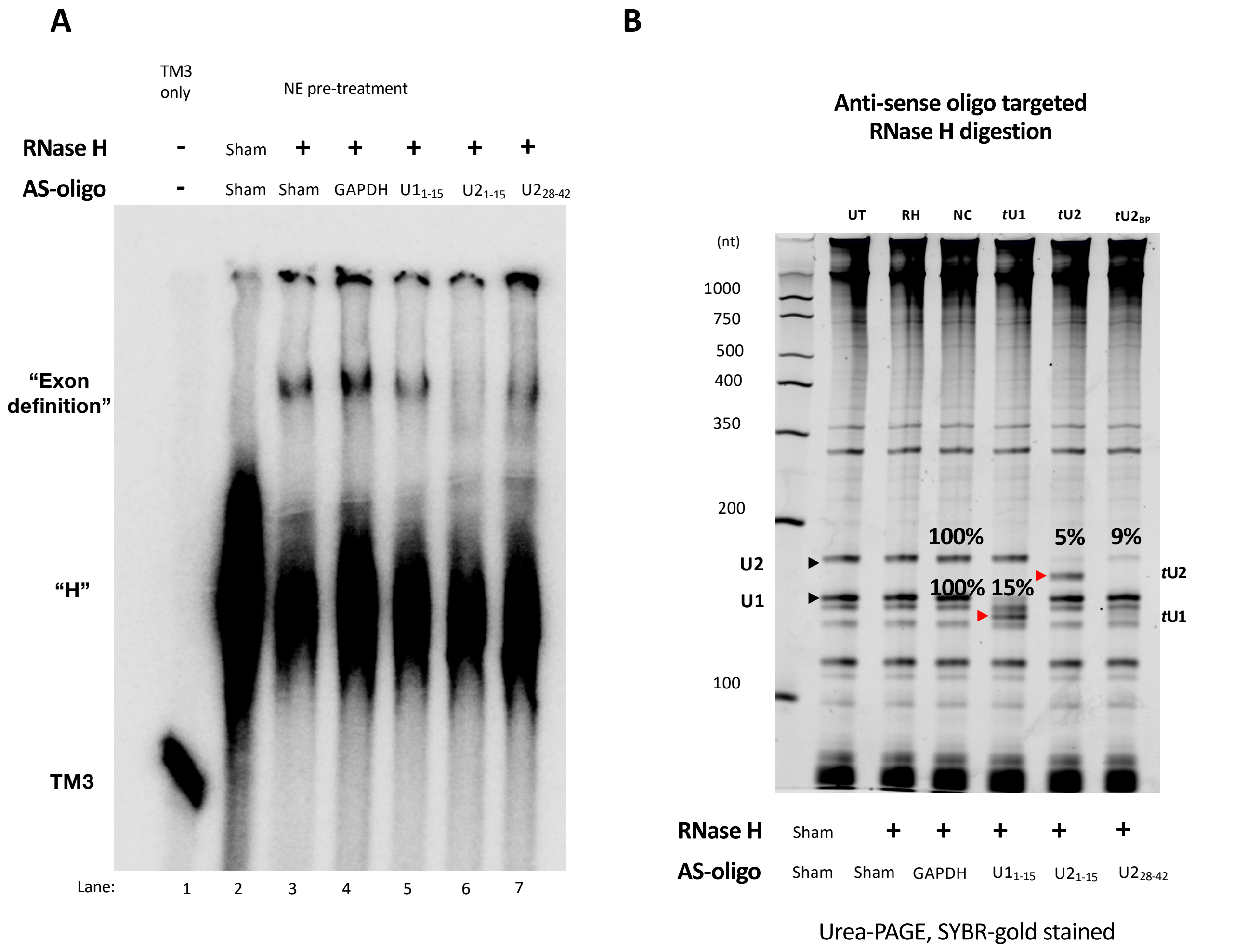

Figure S8. Pre-spliceosomal complex progression is dependent on U1 and U2 snRNP. (A) Miniaturized Tpm1 exon 3 model RNA, TM3, was incubated in untreated nuclear extract (NE), sham-treated NE, or NE pre-treated with RNase H in combination with DNA oligonucleotide targeting GAPDH mRNA, $U1_{1\text{-}15}$, $U2_{1\text{-}15}$, and $U2_{28\text{-}42}$. Lane 1, free TM3 RNA. (B) SYBR Gold stained urea-PAGE evaluation of the total NE RNA composition. The level of snRNA degradation was indicated and calculated by comparing it to the level of that the sham-treated condition. U1 or U2 snRNA mobilities before and after RNase H digestion were indicated by black or red triangle, respectively.

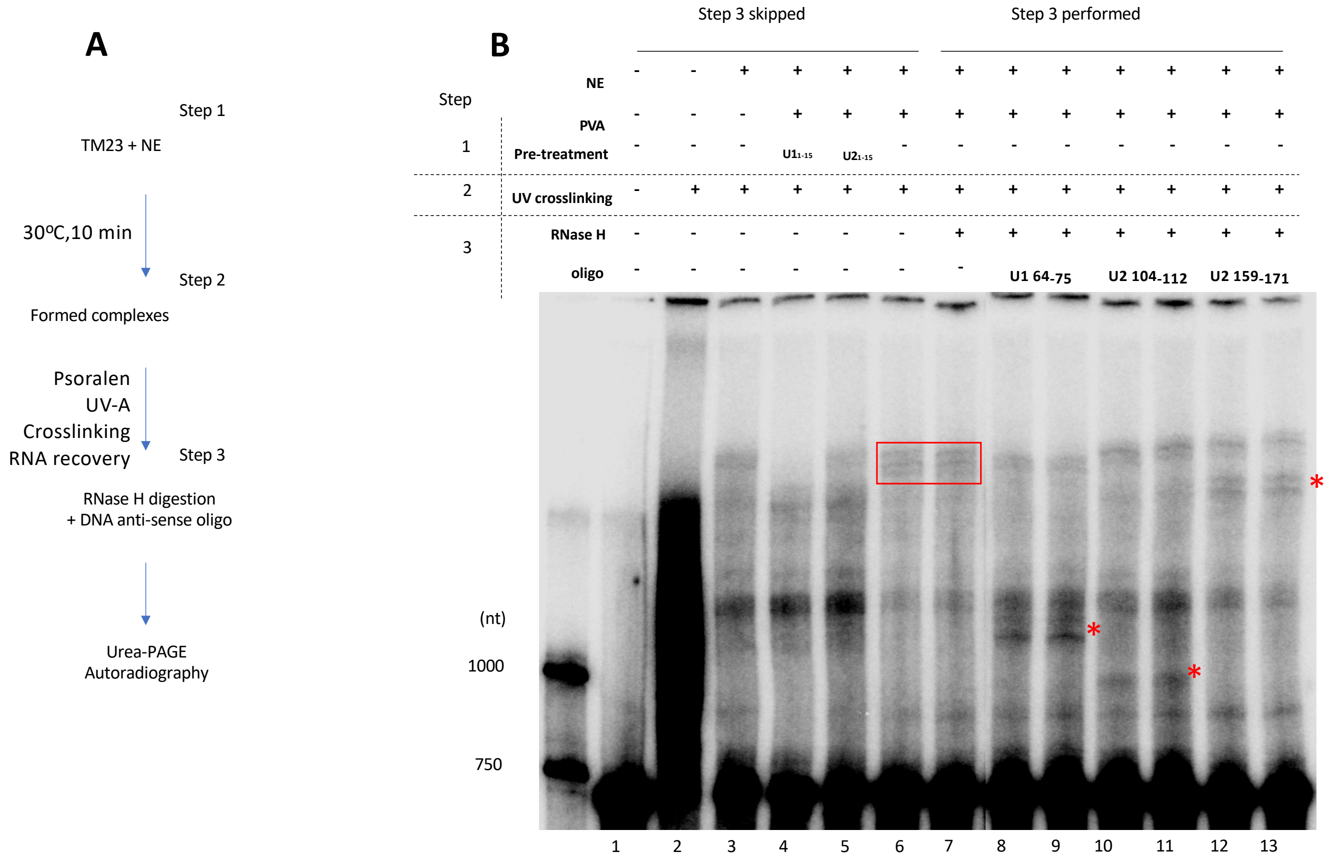

Figure S9. Anti-sense DNA oligonucleotide digestion of TM23-snRNA Psoralen crosslinks. (A) Flow chart summarizing the "three steps" sample preparation process. (B) Radiolabeled RNA species were resolved on a 4% Urea-PAGE TBE gel. Lane 1, TM23 RNA. Lane 2, crosslinking of incubated TM23 without NE. Lane 3, crosslinking of NE incubated TM23, but without PVA. Under such conditions, Pre-spliceosomal progression is discouraged. Lane 4-5, crosslinking of TM23 incubated in NE pre-treated with either $U1_{1-15}$ or $U2_{1-15}$ DNA anti-sense oligo. The complexes assembled were expected to be identical to that shown in Fig.4.B. Lanes 4-5. Lane 6-7, crosslinking of TM23 incubated in sham pre-treated NE. Lane 8-9, RNase H digestion of TM23-snRNA crosslink with $U1_{64-75}$ complementary DNA oligo. Lane 10-11, RNase H digestion of TM23-snRNA crosslink with $U2_{104-112}$ complementary DNA oligo. Lane 12-13, RNase H digestion of TM23-snRNA crosslink with $U2_{159-171}$ complementary DNA oligo. The triplet bands likely represent TM23-snRNA crosslinks are highlighted in the red box. The top band is responsive to U1 complementary DNA oligo digestion. The bottom two bands show sensitivity to U2 complementary oligo-targeted digestion. The digestion products were indicated with a red asterisk.

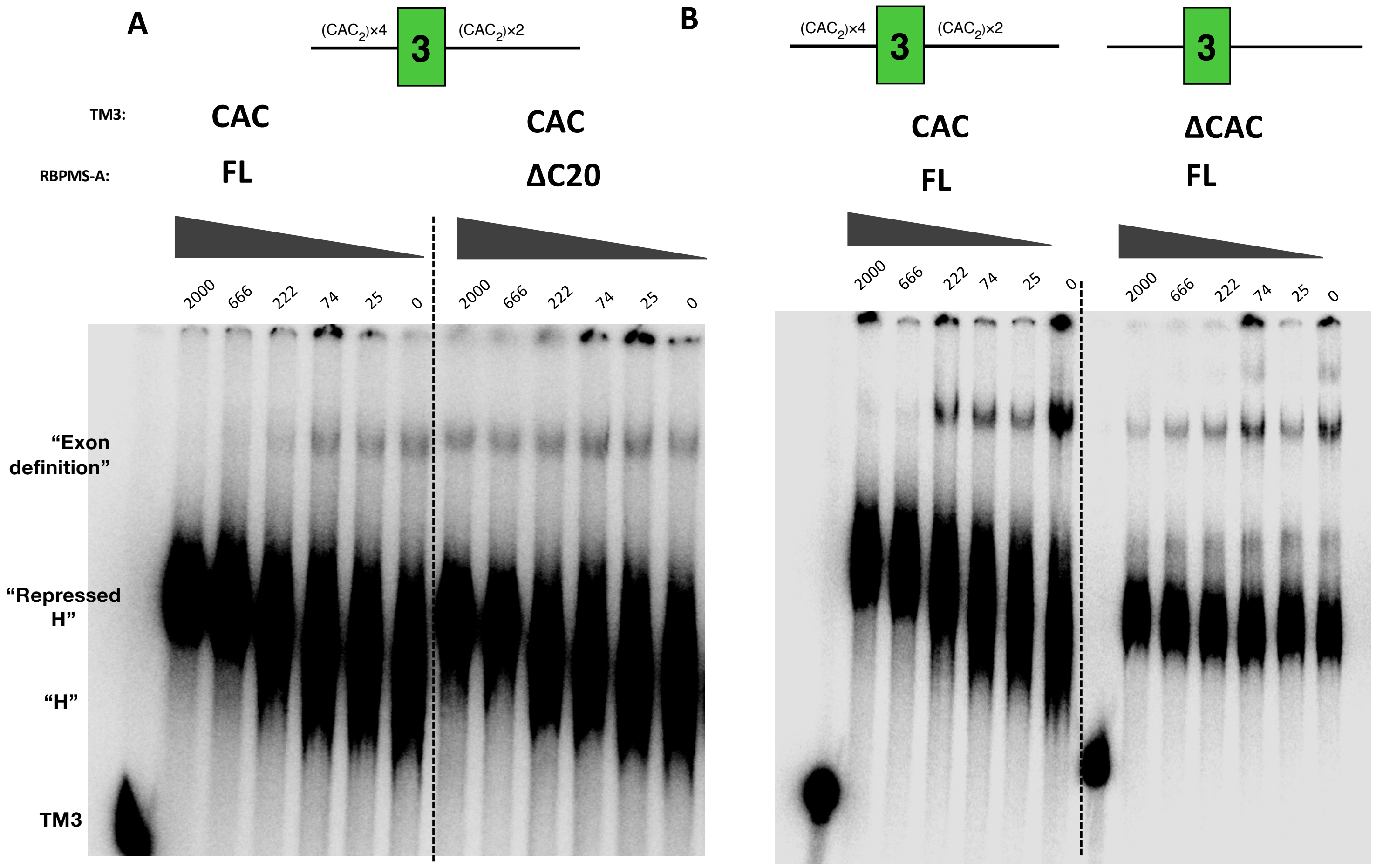

Figure S10. EMSA after incubation of TM3 (CAC) or CAC cluster deficient TM3 (ΔCAC) in NE in combination with FL or ΔC20 RBPMS-A.

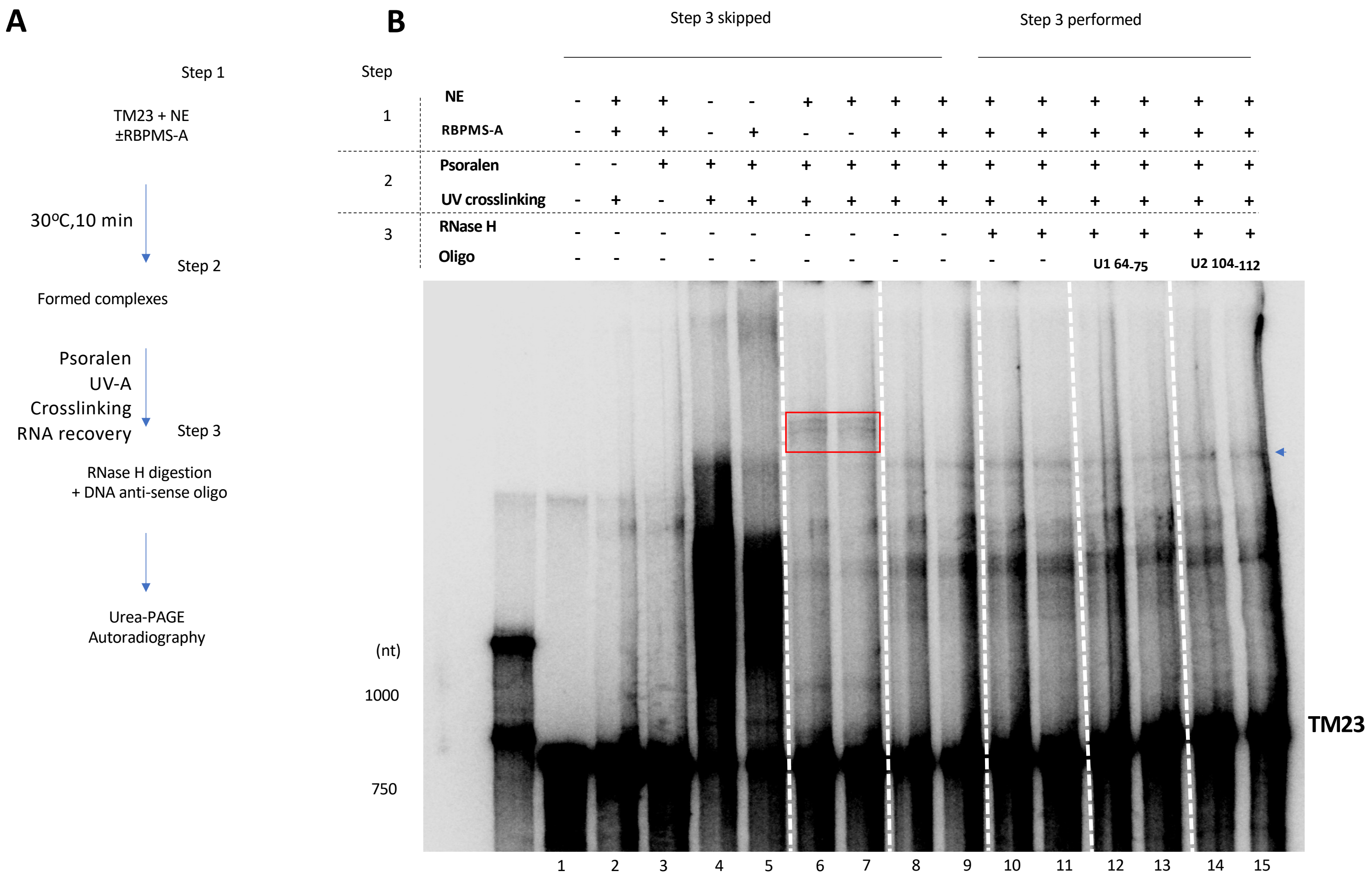

Figure S11. RBPMS inhibits U1 and U2 snRNP recruitment. (A) Flow chart summarizing the "three steps" sample preparation process. (B) Radiolabeled RNA species were resolved on a 4% Urea-PAGE TBE gel. Lane 1, TM23 RNA. Lane 2, RBPMS repressed TM23 complex radiated with UV-A. Lane 3, RBPMS repressed TM23 complex incubated with Psoralen without UV radiation. Lane 4, crosslinking of incubated TM23 without NE. Lane 5,crosslinking of incubated TM23 without NE but with RBPMS-A. Lane 6-7, crosslinking of TM23 incubated in NE. Lane 8-9, crosslinking of TM23 incubated in NE+ RBPMS-A. Lanes 10-11, the identically prepared RNA products shown in lanes 8-9 were sham-treated with RNase H. Lanes 12-15, the identically prepared RNA products shown in lanes 8-9 were treated with either $U1_{64-75}$ or $U2_{104-112}$ complementary DNA oligo in combination with RNase H. The triplet bands likely represent TM23-snRNA crosslinks are highlighted in the red box. A potential intramolecular TM23 crosslink is indicated by a blue arrow.

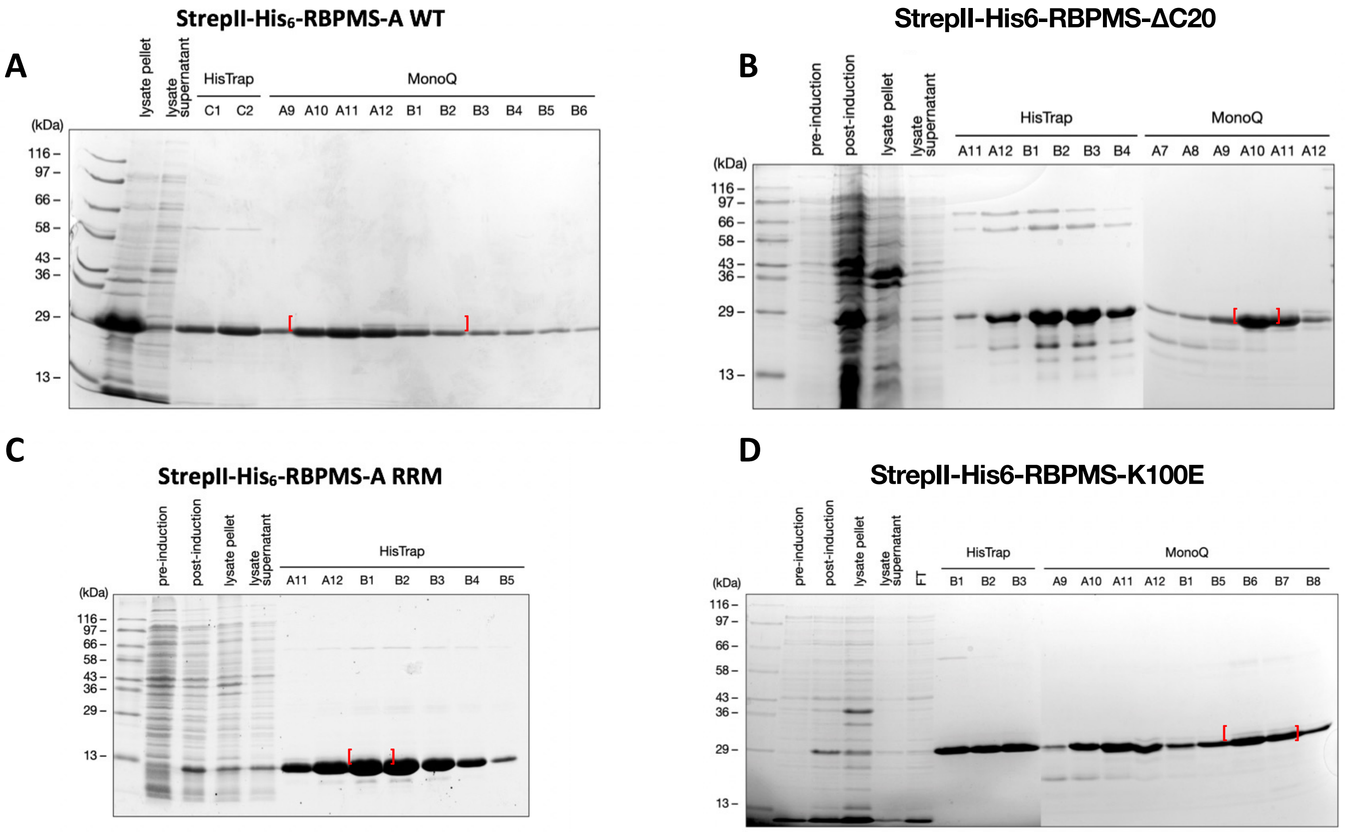

Figure S12. Recombinant Strep-II and His6 tagged RBPMS constructs used in the AP-MS and pull-down study. Selected fractions are bracketed.

A

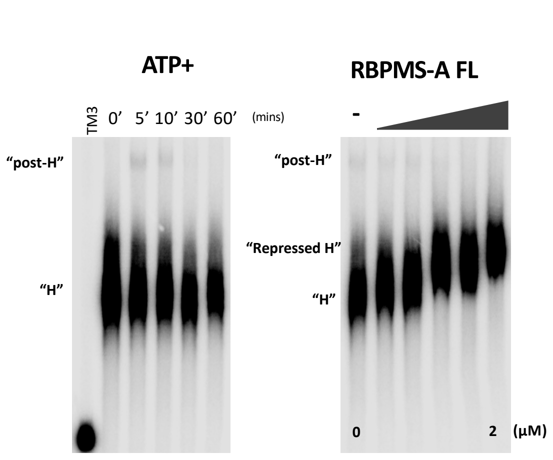

B

Group name	Condition	Samples in triplicate
G1	ATP- T- R-	N1, N3, N5
G2	ATP- T- R+	N2, N4, N6
G3	ATP+ T- R-	NA1, NA3, NA5
G4	ATP+ T- R+	NA2, NA4, NA6
G5	ATP- T+ R-	B1, B3, B5
G6	ATP- T+ R+	R1, R3, R5
G7	ATP+ T+ R-	B2, B4, B6
G8	ATP+ T+ R+	R2, R4, R6

Sample grouping

Background subtraction

(G5-G1)
(G6-G2)
(G7-G3)
(G8-G4)

Differential pulldown analysis

(G6-G2) vs (G5-G1) ATP- , RBPMS dependent

(G8-G4) vs (G7-G3) ATP+ , RBPMS dependent

C

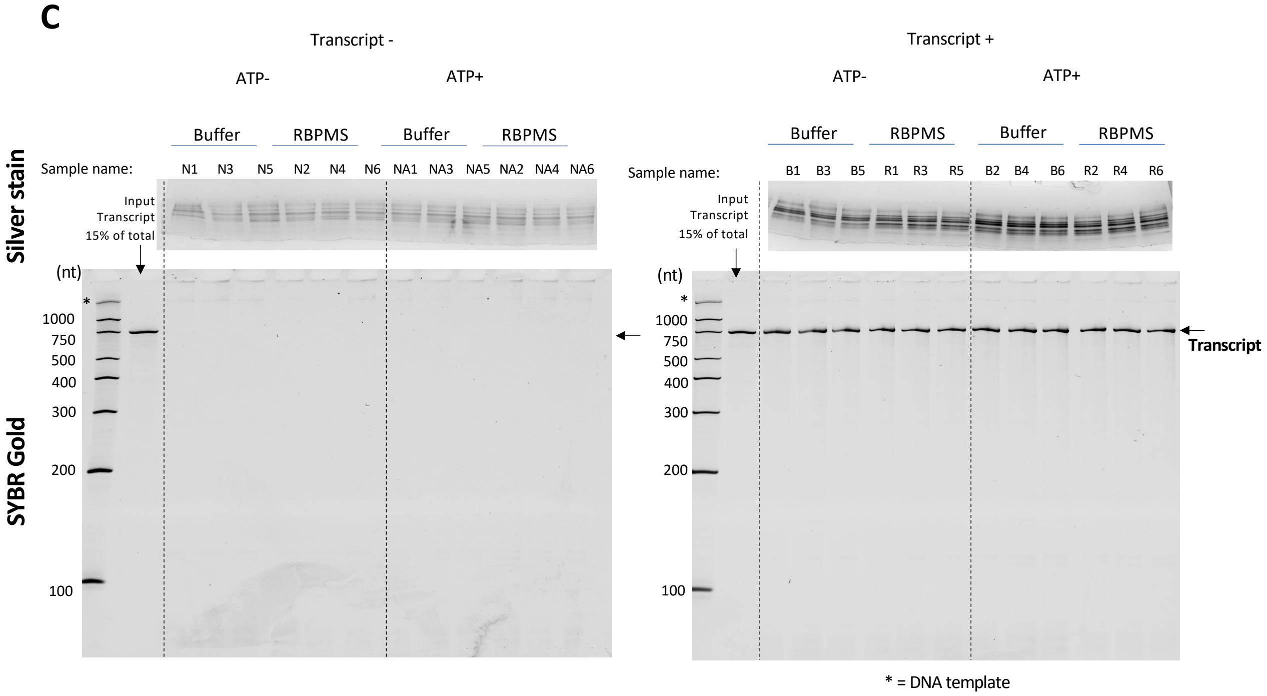

Figure S13. Sample preparation for RNA-assisted pull-down. (A) Native gel screening for complexes assembly condition on TM3 transcript without PVA. As specified in materials and methods, 1.5 µM RBPMS-A and 20 min assembly time were chosen for RNA affinity purification. (B) Flow chart summarizing the grouping, background subtraction, and differential pulldown analysis strategies. The top table detailed the experimental design. Each group contains three technical repeats under the condition defined by three variables: ATP, transcript (T), and RBPMS-A (R). (C) Top panel, silver-stained SDS-PAGE gel slides submitted to proteomic analysis. Bottom panel, the RNA compositions were demonstrated using SYBR gold stained Urea-PAGE gel and the percentage of recovery was estimated by comparing to 15% of the total RNA input.

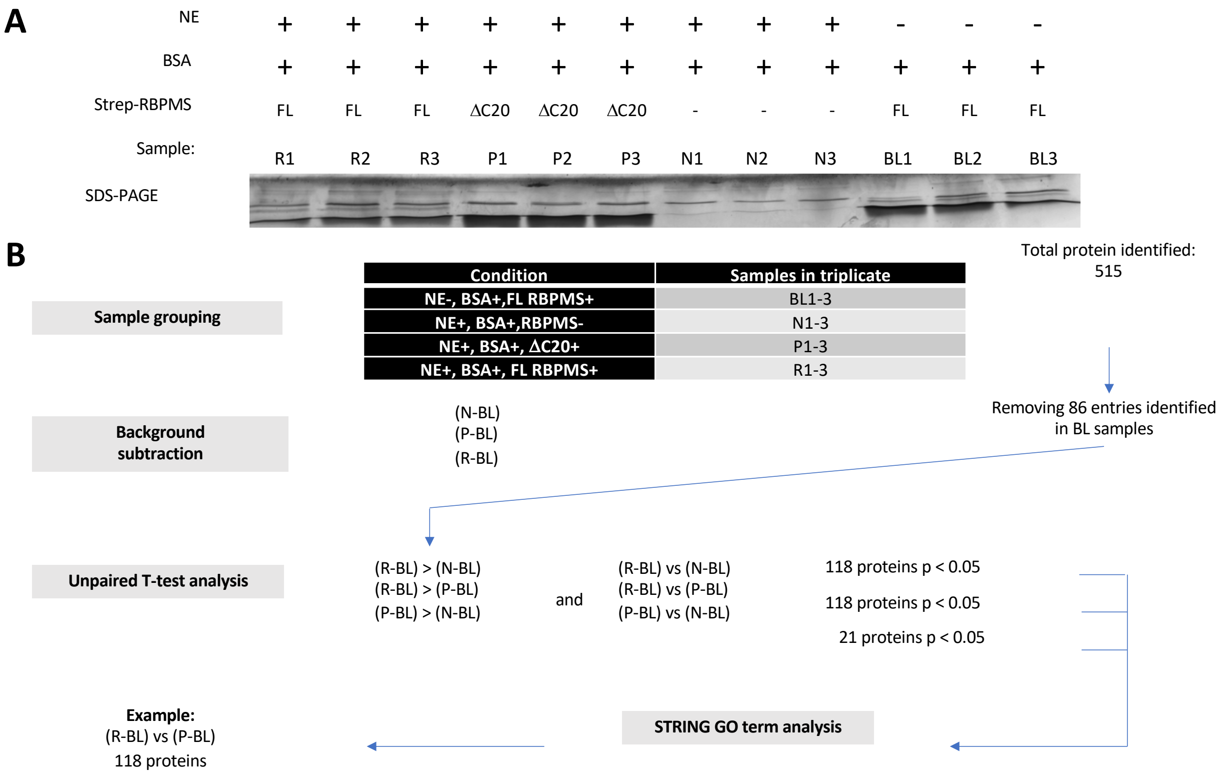

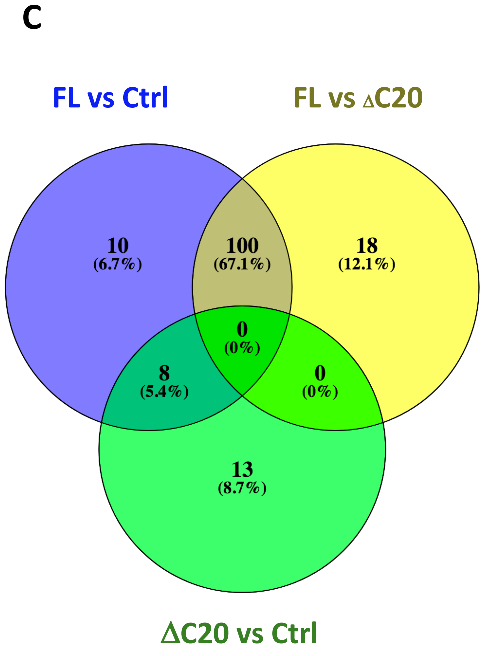

Figure S14. Sample preparation for affinity purification pull-down (AP-MS). (A)Coomassie stained SDS-PAGE gel lanes submitted to proteomic analysis. (B) Top panel: Flow chart summarizing the grouping, background subtraction, and statistical analysis strategies. The top table detailed the experimental design. Each group contains three technical repeats under the condition defined by four variables: NE (nuclear extract), BSA blocker, ΔC20 or FL RBPMS-A. Complete list of discovered proteins can be found in Supplementary files AP_MS_TSC_raw . The list of significantly differential enriched protein can be found in Supplementary files AP_MS_SL . (C) Venn diagram showing the overlap of RBPMS interactors enriched against different background conditions. Image generated with VENNY 2.1 server.

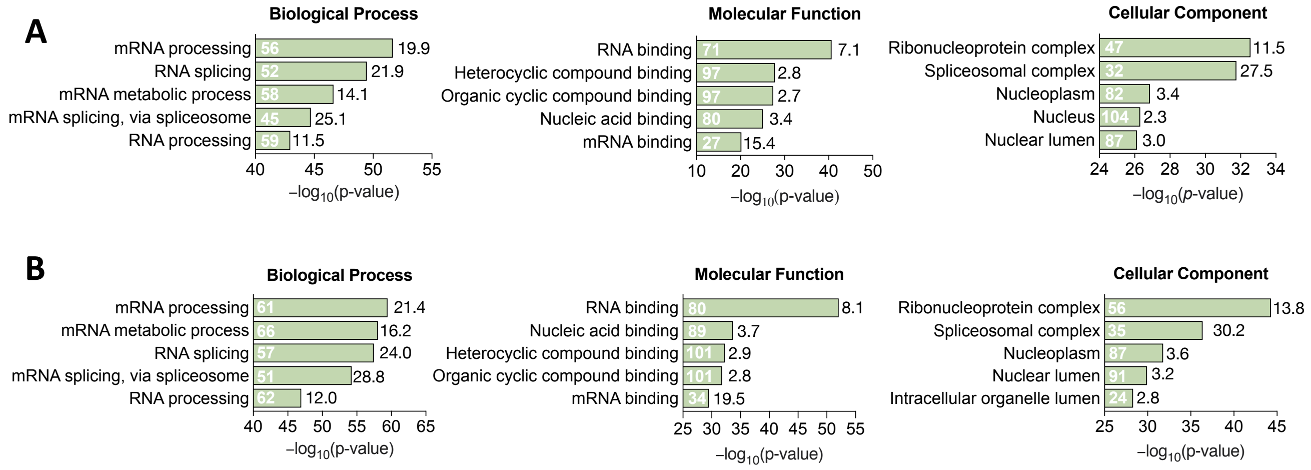

Figure S15. RBPMS-A interactome in Hela NE. (A) GO analysis of the 118 RBPMS-A interactors detectable above negative control. (B) GO analysis of the 133 RBPMS-A interactors significantly enriched compared to ΔC20 . The significance of enrichment for each term was evaluated using the right-sided hypergeometric test with Benjamini-Hochberg *p*-value correction. The top five enriched terms are shown for each GO category (biological process, molecular function, and cellular component). The number within the bar in white indicates the number of proteins annotated with a particular term. The number in front of the bar in black indicates the strength of the enrichment effect (i.e. the ratio between the number of proteins annotated with a term in an enriched network and the number of proteins expected to be annotated with this term in a random network of the same size).

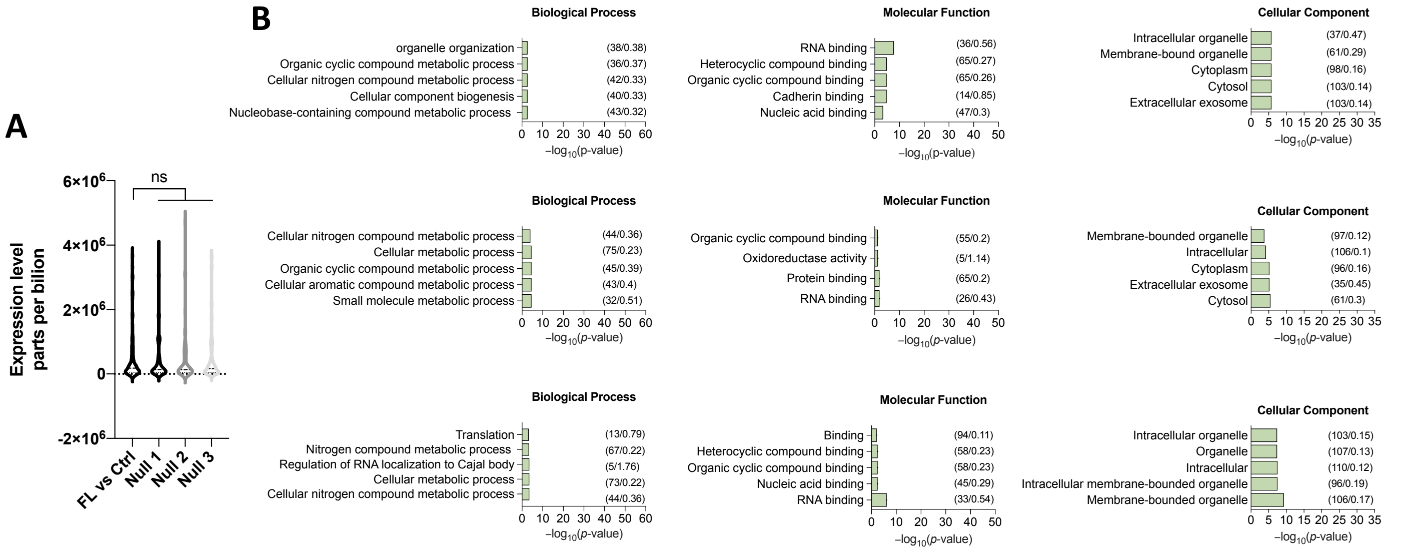

Figure S16. (A) Gene expression level comparison of three size-matched randomly sampled gene sets to the 118 significant FL vs Ctrl RBPMS-A interactors. Null-gene sets 1-3 were generated based on gene expression levels determined by proteomic method, Dorte B. Bekker-Jense *et al* 2017. Data accessed by EBI Expression Atlas with PRIDE project code PXD004452. The list of genes are provided in Supplementary files AP_MS_SL. (B) GO analysis of the Null gene set 1-3. The significance of enrichment for each term was evaluated using the right-sided hypergeometric test with Benjamini-Hochberg *p*-value correction. The top five enriched terms are shown for each GO category (biological process, molecular function, and cellular component). Two additional values associated with each term are provided and bracketed. The number in front of forward slash indicates the number of proteins annotated with a particular term. The number after the forward slash indicates the strength of the enrichment effect (i.e. the ratio between the number of proteins annotated with a term in an enriched network and the number of proteins expected to be annotated with this term in a random network of the same size).

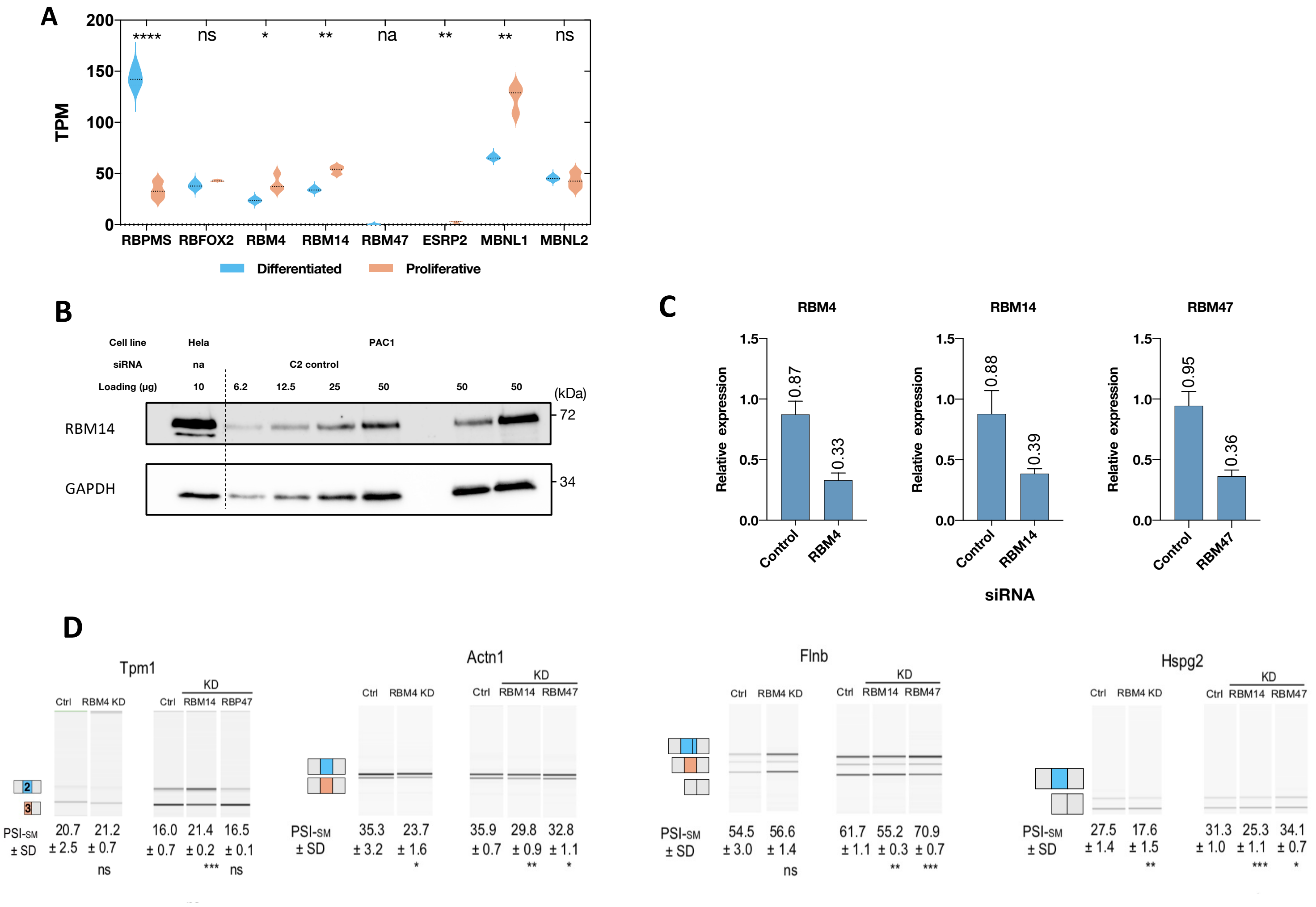

Figure S17. RBPMS-A and its associated proteins modulate VSMC alternative splicing events. (A) mRNA expression of RBPMS and its putative interaction partners in cultured smooth muscle cell PAC-1 in either differentiated, D, or proliferative, P, state. Median of the dataset is depicted as black dashed line. Data retrieved using NCBI Gene Expression Omnibus accession number GSE127799. (B) Western blots or (C) RT-qPCR verification of the knockdown of RBM14, RBM47 and RBM4 compared to control siRNA knockdown 48 hr after the second siRNA treatment. For qPCR, relative expression levels were normalised to housekeeping gene CANX and Rpl32. For Western blot, GAPDH detection was used as a loading control. (D) RT-PCR analysis of differentiated and proliferative exon usage upon knockdown with indicated siRNA. Illustrations of the splicing isoforms are shown on the left and indicate differentiated exon (blue) and proliferative exon (Salmon PSI, percentage of splice in, values for the SM exons are shown as mean PSI ± SD (n=3). Statistical analysis was performed using unpaired, two-tailed Student's T test, indicated as ns = p > 0.05, * = p < 0.05, ** = p < 0.01, *** = p< 0.001, and **** = p< 0.0001.

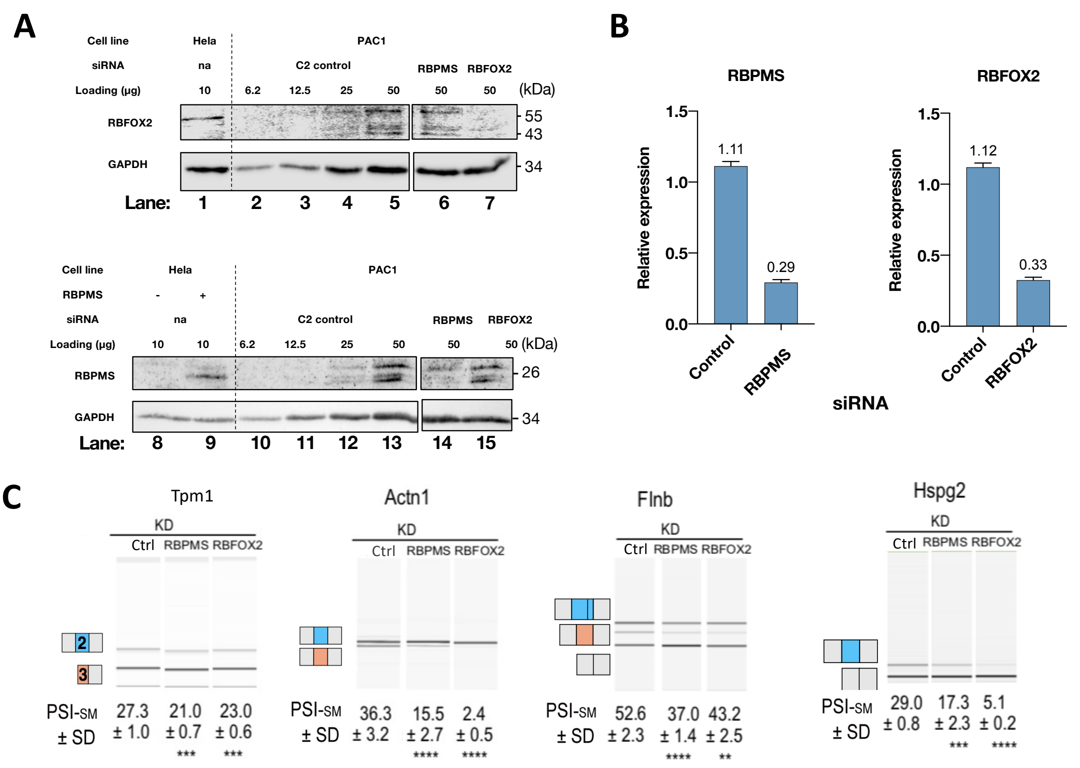

Figure S18. RBPMS-A and RBFOX2 modulate VSMC alternative splicing events. (A) Western blots or (B) RT-qPCR verification of the knockdown of RBPMS and RBFOX2 compared to control siRNA knockdown 48 hr after the second siRNA treatment. For qPCR, relative expression levels were normalised to housekeeping gene CANX and Rpl32. For Western blot, GAPDH detection was used as a loading control. Recombinant RBPMS was added to Hela extract to serve as immunoblotting positive control, Lane 9. (C) RT-PCR analysis of differentiated and proliferative exon usage upon knockdown with indicated siRNA. Illustrations of the splicing isoforms are shown on the left and indicate differentiated exon (blue) and proliferative exon (Salmon). PSI, percentage of splice in, values for the SM exons are shown as mean PSI ± SD (n=3). Statistical analysis was performed using unpaired, two-tailed Student's T test, indicated as ns = p > 0.05, * = p < 0.05, ** = p < 0.01, *** = p< 0.001, and **** = p< 0.0001. siRNA used: RBPMS KD1, and RBFOX2 KD1, see M&M.

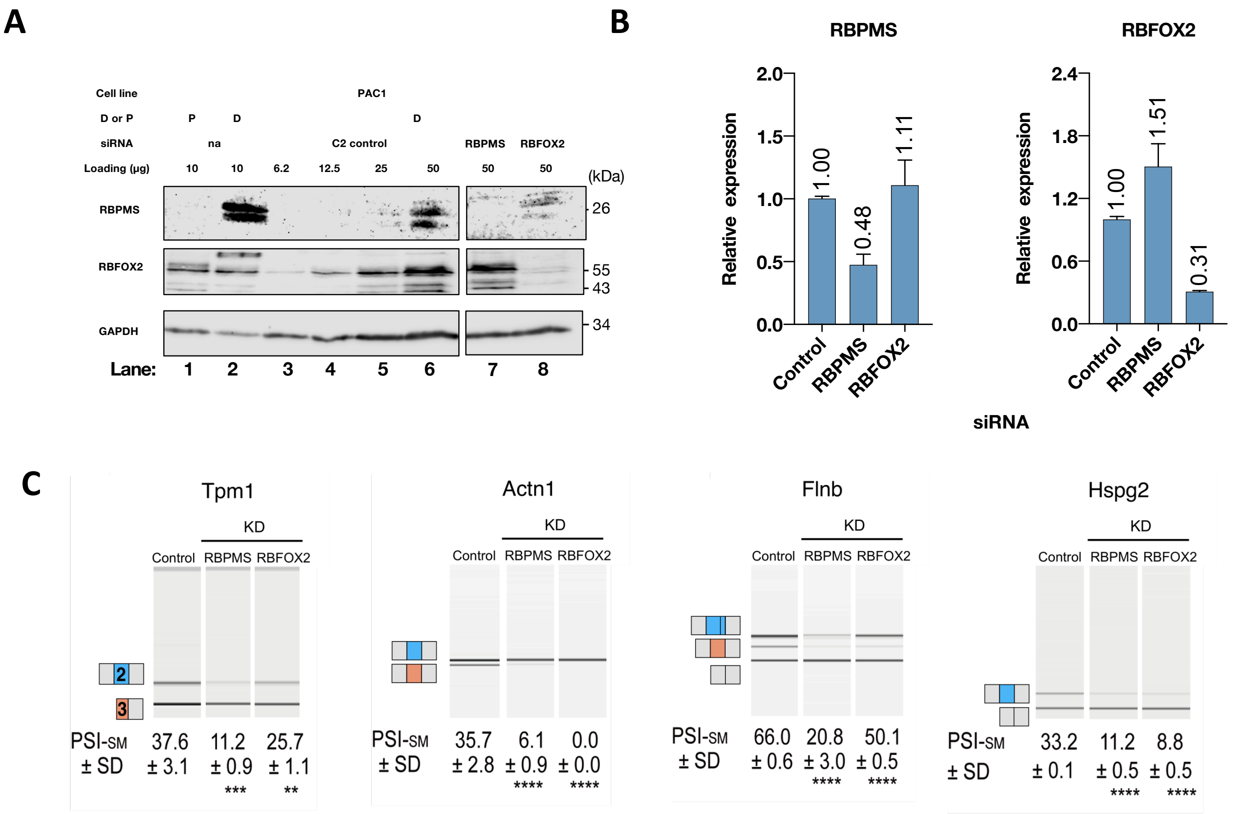

Figure S19. RBPMS-A and RBFOX2 modulate VSMC alternative splicing events. (A) Western blots or (B) RT-qPCR verification of the knockdown of RBPMS and RBFOX2 compared to control siRNA knockdown 48 hr after the second siRNA treatment. For qPCR, relative expression levels were normalised to housekeeping gene CANX and Rpl32. For Western blot, GAPDH detection was used as a loading control. Lane 1 and 2, proliferative “P” or differentiated “D” PAC1 lysate prepared from separately and served as positive controls. Lane 3-9 , lysate prepared from the same knockdown experiments. (C)) RT-PCR analysis of exon usage upon two siRNA treatments with indicated siRNA. Illustrations of the splicing isoforms are shown on the left and indicate differentiated exon (blue) and proliferative exon (Salmon). PSI, percentage of splice in, values for the SM exons are shown as mean PSI ± SD (n=3). siRNA used: RBPMS KD3, and RBFOX2 KD2, see M&M.

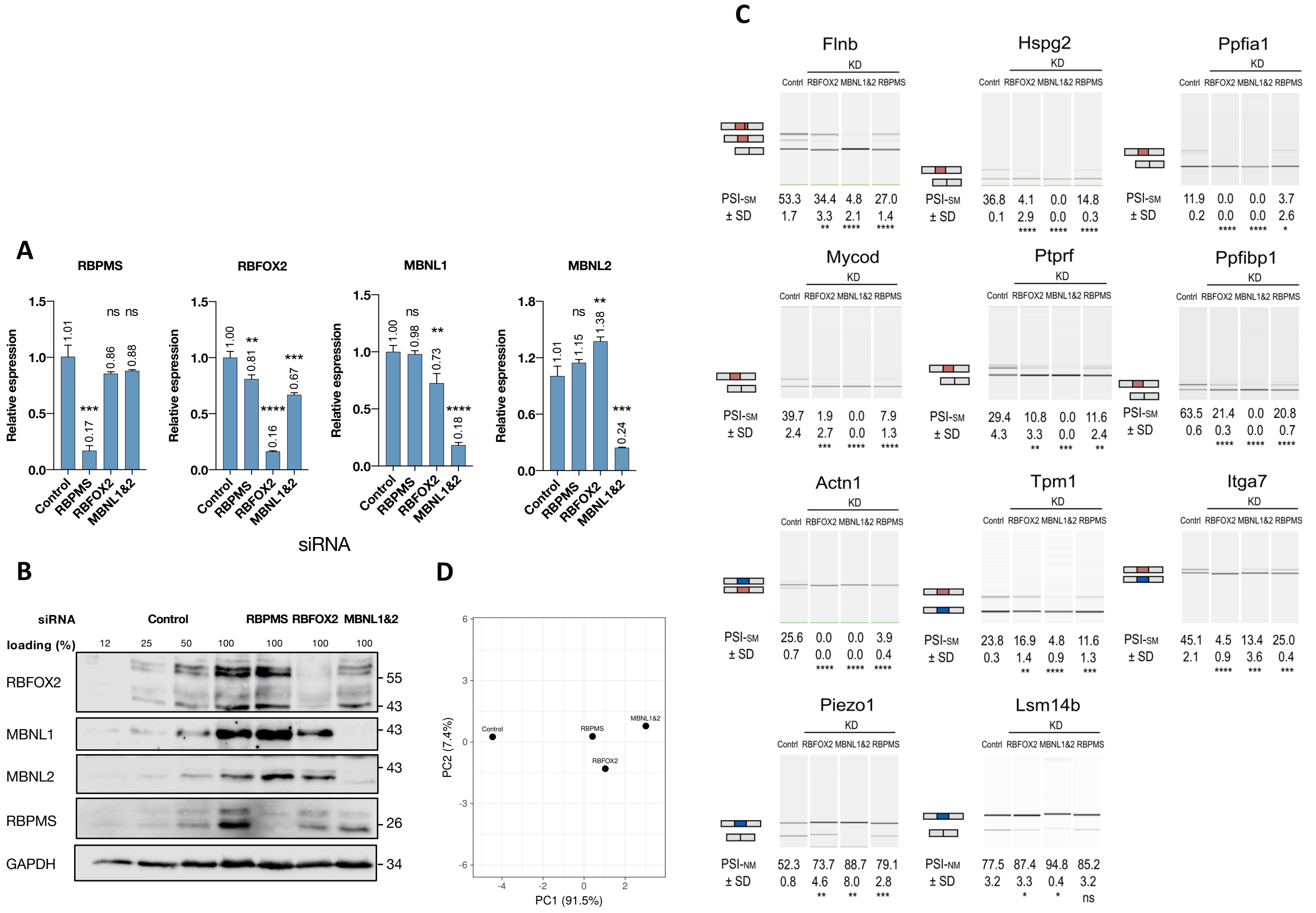

Figure S20. RBPMS-A and its associated proteins modulate VSMC alternative splicing events. (A-B) Upon two siRNA treatments, knockdown (KD) of RBPMS, RBFOX2, MBNL1 and MBNL2 was verified via RT-qPCR or Western blots. For qPCR, relative expression levels were normalised to housekeeping gene CANX and Rpl32. Mean and SD are indicated on top of the bar and using error bar. For Western blot, GAPDH detection was used as a loading control.(C) RT-PCR analysis of differentiated and proliferative exon usage upon two siRNA treatments with indicated siRNA. Illustrations of the splicing isoforms are shown on the left and indicate differentiated exon (blue) and proliferative exon (Salmon). PSI, percentage of splice in, values for the SM exons are shown as mean PSI ± SD (n=3). Statistical analysis was performed using unpaired, two-tailed for A-B and D, Student's T test, indicated as ns = p > 0.05, * = p < 0.05, ** = p < 0.01, *** = p< 0.001, and **** = p< 0.0001. For A, RBM47 expression in rat VSMC is insufficient for reliable statistical comparison. siRNA used: RBPMS KD1, MBNL1 THH2, MBNL2, RBFOX2 KD2, see M&M. (D) *X*-axis and *Y*-axis showing principal component 1 and principal component 2, which explain 91.5% and 7.4% of the total variance, respectively. Data separated based on the siRNA treatment. *N* = 4 data points.

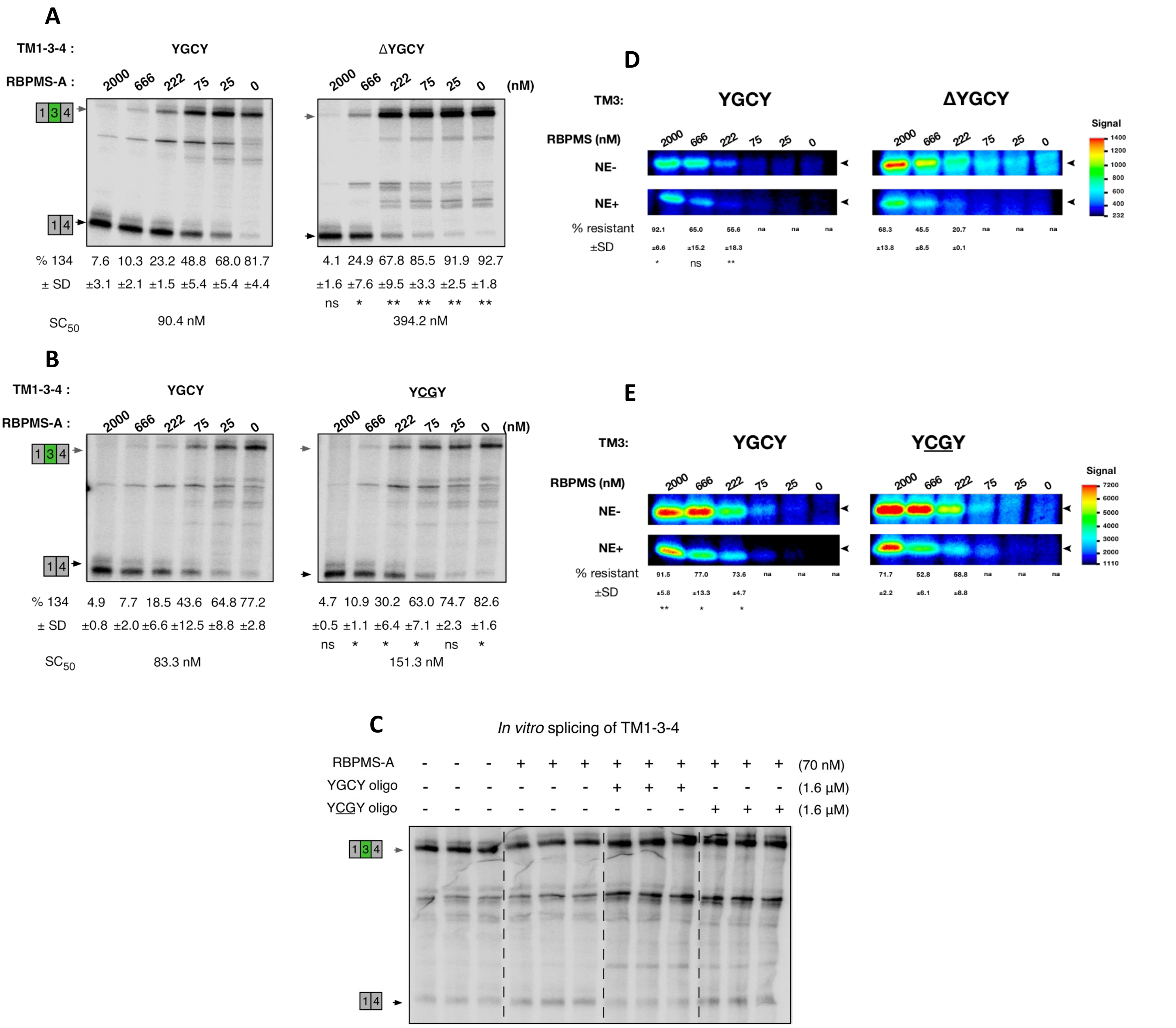

Figure S21. Validation of cooperativity between MBNL and RBPMS *in vitro*. Sensitivity of RBPMS modulated splicing switch on TM134 to the deletion (A) or mutation (B) of flanking MBNL binding sites. YGCY refers to WT transcript. ΔYGCY refers to deletion of both URE and DRE elements (Fig 2B). YCGY refers to transcript with MBNL binding site disrupted. Half maximum switching concentration, SC$_{50}$, was estimated with log (inhibitor) vs response-variable slope equation provided by GraphPad Prism 9. (C) Titration of RNA oligo containing three YGCY or YCGY motifs to the TM134 *in vitro* splicing reactions in combination with the effect of 70 nM of RBPMS-A. Comparison of RBPMS-A NE-resistant UV crosslinking to [^{32}P]-CTP labelled TM3 transcript between WT with the deletion (D) or mutation (E) of flanking MBNL binding sites. %$_{resistant}$, the percentage of binding that sustained NE competition, (Signal$_{NE+}$/Signal$_{NE-}$)100. Statistical analysis was performed using unpaired, one-tailed, Student's T test, indicated as ns = $p > 0.05$, * = $p < 0.05$, ** = $p < 0.01$, *** = $p< 0.001$, and **** = $p< 0.0001$. For D-E, signal to noise ratio between 25 -75 nM is too low to conduct confident comparison.

TEMPLATE	5'-3' SEQUENCES	METHOD	COMMENT	CONSTRUCT
**pET15b**	F:TCCGGCTGCTAACAAAGCCCGAAAGG<br>R:GGTTCTCGCCGCTGCTGTGATGATGATGATGATGGCT	PCR/LIC	LIC adaptor addition	pET15-LIC
**pEGFP-C1-RBPMS-A & pET15-LIC**	F:CACAGCAGCGGCGAGAACCTGTACTTCCAGGGAGTCGACAACGGCGGCGG C<br>R:AGCAGCCGGATCCTCGAGTTATTAGCAG	PCR/LIC	TEV-RBPMS insert	pET15b-TEV-RBPMS-A
**pEGFP-C1-RBPMS-A & pET15-TEV**	F:GATGTCGACAACGGCGGCGGCAAAG<br>R:GATCCTCGAGTTATTACTGGGCATGCAGTGAAGC	PCR/ SalI-XhoI		pET15b-TEV-RBPMS-A-ΔC20

Table S.1 Primers used in cloning of RBPMS constructs with TEV cleavable His_6 tag. LIC, ligation independent cloning; F, Forward ; R, Reverse

TEMPLATE	5'-3' SEQUENCES	METHOD	COMMENT	CONSTRUCT
**pET15b-TEV-RBPMS-A**	RBS sequences:<br>F:CTAGAATAATTTTGTTTAACTTTAAGAAGGAGATATACCATGGGCA GCAGC<br>R:CCCATGGTATATCTCCTTCTTAAAGTTAAACAAAATTATT<br>StrepII and His_6 tag:<br>F:TGGAGCCATCCTCAGTTCGAGAAAGGAGGTGGTGGCTCTCATCAT CATCATCA<br>TCACGGTGGTGGCTCTG<br>R:TCGACAGAGCCACCACCGTGATGATGATGATGATGAGAGCCACCA CCTCC<br>TTTCTCGAACTGAGGATGGCTCCAGCTGCTG	XbaI-SalI Oligo cloning	Replacing the N-terminal His_6-TEV with StrepII-His_6	pET15b-StrepII-His_6-RBPMS-A
**pET15b-StrepII-His_6-RBPMS-A**	F:GATGTCGACAACGGCGGCGGCAAAG<br>R:GATCCTCGAGTTATTACTGGGCATGCAGTGAAGC	PCR/ SalI-XhoI		pET15b-StrepII-His_6-RBPMS-A-ΔC20
**pET15b-StrepII-His_6-RBPMS-A**	F:GATGTCGACAACGGCGGCGGCAAAG<br>R:GATCCTCGAGTTATTATGGAGTCCCTACG	PCR/ SalI-XhoI		pET15b-TEV-RBPMS-A-RRM
**pEGFP-C1-RBPMS-A-K100E & pET15b-StrepII-His_6-RBPMS-A**	Fragment 1:<br>F:CGACTAGAGTTTGCTGAGGCAAACACGAAGATG<br>R:GGGTTGCCTTACTGGTTAGCAGAATG<br>Fragment 2:<br>F:CATTCTGCTAACCAGTAAGGCAACCC<br>R:CATCTTCGTGTTTGCCTCAGCAAACTCTAGTCG	Gibson assembly		pET15b-TEV-RBPMS-A-K100E

Table S.2 Primers used in cloning of RBPMS constructs with N-terminal StrepII and His_6 tags. RBS, ribosome binding sequence ; F, Forward ; R, Reverse

Primer	5'-3' SEQUENCES	METHOD
CAC 1.a/b	F:GGCGCGCGGTGTGGCCCTGCCCACGAATGGCTAACTTTC	Mutagenesis
	R:GAAAGTTAGCCATTCGTGGGCAGGGCCACACCGCGCGCC	PCR
CAC 2.a/b	F;CCTTTATGGTCTACGCCCCCTCAACCCGCCCCTTGCGGGATCACG	Mutagenesis
	R:CGTGATCCCGCAAGGGGCGGGTTGAGGGGGCGTAGACCATAAAG G	PCR
CAC 3.a	F:GCCCCTTGCGGGATCCCGCTGCCTGCTGCATC	Mutagenesis
	R:GATGCAGCAGGCAGCGGGATCCCGCAAGGGGC	PCR
CAC 3.b	F:CTGCCTGCTGCA TCCCCCCCCCTTCCCCCTTC	Mutagenesis
	R:GAAGGGGGAAGGGGGGGGGATGCAGCAGGCAG	PCR
CAC 4.a/b	F:CCCCCTTCCTTCCCCCCCCCCGTACTCCCCTGCCAACTCCCAGC	Mutagenesis
	R:GCTGGGAGTTGGCAGGGGAGTACGGGGGGGGGGAAGGAAGGG GG	PCR
CAC 5.a/b	F:GGGTGAGGGAGGCAGGGGGAGCGGGCACTTACATCTGTG	Mutagenesis
	R:CACAGATGTAAGTGCCCGCTCCCCCTGCCTCCCTCACCC	PCR
CAC 8.a/b	F:CTATGGCCAGGCTGGGGGCAGGGGTCCTGTAGGAGGA	Mutagenesis
	R:TCCTCCTACAGGACCCCTGCCCCCAGCCTGGCCATAG	PCR

Table S.3 Primers used in the depletion of tandem CAC sites; F, Forward primer; R, Reverse primer

Complementary DNA oligo	5'-3' SEQUENCES	METHOD
GAPDH mRNA	GAGGTCAATGAAGGGGTC AT	Complex assembly sensitivity & Psoralen crosslink digestion
U1 snRNA 1-15nt	CTGCCAGGTAAGTAT	Complex assembly sensitivity
U2 snRNA 1-15nt	AGGCCGAGAAGCGAT	Complex assembly sensitivity
U2 snRNA 18-42nt	CAGATACTACACTTG	Complex assembly sensitivity
U1 snRNA 64-75nt	CGGAGTGCAATG	Psoralen crosslink digestion
U2 snRNA 104 112nt	CCTGCTCCA	Psoralen crosslink digestion
U2 snRNA 159-171nt	AGGTACTGCAATA	Psoralen crosslink digestion

Table S.4 List of complementary DNA oligonucleotide used in combination with RNase H

TEMPLATE	5'-3' SEQUENCES	METHOD	CONSTRUCT
pGEM-TM134	F:CCCAAGCTTGCCAGCACTTCAGTCCTAGCCAGTTCCTGAAGGTACCACCCTCACTTTCTCCCCAGCTCTGAG<br>R:CGCGGATCCACTTACCCAGACGCTCCTGAGCGC	HindIII-BamHI	pGEM-TM4-40exU1
pGEM-4Z & Synthetic oligonucleotide	Fragment 1:<br>F:AGCTTACGGAATTCGAGCTCGCCCACTCTTGGATCGGAAACCCGTCGGCCTCCGAACGGTAAGAGCCTAGCATGTAGAACTGGTTACC<br>R:AGTTCTACATGCTAGGCTCTTACCGTTCGGAGGCCGACGGGTTTCCGATCCAAGAGTGGGCGAGCTCGAATTCCGTA<br>Fragment 2:<br>F:TGCAGCCCAAGCTTGCTGCACGTCTAGGGCGCAGTAGTCCAGGGTTTCCTTGATGATGTCATACG<br>R:GATCCGTATGACATCATCAAGGAAACCCTGGACTACTGCGCCCTAGACGTGCAGCAAGCTTGGGCTGCAGGTAACC	HindIII-BamHI	pGEM-AML-E1
pGEM-TM234 & pGEM-TM3	-	AccI -HindIII	pGEM-TM23
p3U3D-ΔUΔD & pGEM-TM134 & pGEM-TM3	Fragment 1:<br>F:GGTCTACGCACCCTCAACCC<br>R:GGGGAGGAGGTAAAGAAGAGG<br>Fragment 2:<br>F:CCTCTTCTTTACCTCCTCCCC<br>R:GGGTTGAGGGTGCGTAGACC	Gibson assembly	pGEM-TM134-ΔUΔD & pGEM-TM3-ΔUΔD
pGEM-TM134 & pGEM-TM3	Up stream "U" site:<br>F:CACCTTGCGGGATCACGCTCCCTCGTGCATCCCAC<br>R:GTGGGATGCACGAGGGAGCGTGATCCCGCAAGGTG<br>Down stream "D" site:<br>F:CCATAGCCCAGAGCACTGGATCCCGCCTCTCGTCGTGCGCACATTT<br>R:AAATGTGCGCACGACGAGAGGCGGGATCCAGTGCTCTGGGCTATGG	Mutagensis PCR	pGEM-TM134-YCGY & pGEM-TM3-YCGY
pGEM-4Z & pGEM-TM134	F:ATGCAGCAGGCAGCGTGATCCCGCAAGGTGCGGGTTGAGGGTGCGTAGACCAT AAGCTTGTCTCCCTATAGTGAGTCGTATTAG<br>R:CCCACCCCCTTCCCCCTTCCTTCCCCCCACCCCCGTACGCCACTGCCAACTCCCA GCATGCCTGCAGGTCGACTC	HindIII & PCR	pGEM-P3CAC
pGEM-P3CAC & pGEM-TM134-ΔCAC	Fragment 1:<br>F:GACTCACTATAGGGAGACAAGC TTATGGTCTACGCCCCCTC<br>R:CTAGAGTCGACCTGCAGGCATGCTGGGAGTTGGCAGGG<br>Fragment 2:<br>F:GCTTGTCTCCCTATAGTGAGTC<br>R:ATGCCTGCAGGTCGACTCTAG	Gibson assembly	pGEM-P3CCC
pGEM-4Z	F:AAGCTTGTCTACGCACCCTCAACCCGCACCTTGCGGGATG<br>R:GATCCATCCCGCAAGGTGCGGGTTGAGGGTGCGTAGACA	HindIII-BamHI	pGEM-sCAC
pGEM-4Z	F:AGCTTGTCTACGCCCCCTCAACCCGCCCCTTGCGGGATG<br>R:GATCCATCCCGCAAGGGGCGGGTTGAGGGGGCGTAGACA	HindIII-BamHI	pGEM-sCCC
pGEM-4Z	Fragment 1:<br>F:TGGCCTCTTTCGTTGGCCTCGTGTGTCGAATTCGTATTCTATAGTG<br>R:GTTCATCCATAGTTGCCTGACTCC<br>Fragment 2:<br>F:GGAGTCAGGCAACTATGGATGAAC<br>R:ACGAGGCCAACGAAAGAGGCCAGGAGGAAGCTTGTCTCCC	Gibson assembly	pGEM-NCD12

Table S.5 Cloning oligos for splicing substrates; F, Forward primer ; R, Reverse primer

PRIMER	5’-3’ SEQUENCES	METHOD
E3 extend	CATCTGTGGCCTTTTTCTCCGCCAG C	Reverse transcription
E4 extend	CCAGACGCTCCTGAGCGCG	Reverse transcription
Tpm1 exon 2 For	AGGAGGACATCTCAGCAA	PCR
Tpm1 exon 3 For	GAGCTGGCGGAGAAAAAG	PCR
Tpm1 exon 4 Rev	TCCAACTCCTCCTCAACCAG	PCR
AML E1 for	CTCTTGGATCGGAAACCCG	PCR
TM E3 Rev	CCTTTTTCTCCGCCAGCTCC	PCR

Table S.6 List of primers used in *trans* splicing product detection

Experiment	Plasmid	Restriction enzyme	Capping %
Cis-splicing	pGEM-TM134 &-YCGY &-ΔUΔD	XbaI	50
	pGEM-TM234	BamHI	50
Complex assembly & Trans-splicing UV crosslinking	pGEM-TM3 &-YCGY &-ΔUΔD	EcoRI	80
	pGEM-TM23	EcoRI	80
RNA affinity pull-down	pGEM-TM3-MS2	EcoRI	80
Trans-splicing	pGEM-TM4-40exU1	BamHI	80
	pGEM-AML-E1	BamHI	80
Band shift	pGEM-P3CAC & -P3CCC	XbaI	0
	pGEM-SCAC & -SCCC	EcoRI	0
MBNL compete	pGEM-D12	BamHI	0
	pGEM-NCD12	BamHI	0

Table S.7 Linearized template plasmid for *in vitro* transcription

Gene and identifier	Source or reference	5’-3’ sequence
C2	Dharmacon Gooding et al., 2013	AAGGUCCGGCUCCCCCAAAUG
RBM4 A	Ambion Life technology 4392420	GACUUGACCGAGCAAUAUA
RBM4 B	Sigma Aldrich SASI_RN01_00120943	CAGACUUUACUGAACAGUA
RBM14	Ambion Life technology 4392420	GCAGCUCAACGGCAAAGAA
RBM14	Sigma Aldrich SASI_RN02_00337825	GCGUUUAGCCGAGCUCUCUtt
RBM47	Sigma Aldrich SASI_RN01_00068041	CCGGUUACAACAUGGUACAtt
RBPMS KD1	Nakagaki-silva et al 2019 RSS363828	GGCGGCAAAGCCGAGAAGGCGAACA
RBPMS KD3	Nakagaki-silva et al 2019	CAGUACUCCUCUGCCCAACACUGUA
MBNL1 THH2	Dharmacon Gooding et al., 2013	CACGGAAUGUAAAUUUGCAUU
MBNL2	Dharmacon Gooding et al., 2013	GAAGAGUAAUUGCCUGCUUUU
RBFOX2	KD1	GCAAUGGUUCAGCCUUUUA
RBFOX2	KD2	AGAAGAUGGUCACACCAUA
ESRP2	Sigma Aldrich SASI_RN02_00228675	GCUAUAUUGAGGUGUAUAAtt
ESRP2	Ambion Life technology 4390815	GACUUAAUCCUCCUAGUUU
ESRP2	Sigma Aldrich SASI_RN02_00228676	CAGAAAUGGUGAGGCCCUUtt

Table S.8 List of siRNA

Gene	Genome	Regulated Exon or Transcript Reference	5'-3' sequence	Experiment
TPM1	hg19	Chr15:63335905-63336030(+)	GAGCTGGCAGAGAAAAAG<br>AGGAGGACATCGCGGCCA<br>TCCAACTCTTCCTCAACCAG	RT-PCR
Tpm1	rn6	Chr8:72840038-72840164(-)	AGGAGGACATCTCAGCAA<br>GAGCTGGCGGAGAAAAAG<br>TCCAACTCCTCCTCAACCAG	RT-PCR<br>PvuII-HF
Actn1	rn6	Chr6:103379819-103379900(-)	CAGATCCTGACCCGGGATG<br>CATGACTTGGTCTGCTGTATCTGT	RT-PCR
Flnb	rn6	Chr15:18780269-18780341(-)	CAGGGAAGGGGAAAGTCACC<br>ACTCACTGGGACATAGGCCT	RT-PCR
Hspg2	rn6	Chr5:155853492-155853543(+)	CTGGGGGTTCAAGTTCCGAC<br>CGTGCAGACTCTTGGGAACT	RT-PCR
Ptprf	rn6	chr5:137045508-137048755	CGTCAAAGGATGAGCAGTCAATC<br>CCCATCATTGGCTTTGAGAC	RT-PCR
Pizeo1	rn6	chr19:55313339-55314234	CAACTCCAGTCCACAGACCC<br>TTCCTCCTCACTGTCCGACT	RT-PCR
Itga7	rn6	chr7:3362502+3364542	GGGGAGTGGAAGTTCTGTGA<br>CCTTCCCAGAATCAATGGAG	RT-PCR
Mycod	rn7	Chr10:49882268+49891909	CAGTTACGGCTTCAACAGAGAA<br>TTTTCCTCGGGTCATGGAAC	RT-PCR
Ppfibp1	rn6	chr4:181393665+181396223	GACGAAAGGAGAAGGGGTCG<br>CCATCAGAGACTCCACTGCC	RT-PCR
Ppfia1	rn6	chr1:217679927-217681616	GCCAGTTGCAAGAACGTCTG<br>TTGCCATGTCTCTCCTCAGC	RT-PCR
Lsm14b	rn6	chr3:175415210+175415825	GCTAAGAAGCTGTTGCCCAG<br>GCGATTTTGCCCTCTGGAAC	
RBM4	rn7	NM_001170484.1	TGCTGCTGCTGCTTGTACTG<br>CACACACATCCCACCTCAAAC	RT-qPCR
RBM14	rn7	NM_133388_1	TCCCAATTCCCTATCTCCCC<br>ACCACTCCAATCCCACATC	RT-qPCR
ESRP2	rn7	NM_001107423.1	TACCTCACCACACCTCCTAC<br>TCCTCTTCTACAAACACACCC	RT-qPCR
RBM47	rn7	NM_001005882.1	ACGAAGGCAAGGGCAAACAG<br>TACCGCAGGAATGACAGCAG	RT-qPCR
RBFOX2	rn7	NM_001261516	GCCTACAGATATGCACAGCCT<br>CTGTGTACACCCTGCCGTAA	RT-qPCR
RBPMS	rn7	NM_001271244.1	AAAGCCGAGAAGGAGAACACC<br>TTGAATGGCCTGAAGAGCAG	RT-qPCR
MBNL1	rn7	NM_001191566.1	TGAAAATGGACGAGTAATCGCC<br>AAACATTGGCACGGGCTGCAG	RT-qPCR
MBNL2	rn7	NM_001111064.1	GGCTCAACTGCAACTCAGAAAC<br>GTCGTTTGTGTCGATCATGG	RT-qPCR
Rpl32	rn7	NM_013226.3	GCCCAAGATCGTCAAAAAGAGG<br>ATCAGGATCTGGCCCTTGAATC	RT-qPCR
CanX	rn7	NM_172008.2	TGAAGCTGCTATCCAAGACCTC<br>TGGGGTTTTTGTGGCGAAAG	RT-qPCR

Table S.9 PCR primers for AS analysis in knockdown experiments

ANTIBODIES	HOST	SOURCE	DILUTION
ESRP2	Rabbit	Abcam ab113486	1:1000
MBNL1	Mouse	SigmaAldrich M3320	1:2000
MBNL2	Rabbit	Santa Cruz, sc-134813	1:500
GAPDH	Rabbit	Abcam ab181602	1:10,000
hnRNP M	Rabbit	Abcam, ab177957	1:10,000
Matrin3	Goat	Santa Cruz, sc-55723	1:1000
PTBP1	Mouse	Douglas Black lab	1:2000
RBFOX2	Rabbit	Bethyl, A300-864A	1:2000
RBM14	Rabbit	Abcam, ab70636	1:2000
RBM4	Rabbit	Abcam, ab251923	1:2000
RBM47	Rabbit	Abcam, ab154176	1:2000
RBPMS	Rabbit	SigmaAldrich HPA056999	1:500
Strep-tag II	Mouse	IBA Lifesciences, 2-1507-001	1:20,000
Tubulin	Rat	Abcam, ab6160	1:5000

Table S.10 List of antibody

	MBNL Competing oligonucleotide	Band shift	Cis Splicing substrates	Complex assembly & Psoralen crosslinking	UV Crosslinking	Trans splicing Substrates & RNA affinity pull-down
Capping % (Radio-specificity)	0 (low)	0 (low)	50(low)	80(low)	80(high)	80(no)
Linearised RNA 1 mg/ml (µl)	1	1	1	1	1	1
1 µl of 5 mM A,U,GTP (x mM CTP)	1 µl of 10 mM NTP	5(2)	5(2)	5(3)	5(0.4)	10 µl of 10 mM A,U,CTP (2 mM GTP)
Ribolock RNase Inhibitor (µl)	0.4	0.4	0.4	0.4	0.4	1
5x transcription buffer (µl)	2	2	2	2	2	10
[32P] CTP (µl)	1	1	1	1	2	-
10 mM Cap analogue (µl)	-	-	0.5	0.8	0.8	8
T7 RNA polymerase (µl)	1	1	1	1	1	1.5
H2O	3.6	3.6	3.1	2.8	1.8	18.5

Table S.11 *In vitro* transcription
